# Model-driven discovery of calcium-related protein-phosphatase inhibition in plant guard cell signaling

**DOI:** 10.1371/journal.pcbi.1007429

**Published:** 2019-10-28

**Authors:** Parul Maheshwari, Hao Du, Jen Sheen, Sarah M. Assmann, Reka Albert

**Affiliations:** 1 Department of Physics, Penn State University, University Park, Pennsylvania, United States of America; 2 Department of Molecular Biology and Centre for Computational and Integrative Biology, Massachusetts General Hospital, and Department of Genetics, Harvard Medical School, Boston, Massachusetts, United States of America; 3 Biology Department, Penn State University, University Park, Pennsylvania, United States of America; Purdue University, UNITED STATES

## Abstract

The plant hormone abscisic acid (ABA) promotes stomatal closure via multifarious cellular signaling cascades. Our previous comprehensive reconstruction of the stomatal closure network resulted in an 81-node network with 153 edges. Discrete dynamic modeling utilizing this network reproduced over 75% of experimental observations but a few experimentally supported results were not recapitulated. Here we identify predictions that improve the agreement between model and experiment. We performed dynamics-preserving network reduction, resulting in a condensed 49 node and 113 edge stomatal closure network that preserved all dynamics-determining network motifs and reproduced the predictions of the original model. We then utilized the reduced network to explore cases in which experimental activation of internal nodes in the absence of ABA elicited stomatal closure in wet bench experiments, but not in our in silico model. Our simulations revealed that addition of a single edge, which allows indirect inhibition of any one of three PP2C protein phosphatases (ABI2, PP2CA, HAB1) by cytosolic Ca^2+^ elevation, resolves the majority of the discrepancies. Consistent with this hypothesis, we experimentally show that Ca^2+^ application to cellular lysates at physiological concentrations inhibits PP2C activity. The model augmented with this new edge provides new insights into the role of cytosolic Ca^2+^ oscillations in stomatal closure, revealing a mutual reinforcement between repeated increases in cytosolic Ca^2+^ concentration and a self-sustaining feedback circuit inside the signaling network. These results illustrate how iteration between model and experiment can improve predictions of highly complex cellular dynamics.

## Author summary

We build on the foundation of a comprehensive discrete dynamic model of a plant signaling network that governs the closing of microscopic stomatal pores on the surface of leaves. That network captured the vast majority of experimental observations regarding closure in response to a specific signal, the hormone abscisic acid, which elicits stomatal closure during drought, thereby promoting plant water conservation. Here, we identify a knowledge gap in understanding experimentally observed stomatal closure in response to activation of internal nodes in the network. In contrast to the expectation that these nodes might be positioned along a linear pathway, we find that the knowledge gap can be filled by reinforcement of a feedback-rich subnetwork. Specifically, we predict that cytosolic calcium elevation can inhibit one or more of three 2C-type protein phosphatases, and confirm this prediction experimentally. The model augmented with this new information recapitulates experimental observations on internal node activation, via a mechanism that is potentially generalizable to other biological networks. Key to this mechanism is a mutual reinforcement between two feedback loops, one that generates a repetitive activity pattern, and another that preserves a sustained activity pattern.

## Introduction

Stomatal pores on the surfaces of aerial plant parts mediate both uptake of carbon dioxide (CO_2_), the substrate for photosynthesis, and transpirational water vapor loss. The aperture of each stomatal pore is increased or decreased by dynamic swelling or shrinking, respectively, of the surrounding guard cell pair. Given the central role that stomatal apertures play in control of both carbon assimilation and plant water status, guard cells have evolved exquisite sensitivity to a plethora of environmental and endogenous signals. In particular, drought and other dehydrating stresses result in abscisic acid (ABA) synthesis and redistribution that comprises a strong stimulus for both inhibition of stomatal opening and promotion of stomatal closure.

Guard cell researchers can directly observe responses to ABA in the form of electrophysiological measurements of the ionic fluxes that drive guard cell volume changes, microscopy-based direct observations of stomatal apertures, and whole-leaf as well as whole-canopy measurements of alterations in CO_2_ and water vapor exchange with the atmosphere. Owing to these advantages, as well as to the vital role of this specialized cell type in the control of plant water use efficiency, guard cells have become a leading system for elucidation of cellular signaling processes [1–3]. A core ABA response system in guard cells consists of ABA binding to the soluble pyrabactin resistance 1/pyrabactin resistance 1-like/regulatory component of ABA receptor (RCAR/PYR1/PYL) receptors that inhibit protein phosphatase 2Cs (PP2Cs), thus activating the Ca^2+^-independent protein kinase OPEN STOMATA 1 (OST1). Resultant activation of nicotinamide adenine dinucleotide phosphate (NADPH) oxidases results in reactive oxygen species (ROS) production that enhances Ca^2+^ uptake. Dozens of downstream processes, both Ca^2+^-dependent and Ca^2+^-independent, ultimately activate anion channels; consequent anion loss causes membrane depolarization that drives K^+^ efflux, and the net loss of ions drives water efflux, guard cell shrinkage and stomatal closure.

In total, over 80 distinct signaling entities, including enzymes, signaling proteins, metabolites, cytosolic pH (pH_c_), cytosolic Ca^2+^ (Ca^2+^_c_), and cellular redox status are implicated by experimental analyses as components in ABA-induced stomatal closure. This is a massive amount of information, and computational approaches can aid in its comprehension and synthesis. We recently published an updated network of ABA-induced stomatal closure based on synthesis of information from over 100 scientific publications. The network contains 81 components (nodes) and 153 connections (edges) [4]. While networks themselves are static entities, they can form the basis of dynamic modeling approaches that can provide both analysis and predictions. Appropriate choice of modeling approaches requires recognition of the types of experimental data and their limitations. For example, in the case of ionic fluxes, detailed quantitative information in real time is available, making quantitative kinetic modeling approaches such as those invoked by the OnGuard software appropriate [5–7]. However, in the case of guard cell signaling networks, much of the information is qualitative rather than quantitative. In such instances, discrete models, in which components are designated to exist in a discrete, rather than continuous, number of states, are more appropriate, of which the simplest is the Boolean ON/OFF formalism. In this formalism the ON state (alternatively denoted as 1) is interpreted as a higher-than-threshold abundance or activity, and the OFF or 0 state is interpreted as lower-than-threshold abundance and activity.

In our previous model [4], we synthesized published knowledge concerning the mechanisms by which guard cell components regulate, and are regulated by, each other during ABA signaling, resulting in 58 Boolean regulatory functions that describe this regulation with the Boolean operators AND, OR, and NOT. We established initial (resting) node states where known, randomized states of the remaining nodes, and performed dynamic modeling in both the presence and absence of ABA. Because little is known concerning the relative temporal relationships of activation/deactivation among the majority of the 80+ signaling components, which function in highly interconnected signaling pathways, our modeling employed randomized asynchronous update, in which nodes are updated sequentially in a randomly selected order. We performed a large number of simulations (considering a large number of in silico stomata) and determined the percentage of simulations wherein the final state (attractor) representing stomatal closure was reached. Taking advantage of the ease by which components can be manipulated in silico, we also predicted the impact of knock out or constitutive activation (as can occur via pharmacological intervention or overexpression of an active protein) of every node in the network. Our previous model had 2^81^ possible system states (i.e., more than 10^23^) and thus was far from determined by the available wet bench data. Nevertheless, we observed that modeling predictions were congruent with experimental evidence in 85 out of 112 cases (76%), supporting the validity of our model and the usefulness of discrete dynamic modeling for complex non-linear systems, including ABA-induced stomatal closure.

From the above model, 9 of the predictions that were inconsistent with experimental data were simulations of stomatal behavior upon constitutive activation of an internal node in the absence of ABA. For example, while the model correctly predicted that elevation of ROS would lead to stomatal closure even in the absence of ABA, which is well-documented experimentally [8–10], the model failed to correctly predict that elevation of Ca^2+^_c_ would lead to stomatal closure in the absence of ABA, even though manipulations that elevate Ca^2+^_c_ are confirmed strong drivers of stomatal closure [11–15]. We determined that for 8 of these 9 cases, the discrepancy with experimental results would be eliminated if we made just one additional assumption, namely that the four PP2Cs with a documented role in guard cell ABA signaling are inhibited by elevation of Ca^2+^_c_ levels. These four PP2Cs are ABSCISIC ACID INSENSITIVE 1 (ABI1), ABSCISIC ACID INSENSITIVE 2 (ABI2), HYPERSENSITIVE TO ABA 1 (HAB1) and PROTEIN PHOSPHATASE 2CA (PP2CA). In the present report, we reduce our original model into a smaller network that preserves all the relationships of the complete network while being intuitively more accessible. We then evaluate, both computationally and experimentally, the previously posed hypothesis that Ca^2+^_c_ either directly or indirectly inhibits guard cell PP2Cs. Our analysis also highlights the network motif whose activation underlies the efficacy of the observed closure stimuli. Deeper analysis of the relationship of this motif with Ca^2+^_c_ oscillations provides insight into how various regular or irregular Ca^2+^_c_ patterns can affect stomatal closure.

## Results

### Model reduction

Our published network model of ABA-induced stomatal closure contains 81 nodes, 153 edges, and 58 regulatory functions (23 nodes do not have regulators) [4]. The full biological names corresponding to the abbreviated node names of this network are reproduced in S1 Table. To ease comprehension of our new analyses, we employ dynamics-preserving methods to reduce this network [16–18]. These manipulations are possible given the inherent logic structure of Boolean networks, can be implemented for any Boolean system, and have no impact on the outcomes of dynamic modeling. All network reduction processes that were implemented are described in the Methods section and in S2–S5 Tables. To give an example of one type of reduction, two nodes related by an equivalence relationship can be merged. That is, given an edge A → B such that A is the sole regulator of B and B is the only target of A, the state of B will equal the state of A in every long-term state (steady state) of the system. Because of this strong determination between node A and node B, they can be merged into a single equivalent node AB. The steady state of this merged node reflects the shared steady state of node A and B, and it also equivalently expresses interventions into nodes A or B. For example, since knockout of node A causes the OFF state of node B, the knockout of node AB in the reduced network is equivalent to the knockout of A, and also to the knockout of B, in the original system.

**Fig 1:**
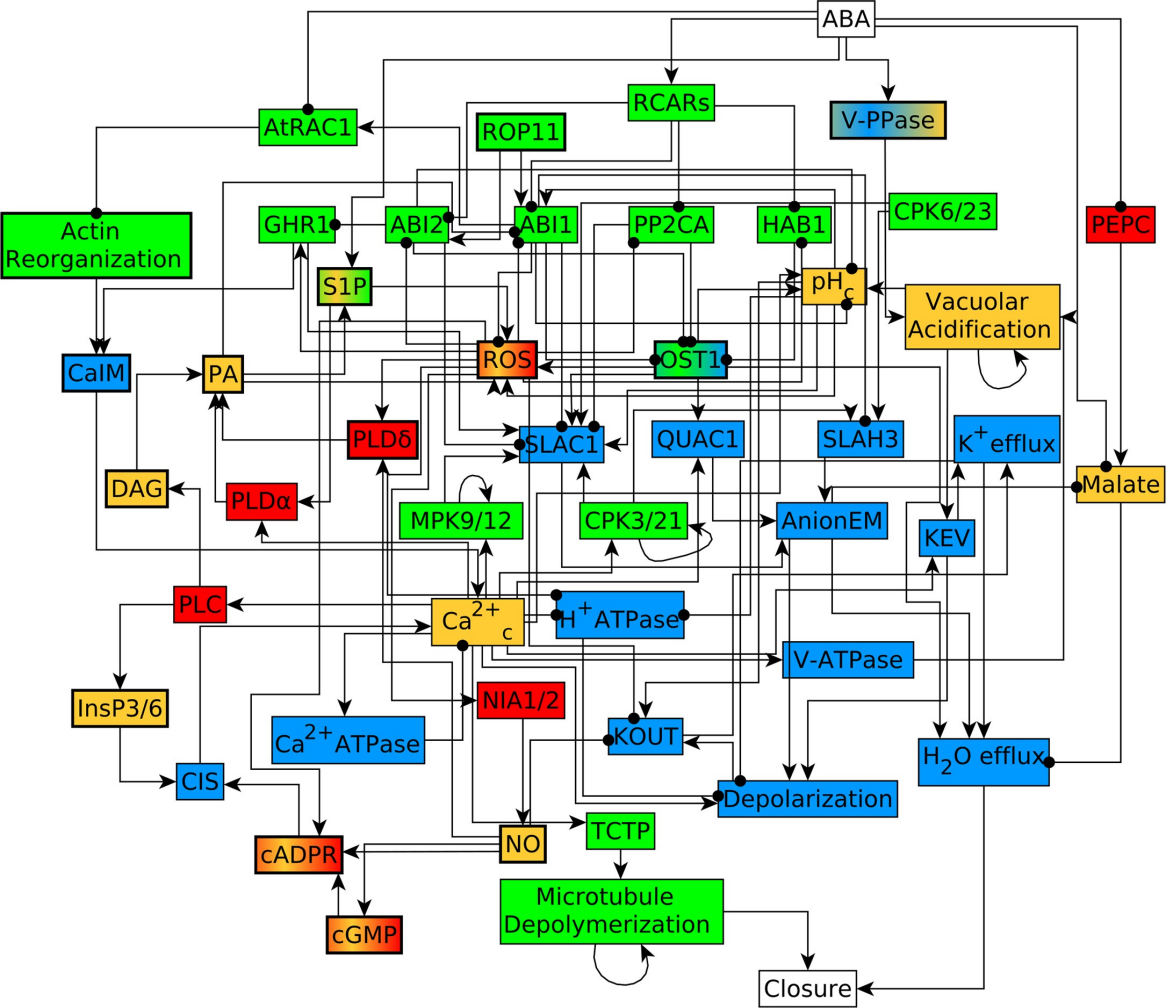
Reduced version of the signal transduction network corresponding to ABA induced closure [4]. Edges ending in an arrowhead denote an activating process or regulation while those ending in filled circles denote an inhibitory regulation. Nodes that are enzymes are shown in red color, signaling proteins in green, membrane-transport related nodes in blue and secondary messengers and small molecules in orange. Symbols with a bold boundary (and in some cases, two colors) mark nodes that merge multiple nodes of the original model; these are listed in detail in Table 1.

The instances of node merging employed in the network reduction are supported by 40 logic equivalence relationships (see Table 1), and by the vast majority of the experimental observations of the effect of knockout or constitutive activation of a node on ABA induced closure (see S2–S5 Tables). This node merging was not done in our original model so as to fully explicate and acknowledge all the biological information in the literature; however, such network reduction simplifies and increases the comprehensibility of the model. Ultimately, network reduction resulted in the equivalent network depicted in Fig 1, with 49 nodes (of which 14 are merged nodes) and 113 edges. The regulatory functions of the reduced model are indicated in S1 Text. For convenience, in Fig 1 and in the following descriptions we use a shorthand notation for the merged nodes; Table 1 indicates the full notation of each merged node.

**Table 1:** Explanation of the merged nodes of the reduced model.

Coded node name	Shorthand notation	Eliminated nodes whose indicated fixed state yields the sustained ON (1) state of the merged node	Eliminated nodes whose indicated fixed state yields the sustained OFF (0) state of the merged node
8-nitro-cGMP→ADPRc→cADPR[NAD^+^]	cADPR	**8-nitro-cGMP = 1**, ADPRc = 1	8-nitro-cGMP = 0, **ADPRc = 0**, NAD^+^ = 0
Actin_Reorganization[ARP_Complex,SCAB1]{b,c}	Actin	None	**ARP_Complex = 0**, **SCAB1 = 0**
CaIM[~ABH1,~ERA1,MRP5,NtSyp121]	CaIM	**ABH1 = 0**, **ERA1 = 0**	None
DAG{c}	DAG	None	**PtdInsP4 = 0**
InsP3[→InsP6]{c}	InsP3/6	InsP3 = 1, InsP6 = 1	**InsP3 = 0**, **PtdInsP4 = 0, InsP6 = 0**
NOGC1→cGMP[GTP]	cGMP	NOGC1 = 1	GTP = 0, NOGC1 = 0
NO[Nitrite]{a}	NO	None	Nitrite = 0, NADPH = 0
OST1[→PIP2;1]	OST1	OST1 = 1	**OST1 = 0**
PA[DAGK,PC]	PA	None	None
PI3P5K→PtdIns(3,5)P2→V-PPase	V-PPase	PI3P5K = 1, PtdIns(3,5)P2 = 1	PI3P5K = 0, PtdIns(3,5)P2 = 0
PLDδ→[GAPC1/2]	PLDδ	None	None
RBOH[RCN1]{b}→ROS{a}	ROS	RBOH = 1	NADPH = 0, **PtdInsP3 = 0**, **RCN1 = 0**, RBOH = 0
ROP11[GEF1/4/10]	ROP11	**GEF1/4/10 = 1**	GEF1/4/10 = 0
SPHK1/2→S1P[~SPP1,Sph] →GPA1[~GCR1]	S1P	**GCR1 = 0**, GPA1 = 1, SPHK1/2 = 1	SPP1 = 1, Sph = 0, GPA1 = 0, SPHK1/2 = 0

The first column lists the coded node names of the nodes that are reduced or merged in the reduction process, following the naming procedures described in the Methods. Explication of the meaning of the coded node names is given in S2–S5 Tables. The second column lists the shorthand notation for the nodes, used for convenience in the figures and tables. The third column lists the settings (in terms of a sustained state of a node that was eliminated during the network reduction process) that yield the sustained ON (1) state of the merged node. Therefore, simulated constitutive activation (i.e., sustained ON state) of the merged node incorporates all of these individual settings. The fourth column lists the settings that yield the sustained OFF state of the merged node; simulated knockout of the merged node incorporates all these settings. The settings that were experimentally studied are indicated in boldface in the last two columns (see S2–S5 Tables for more details and references). The reduced model correctly incorporates the results of all of these experiments.

**Fig 2:**
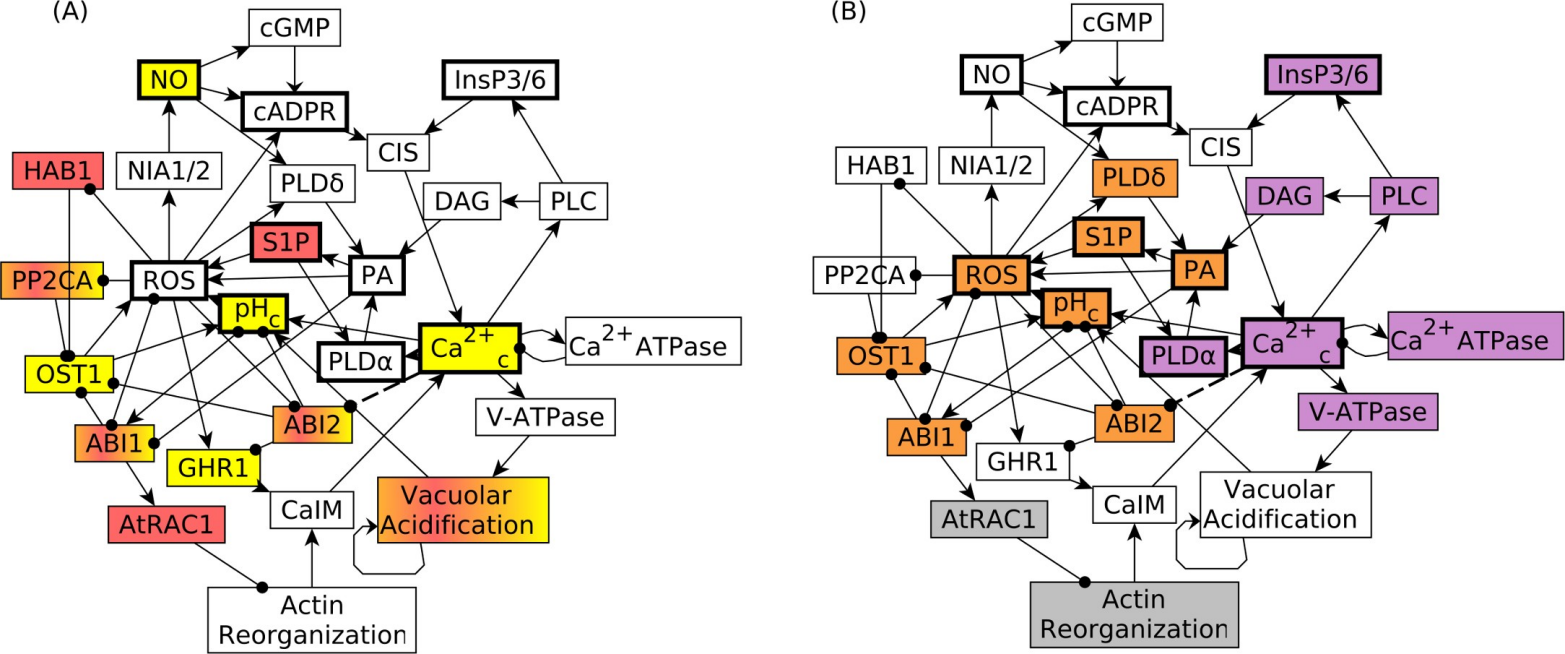
Feedbacks play an important role in the ABA induced closure network and underlie the possibility of closure in response to constitutive activation of an internal node. Both panels show the strongly connected component (SCC) of the network, which contains more than half of the nodes, including 9 internal nodes (shown with thick boundary) whose constitutive activation was observed to lead to closure. The SCC contains both positive and negative feedback loops. **(A)** To illustrate the routes of information propagation, the 7 nodes that are directly affected by the in-component are shown with fuchsia background while the 7 nodes that regulate the out-component are shown with yellow background. Three of the four nodes that belong to both categories (and thus have both backgrounds) are PP2Cs (ABI1, ABI2, PP2CA, HAB1), which inhibit closure and are in turn inhibited by ABA, ROS, or, in the case of ABI1, PA. The model versions studied here reinforce the SCC by additional inhibitory relationship(s) between Ca^2+^_c_ and one or more PP2Cs, here indicated by a dashed line between Ca^2+^_c_ and ABI2. Such inhibition allows the inactivation of PP2Cs even in the absence of ABA. **(B)** The positive feedback loops of the reinforced SCC enable the stabilization of 18 nodes and the achievement of closure upon constitutive activation of certain internal nodes (shown with thick boundary). The nodes of the stable motif associated with closure in the absence of ABA are shown with orange background. The nodes shown with purple background oscillate in the attractors corresponding to closure as a result of the negative feedback loop between Ca^2+^_c_ and Ca^2+^_c_ ATPase. The nodes in white background stabilize in the same state in the attractor corresponding to closure in the presence of ABA (indicated in S7 Table) and in the attractor corresponding to closure in the absence of ABA (shown in S13 Table). The two nodes with grey background have different states in these two attractors. In both panels, edges ending in an arrowhead denote an activating process or regulation while those ending in filled circles denote an inhibitory regulation.

Similar to the complete ABA signaling network, the reduced network has a large feedback-rich center, which in graph theory is called strongly connected component (SCC, see Methods). As shown in Fig 2, the SCC contains 27 nodes and 50 edges, in other words almost half of the network. Four nodes, including ABA, can reach the SCC through paths (i.e. they are in the in-component) and 16 nodes, including Closure, can be reached from the SCC (i.e. they are in the out-component); these nodes are listed in S6 Table. The nodes of the SCC that are directly regulated by nodes in the in-component are shown in fuchsia background in Fig 2A while the nodes that directly regulate nodes in the out-component are shown in yellow background. Four nodes, namely, ABI1, ABI2, PP2CA, and Vacuolar Acidification, are regulated by a node in the in-component and also directly regulate nodes in the out-component. Three of these nodes are PP2Cs, for which both the edges from the in-component and the edges to the out-component are inhibitory. These nodes need to be inactivated either by nodes of the in-component (in response to ABA) or by nodes of the SCC such as ROS, phosphatidic acid (PA), or as proposed here, Ca^2+^_c_, in order to achieve closure.

One of the important organizational features of the ABA-induced stomatal closure network is its stable motifs. Stable motifs are generalized positive feedback loops that maintain a fixed state of their constituent nodes once stabilized [19]. The effects of this stabilization propagate through the network, and channel the dynamics of the system into a subset of its full state space. Ultimately, a stable motif or a combination of stable motifs determine the attractor(s) (long-term dynamic behaviors) of the system. We verified that the reduced model preserved the original stable motifs (compare S1 Fig with Fig 2B–2E of [4]). Multiple stable motifs are associated with ABA-induced stomatal closure (S1A Fig), and a family of related motifs describe the lack of closure in the absence of ABA (S1B and S1D Fig). The reduced model also retained a stable motif associated with closure in the absence of ABA (S1C Fig and orange nodes in Fig 2B), which, consistent with biological reality, does not stabilize in any of the trajectories of the simulated wild type system. A key reason for the lack of realization of the stable motif is that in the absence of ABA the PP2Cs are active in all trajectories.

The reduced model’s attractors (determined as described in the Methods section and summarized in S7 Table) are equivalent to the attractors of the original model, as expected. In the attractor associated with closure in the presence of ABA, 30 nodes stabilize in the ON state, 9 nodes stabilize in the OFF state, and 9 nodes oscillate. The oscillating nodes are driven by the negative feedback loop formed by Ca^2+^_c_ increase and Ca^2+^ ATPase (the node with a function to decrease Ca^2+^_c_). These nodes are shown in purple background in Fig 2B. Indeed, the existence of Ca^2+^_c_ oscillations in ABA-induced stomatal closure is well-established [11,20]. In the simulations, these 9 nodes switch between ON and OFF periods of one to three timesteps, with a variability in duration caused by the stochasticity of the model (see Methods). The average length of the ON and OFF periods of these nodes (see Methods) show clear regularities, as described in S2 Text. Ca^2+^_c_ and Ca^2+^ ATPase have average ON and OFF periods of 1.33 time steps. This is the same value as the average ON/OFF period of the nodes of a two-node negative feedback loop with no outside regulators, consistent with the fact that in the long-term dynamics of the system both positive regulators of Ca^2+^_c_ (CIS, which stands for Ca^2+^ influx from internal stores to the cytosol, and CaIM, which denotes Ca^2+^ influx through the membrane) have stabilized in the ON state. The group of nodes PHOSPHOLIPASE C (PLC), TRANSLATIONALLY CONTROLLED TUMOR PROTEIN (TCTP), QUICKLY ACTIVATING ANION CHANNEL 1 (QUAC1), PHOSPHOLIPASE D α (PLDα) and VACUOLAR PROTON ATPase (V-ATPase), which are all directly regulated by (i.e., at distance 1 from) Ca^2+^_c_, have a longer average ON/OFF period than Ca^2+^_c_. The node that merges inositol 1,4,5 trisphosphate and hexakiphosphate (InsP3/6), as well as diacylglycerol (DAG), both of which are at distance 2 from Ca^2+^_c_, have a longer average ON/OFF period than the nodes directly regulated by Ca^2+^_c_. The increase in the average ON/OFF period with increasing distance from Ca^2+^_c_ reflects the increased chance that the effect of a transient change in Ca^2+^_c_ does not translate into activation of these downstream nodes (see S2 Text). In summary, the attractor corresponding to closure in the presence of ABA features a constant state for 39 nodes and three classes of oscillations for 9 nodes. This attractor signature serves as a comparison for all model versions considered subsequently.

There are multiple attractors associated with lack of closure in the absence of ABA, but in all of them, the same 8 nodes stabilize in the ON state and the same 31 nodes stabilize in the OFF state (see S7 Table). Six nodes stabilize in either the OFF or ON state. These nodes are directly or indirectly affected by positive self-regulation thus if they are activated during the dynamics of the system, they will maintain that activity. Finally, in the absence of ABA, three nodes affected by a negative feedback loop between depolarization and K^+^ efflux through outwardly-rectifying channels (KOUT) either oscillate or are stabilized in the OFF state.

We performed in silico simulations with the original 81-node and reduced 49-node model (see Methods and S1 Text) and determined the percentage of closure in the presence and absence of ABA and in settings where any node (except ABA and Closure) is knocked out or constitutively activated. Compilation of the results for simulated knockout and constitutive activation of each node of the original model in the presence of ABA (S8 Table) supports the validity of network reduction. S8 Table marks the groups of nodes that are merged into a single node in the reduced model. For the majority of groups all interventions in the same group yield the same ABA response. The exceptions consist of tautological interventions such as external supply of an already-abundant molecule (the elimination of which is a minimal loss of information) and a small number of interventions into source nodes that have multiple targets or whose targets have multiple regulators. The reduced model reproduces the results of the original model [4] with 10 minor discrepancies (all involving the speed of reaching 100% closure) out of the 188 possible knockout or constitutive activity settings, see S9, S10 and S11 Tables. A characteristic example of discrepancy between the full and reduced models is the knockout of the nitrate reductases NIA1/2 in the presence of ABA. Knockout of nitrate reductase activity is predicted to lead to a close to wild type response in the reduced model, consistent with experimental evidence [21], while the full model predicted ABA hypersensitivity. Collectively, these results verify that the reduction methods preserve the dynamics of the system.

### Model predictions concerning Ca^2+^_c_ inhibiting one or more PP2Cs

Our original model successfully recapitulated 13 instances of experimentally observed stomatal behavior upon constitutive activation of an internal node in the absence of ABA and did not recapitulate 9 experimental findings of closure [4]. We made two key observations in our previous analysis: (i) the 9 nodes whose constitutive activation can lead to closure according to experiments but not in the model participate in paths that converge to Ca^2+^ release from stores and (ii) even transient inactivation of the PP2C phosphatases is able to resolve 8 discrepancies. We concluded that analysis with the observation that predicted Ca^2+^_c_ inhibition of all four PP2C protein phosphatases would resolve all but one case of discrepancy between experiment and model prediction (see S3 Text for a more detailed description of the argument). As illustrated in the second column of Table 2, because of node merging in the reduction process, the total number of nodes for which there are experimental data on closure in the absence of ABA in the reduced model is 17. The number of cases of agreement with experiments in the absence of ABA in the reduced model is 10 (2 of which are merged nodes) and the number of instances of disagreement with experiment is 7 (5 of which are merged nodes). As shown in the second column of Table 2, in the majority of cases of constitutive activation of a node the percentage of closure was close to the baseline (i.e., close to the case of no constitutive activation); 0% in this case. Only the constitutive activation of ROS yielded a significantly increased percentage of closure.

**Table 2:** Comparison between experimental results and model simulation outcomes upon simulated constitutive activation (CA) of all nodes in the model for which corresponding experimental evidence on constitutive activation in the absence of ABA exists, under different assumptions regarding Ca^2+^_c_ inhibition of PP2Cs.

Category of closure response	Nodes in each category in the reduced model	Nodes in each category when adding a direct inhibitory edge from Ca^2+^_c_ to ABI1	Nodes in each category when adding a direct inhibitory edge from Ca^2+^_c_ to ABI2, to HAB1, to PP2CA, or to all four PP2Cs	Nodes in each category when adding a direct inhibitory edge from PA to ABI2, to HAB1, to PP2CA, or to all four PP2Cs
Close to baseline	**TCTP** [22], **ROP11** [23], **Microtubule Depolymerization** [24], Ca^2+^_c_ [15], InsP3/6 [25], cADPR [26], pH_c_ [27], **PLDα** [28], PA [29], NO [21], S1P [30,31], **AtRAC1** [32], **H**^**+**^ **ATPase** [33], **PP2CA** [34], **ABI1** [12], **ABI2** [12]	**TCTP** [22], **ROP11** [23], **Microtubule Depolymerization** [24], Ca^2+^_c_ [15], InsP3/6 [25], cADPR [26], pH_c_ [27], **PLDα** [28], PA [29], NO [21], S1P [30,31], **AtRAC1** [32], **H**^**+**^ **ATPase** [33], **PP2CA** [34], **ABI1** [12], **ABI2** [12]	**TCTP** [22], **ROP11** [23], **Microtubule Depolymerization** [24]	**TCTP** [22], **ROP11** [23], **Microtubule Depolymerization** [24]
Significantly increased	**ROS** [8,35]	**ROS** [8,35]	**InsP3/6** [25], **cADPR** [26], **ROS** [8,35], **Ca**^**2+**^_**c**_ [15],	**InsP3/6** [25], **cADPR** [26], **ROS** [8,35], **Ca**^**2+**^_**c**_ [15]
Slightly increased	-	-	**S1P** [30,31], **pH**_c_ [27], **PLDα** [28], **PA** [29], **NO** [21]	**S1P** [30,31], **pH**_**c**_ [27]**, PLDα** [28], **PA** [29], **NO** [21]
Decreased	-	-	**PP2CA** [34], **H**^**+**^**ATPase** [33]**, AtRAC1** [32]	**PP2CA** [34], **H**^**+**^**ATPase** [33]**, AtRAC1** [32]
Significantly decreased	-	-	**ABI2** [12]**, ABI1** [12]	**ABI1** [12]**, ABI2** [12]

The first column lists the various response categories (see Methods) and the next four columns indicate nodes that belong to each response category. The merged nodes are underlined. Simulated constitutive activation of the nodes shown in bold font are supported by experimental observations (see Methods) while the others are not; thus, it is evident that agreement is achieved by the assumptions in the fourth and fifth columns. In the third column, we see that when Ca^2+^_c_ constitutively inhibits ABI1, the results are the same as for the reduced model shown in the second column, and equivalent to those reported previously [4]. The fourth column indicates the results when Ca^2+^_c_ directly inhibits ABI2; since the model in [4] as well as our equivalent reduced model already contains an inhibiting path from Ca^2+^_c_ to ABI1, this column represents the case when Ca^2+^_c_ inhibits both ABI1 and ABI2. The same response categorizations are obtained in the model versions where Ca^2+^_c_ directly inhibits HAB1, Ca^2+^_c_ directly inhibits PP2CA, or Ca^2+^_c_ inhibits all four PP2Cs. Furthermore, the same response categorizations are obtained in the cases where the Ca^2+^_c_ inhibition is assumed to be mediated by PA, i.e., in the model versions where PA directly inhibits ABI2, HAB1, PP2CA or all four PP2Cs (fifth column).

**Fig 3:**
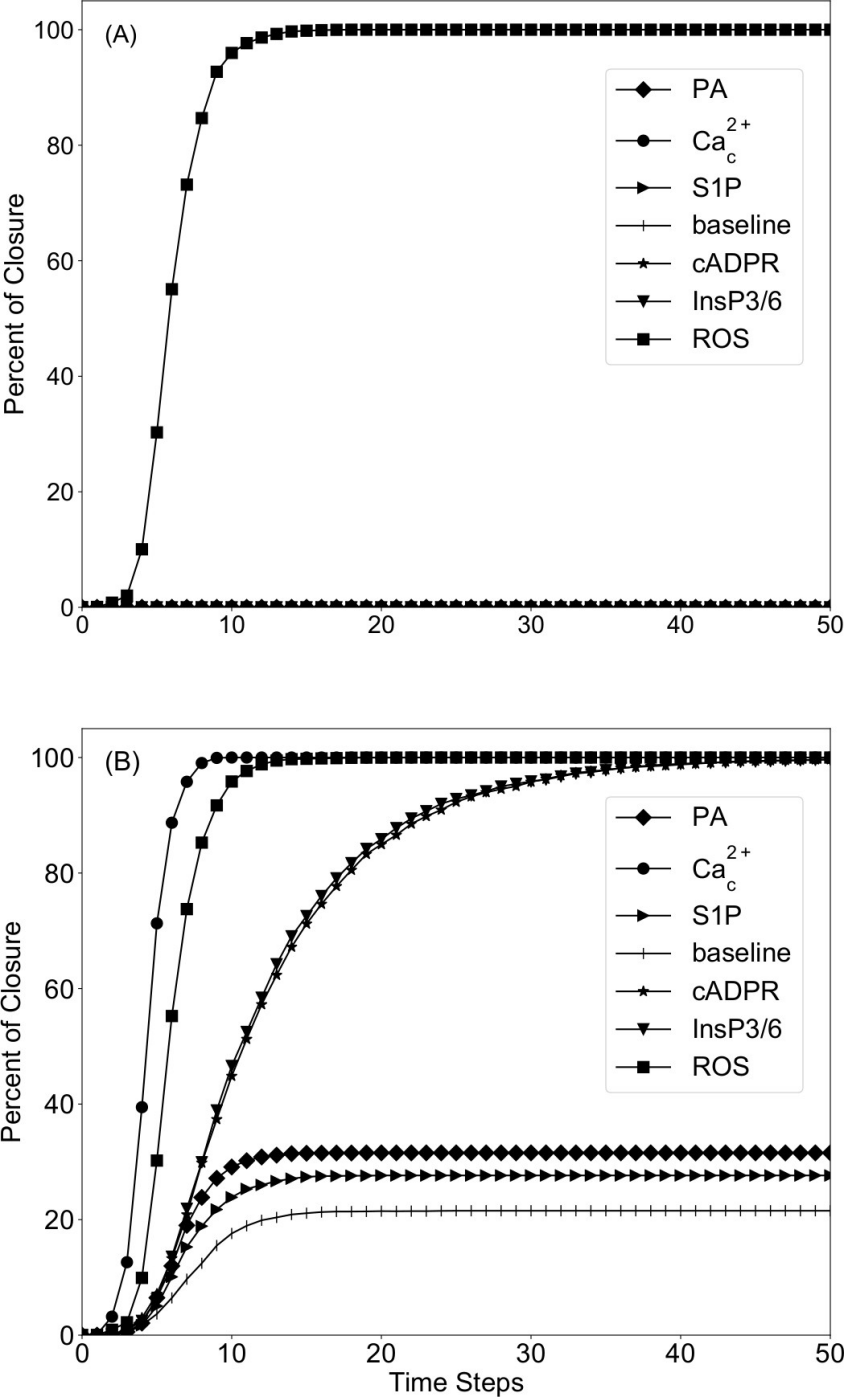
Simulation of constitutive activation of different nodes in the absence of ABA for two assumptions regarding the effect of Ca^2+^c on ABI2. **Panel (A) indicates the original assumption (i.e., no additional edge) and panel (B) represents the assumption that Ca**^**2+**^_**c**_ **inhibits ABI2**. The y-axis denotes the percentage of closure across 4500 simulations when ABA is off. The x-axis denotes the time steps (rounds of node update) of the simulation. **(A)** In the absence of inhibition of PP2Cs by Ca^2+^_c_, only the constitutive activation (CA) of ROS (squares) yields a nonzero percentage of closure in the absence of ABA. All the other situations lead to 0% closure. **(B)** When assuming Ca^2+^_c_ inhibition of ABI2, there is a baseline percentage of closure of 21.5% (see Methods). The constitutive activation of Ca^2+^_c_ (filled circles), ROS (squares), InsP3/6 (downward triangles) or of cADPR (stars) yields 100% closure, but with different timing. The constitutive activation of PA or S1P gives a slightly increased response compared to baseline.

In this report, we newly study the relationship between Ca^2+^_c_ and the four PP2C protein phosphatases in detail. We separately analyze various scenarios of Ca^2+^_c_ inhibiting a single PP2C (which would be the most parsimonious assumption, see S3 Text) or multiple ones and compare model simulations with the available experimental results (Tables 2 and S12). We first performed four simulations, adding an inhibitory edge from Ca^2+^_c_ to each one of the PP2Cs (ABI1, ABI2, HAB1 and PP2CA), with no other changes. Simulations of *direct* Ca^2+^_c_ inhibition of ABI1 yielded results identical to those obtained in the absence of any additional inhibitory edge from Ca^2+^_c_ (third column of Table 2). We uncovered that this is because an *indirect* inhibitory effect between Ca^2+^_c_ and ABI1 was already incorporated by multiple pathways in the model, such that the newly posited edge, in the case of ABI1, is completely redundant. One such indirect pathway is Ca^2+^_c_ → PLC → DAG → PA–● ABI1; this pathway is discussed in more detail in the next section. In contrast, when Ca^2+^_c_ is assumed to inhibit any one of ABI2, HAB1 or PP2CA we observed that the constitutive activation (CA) of Ca^2+^_c_ can lead to complete stomatal closure (fourth column of Table 2). This computational outcome agrees with multiple observations that experimental elevation of Ca^2+^_c_ suffices to elicit stomatal closure [11,13,15]. Moreover, addition of an inhibitory edge from Ca^2+^_c_ to any one of ABI2, HAB1, or PP2CA also successfully captures all 9 cases of experimentally observed closure due to constitutive activation of a node, including pH_c_, nitric oxide (NO), cyclic ADP-ribose (cADPR), PA, and InsP3 [21,25,27,29,36]. Fig 3 compares the percentage of closure for constitutive activation of the node that merges sphingosine, sphingosine kinases and sphingosine-1-phosphate (S1P), the node that merges 8-nitro- cyclic GMP, ADP ribosyl cyclase and cADPR (denoted cADPR), ROS (which is a merged node representing reactive oxygen species and the enzyme RBOH), PA, Ca^2+^_c_ in the reduced model (panel A) and the model version that assumes that Ca^2+^_c_ inhibits ABI2 (panel B). Constitutive activation of ROS yields 100% closure in both model versions. The model version that assumes that Ca^2+^_c_ inhibits ABI2 outperforms the original reduced model by yielding an increased closure percentage when S1P or PA are constitutively activated, and 100% closure for constitutive activation of cADPR, InsP3/6, Ca^2+^_c_. All these results are consistent with experimental knowledge [15,21,25,27,29–31,36]. An explanation of the two categories of increase in the percentage of closure is provided in a later section.

The added inhibition of ABI2 also yields an increase in the baseline percentage of closure. To understand this result, we determined the attractors of the unperturbed system (without any node knockout or constitutive activation). These attractors are the same in the model versions wherein Ca^2+^_c_ directly inhibits ABI2 or HAB1 or PP2C, or in which Ca^2+^_c_ directly inhibits more than one of these PP2Cs. All these model versions have the same attractor corresponding to closure in the presence of ABA and the same set of attractors corresponding to lack of closure in the absence of ABA as the original reduced model (compare S7–S13 Tables). However, they also have an additional attractor, which describes closure in the absence of ABA. This attractor agrees with the attractor describing closure in the presence of ABA in the state of 43 nodes (including 9 oscillating nodes) and differs in 5 nodes (S13 Table and Fig 2B). This attractor is reachable due to the possibility of activating the stable motif corresponding to closure in the absence of ABA (Fig 2B); thus, trajectories that reach this attractor yield a nonzero baseline percentage of closure. In contrast, this attractor is not reachable in the reduced model, nor in the reduced model version wherein we add a direct inhibitory edge from Ca^2+^_c_ to ABI1, thus the baseline percentage of closure is 0 in these models. The mechanisms of activating the stable motif underlying closure in the absence of ABA are explained in detail in the next section.

The response categories of the constitutive activation simulation results are the same whether any two or more than two PP2Cs are inhibited by Ca^2+^_c_. The baseline probabilities of closure are slightly different in each model version, see S12 Table. A main contributor to the similarity of the model outcomes is the symmetry of the regulatory function of OST1, which incorporates the assumption that the inactivity of any two PP2Cs is sufficient to activate OST1 (see S2 Text of [4]). As detailed in the next section and in S4 Text, OST1 is a part of a stable motif that drives the system to closure, thus the equivalence of two settings in activating OST1 translates to their being equally effective in activating the stable motif and yielding closure.

While the motivation underlying our hypothesized inhibition of the PP2Cs is to accurately predict experimental results of closure in the absence of ABA upon constitutive activation of key internal nodes, it is important to check that the thus-augmented model maintains agreement with experimental results in the presence of ABA. As summarized in S14 Table, the assumed inhibition introduces a single discrepancy: the knockout of the node RCARs yields a milder defect in the model than in experimental observations (see the caption of S14 Table for a discussion of this discrepancy).

### Theoretical analysis of the stable motif associated with closure in the absence of ABA

**Fig 4:**
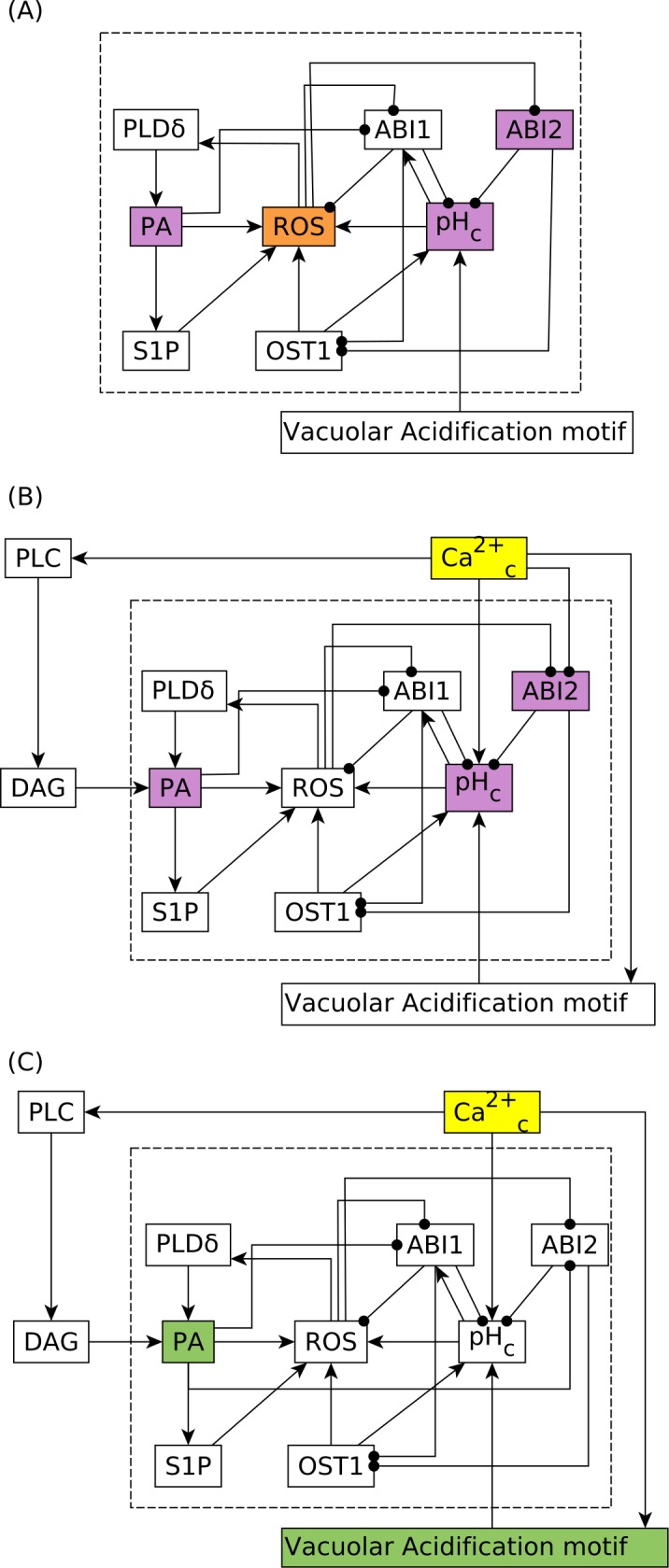
Stable motif corresponding to the attractor that has Closure = ON when ABA = OFF. The stable motif states are as follows: PLDδ = ON, PA = ON, S1P = ON, ROS = ON, OST1 = ON, pH_c_ = ON, ABI1 = OFF, ABI2 = OFF. The stabilization of the Vacuolar Acidification motif (and the corresponding ON state of Vacuolar Acidification) is a required precursor of this stable motif in all versions of the model. **(A)** The stable motif in the absence of any additional inhibitory edge to any of the PP2Cs. ROS (shown in orange color) is a single-node driver of this motif, i.e., if ROS has a sustained ON state, it can stabilize the entire motif, hence eventually leading the network to the Closure = ON attractor. PA, pH_c_ and ABI2 (purple nodes) form a three-node driver set, that is, if PA is ON, pH_c_ is ON and ABI2 is OFF, then the entire motif stabilizes, leading to closure. Note that not every combination of three nodes belonging to the motif shown in (A) can stabilize the entire motif; see S5 Text for more information. **(B)** The corresponding stable motif in the model version where Ca^2+^_c_ is assumed to inhibit ABI2. In this case, if Ca^2+^_c_ is set to ON, it stabilizes PA = ON, pH_c_ = ON, and ABI2 = OFF (through its edges incident on nodes of the motif), which forms a three-node driver, stabilizing the entire motif, hence leading to closure. **(C)** The stable motif in the model version where PA is assumed to inhibit ABI2. PA and Vacuolar Acidification (shown in green color) make up a two-node driver set of the motif, as PA inhibits ABI1 and ABI2, which together with Vacuolar Acidification activate pH_c_, making this combination equivalent to the PA = ON, pH_c_ = ON, ABI2 = OFF three-node driver of the motif. The Ca^2+^_c_→ PLC → DAG → PA path is sufficient for the activation of PA, thus Ca^2+^_c_ remains an external driver of the stable motif associated with closure.

For a deeper understanding of how the inhibition of PP2Cs by Ca^2+^_c_ can yield ABA-independent closure, we used causal logic analysis [16] to determine the drivers of the stable motif associated with closure in the absence of ABA (see Methods). Stabilization of a stable motif (or successive stabilization of a group of stable motifs) can drive an entire network to a particular attractor. These motifs can be identified by finding strongly connected components (SCCs) in an expanded network that expresses the regulatory function of each node [19]. Two different stable motifs were identified in our original model [4] in the absence of ABA (and reproduced by the reduced model, see S1 Fig), one of which is associated with closure in the absence of ABA. This stable motif, which can be stabilized only after the stabilization of the Vacuolar Acidification motif, is shown in reduced form in Fig 4A. The stable motif primarily contains the nodes ROS, PA, PLDδ (Phospholipase D δ), pH_c_, OST1, S1P, and the PP2Cs ABI1 and ABI2. We found that the node ROS is a driver node of this motif, which means that if ROS is set to the ON state, the entire motif stabilizes and eventually leads to the closure attractor of the network. There also exist three-node combinations whose stabilization can lead to the stabilization of the motif. For example, PLDδ, pH_c_ and ABI2 make up a three-node driver set, that is, if PLDδ and pH_c_ are fixed to ON and ABI2 is fixed to OFF, then the entire motif stabilizes, leading to closure. In the reduced model, ROS is the only node in the network whose constitutive activation can yield the stabilization of the motif and closure in the absence of ABA, consistent with the results in Fig 3A and S11 Table.

We next studied the model version wherein Ca^2+^_c_ directly inhibits ABI2. In this version, Ca^2+^_c_ becomes an external driver node of the motif associated with closure in the absence of ABA (Fig 4B). Moreover, as described in S4 Text, we find that a Ca^2+^_c_ pulse (sustained ON state) of four or more time steps, or multiple repeated Ca^2+^_c_ spikes (where one spike is equated to an ON state for a single time step) separated by one or two time steps, also yield the consistent activation of the closure-inducing stable motif. These conclusions are also valid in the model versions where Ca^2+^_c_ directly inhibits HAB1 or PP2CA or multiple PP2Cs (see S4 Text). These results are consistent with experimental observations that Ca^2+^_c_ oscillations induced by membrane hyperpolarization/depolarization cycles induce closure in the absence of ABA [11]. These conclusions on the effect of Ca^2+^_c_ spikes however do not hold true for the original reduced model, illustrating the explanatory power of the added inhibition.

We also found that when InsP3/6 or cADPR (two of the nodes that are upstream of Ca^2+^_c_ and whose constitutive activation, computationally or experimentally, leads to closure) are in their ON state, this eventually leads to Ca^2+^_c_ exhibiting a sustained oscillation pattern. Our model predicts that the constitutive activation of InsP3/6 or cADPR in the absence of ABA leads to sustained Ca^2+^ influx from internal stores to the cytosol (CIS), in agreement with experiments [25,36]. The model also predicts that Ca^2+^_c_ then follows the oscillatory pattern described in S2 Text, which yields the stomatal closure attractor in 100% of our simulations. In total there are eight nodes whose constitutive activation can elicit cytosolic Ca^2+^ oscillations, which then lead to the stabilization of the stable motif associated with closure, and ultimately yield stomatal closure: Actin reorganization, CaIM, CIS, cADPR, GUARD CELL HYDROGEN PEROXIDE RESISTANT 1 (GHR1), InsP3/6, PLC, ROS. Indeed, these eight nodes and Ca^2+^_c_ populate the category “significantly increased closure” in the absence of ABA (see S12 Table). These results support the view that Ca^2+^_c_ is a key driver of the process of stomatal closure in the absence of ABA [11,13,15].

### Experimental assessment of PP2C inhibition by Ca^2+^

Our simulation results and their agreement with reported experimental results gave us a strong basis for predicting that Ca^2+^_c_ inhibits the PP2Cs. This prediction was congruent with the observation that ABI1 has a candidate EF-hand Ca^2+^-binding motif [37,38]. Indeed, the presence of this motif previously inspired three independent studies of potential direct Ca^2+^ regulation of ABI1, all of which assayed the phosphatase activity of recombinant ABI1 in vitro. Giradaut and co-workers observed essentially the same extent of Ca^2+^ inhibition of phosphatase activity for recombinant full-length ABI1 and truncated ABI1 interrupted in the EF hand motif [39]; in both instances, inhibition occurred in the mM Ca^2+^ range, leading to the conclusion that Ca^2+^ was inhibiting the catalytic domain, rather than binding the EF-hand. Grill and colleagues similarly observed Ca^2+^ inhibition of recombinant ABI1 only at unphysiologically high Ca^2+^concentrations, with a half-inhibitory concentration of 8 mM; they suggested that Ca^2+^ may be hindering the Mg^2+^ binding required for phosphatase activity [40]. Finally, Sheen mutated the aspartate that is required for Ca^2+^ binding in the candidate EF-hand and observed no impact on phosphatase activity [41]. In summary, these studies concluded that no direct Ca^2+^ regulation of ABI1 could be observed within the physiological range prevailing in the cytosol (approximately 100 nM to low μM concentrations). The fact that these experiments were conducted in vitro under carefully controlled conditions provides no support for our hypothesis of direct Ca^2+^_c_ inhibition of PP2Cs. We therefore considered whether the regulation of PP2Cs by Ca^2+^_c_ could be via an indirect mechanism. Indeed, by the causal logic theory [16], almost the same system dynamics, and the same attractors, could be obtained under the assumption that the PP2C inhibition by Ca^2+^_c_ occurs indirectly, via a pathway that has the same logical implication as a direct edge from Ca^2+^_c_ to the PP2Cs.

**Fig 5:**
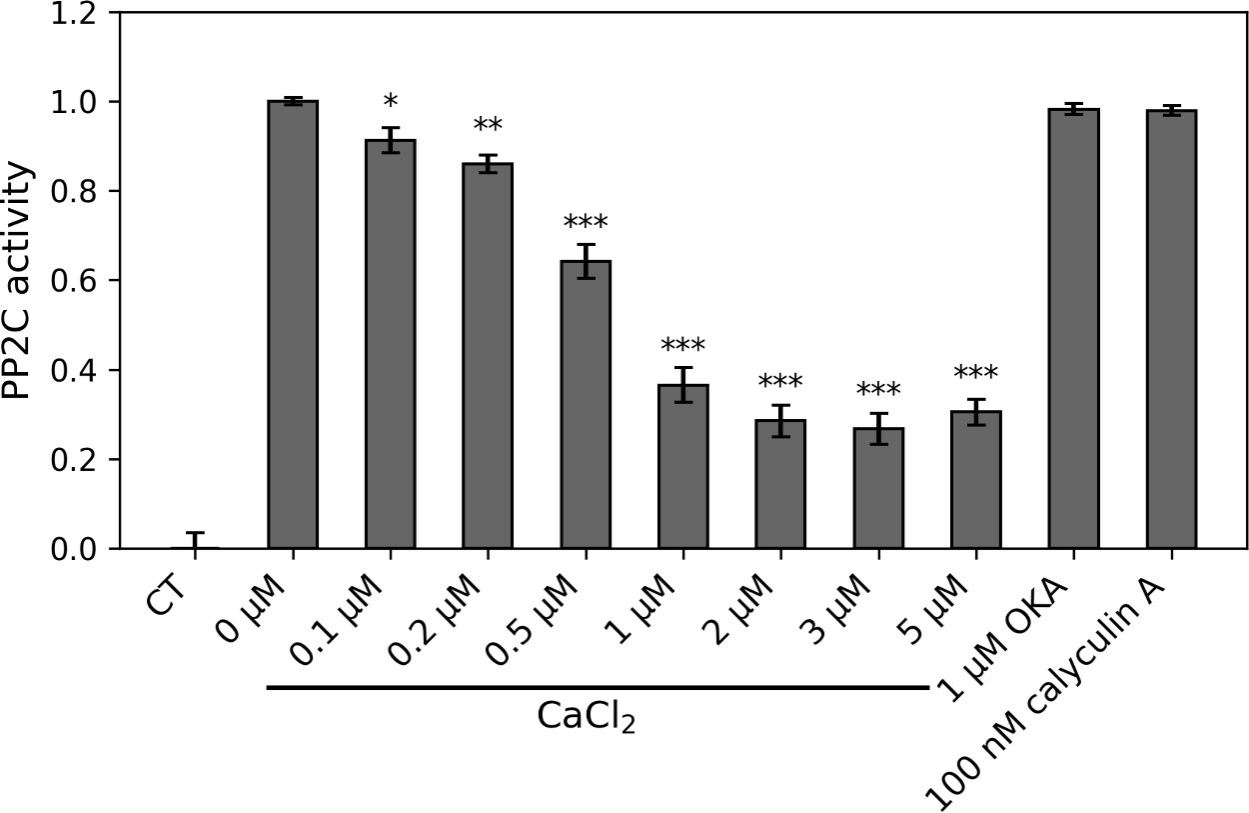
PP2C activity in Arabidopsis mesophyll cell lysates is inhibited by physiological Ca^2+^ concentrations. PP2C activity assays were carried out in the absence or presence of 0.1 to 5 μM CaCl_2_. Either 1 μM Okadaic acid (OKA) or 100 nM Calyculin A, both of which are potent PP1 and PP2A chemical inhibitors, was added to the reaction to confirm the specificity of PP2C activity based on ^32^P-casein dephosphorylation. PP2C activity without CaCl_2_ inhibition is presented as 1.0 (100% activity). CT: the control reaction without lysates. The error bars represent standard deviation based on n = 3 biological replicates. * P < 0.05, ** P < 0.01, *** P < 0.001 (One-way ANOVA with Dunnett’s multiple comparisons test).

Accordingly, we decided to assay whether physiological levels of Ca^2+^ in the presence of cellular (protoplast) lysates without the influence of high apoplastic Ca^2+^ would inhibit PP2C activity. As shown in Fig 5, we did indeed observe inhibition of PP2C activity in the lysates by low μM levels of Ca^2+^, corresponding to levels that prevail in the cytosol. These results, in conjunction with the prior in vitro studies [39–41], lend support to the idea that inhibition of PP2C activity by Ca^2+^_c_ does occur in vivo, but that the mechanism of inhibition is indirect.

### PP2C inhibition mediated by PA gives similar results

**Fig 6:**
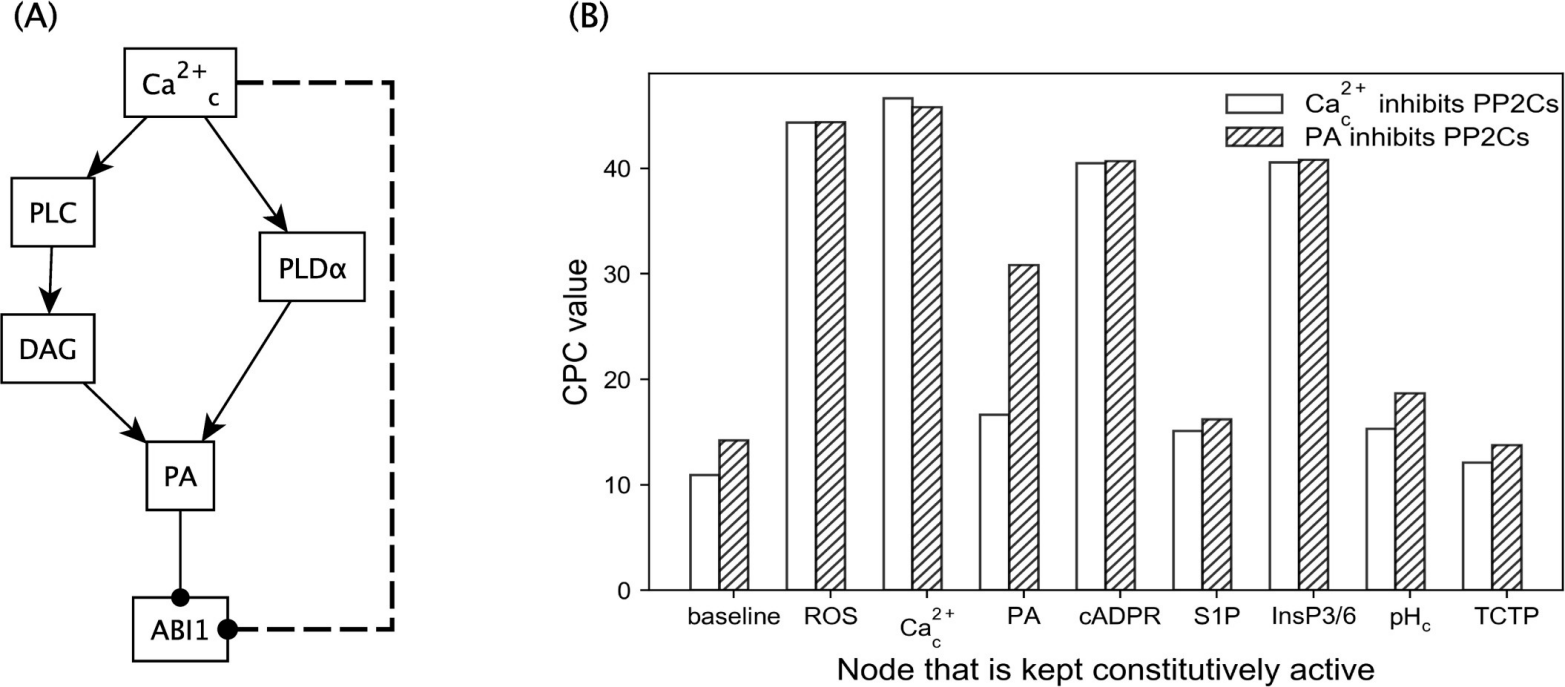
Model outcomes for Ca^2+^c inhibition of PP2Cs via PA. **(A)** Different pathways by which Ca^2+^_c_ contributes to PA production are shown. Edges ending in a filled circle denote inhibitory edges. Given the paths from Ca^2+^_c_ to PA, the inhibition of ABI1 by PA means that Ca^2+^_c_ activation indirectly inhibits ABI1. The same argument also applies to ABI2, HAB1 and PP2CA. In terms of network structure and Boolean logic, indirect inhibition of the PP2Cs by Ca^2+^_c_ has the same effect as direct inhibition (dashed line from Ca^2+^_c_ to ABI1), hence we expect to obtain similar simulation results. **(B)** Simulation results of the extent of closure upon node constitutive activation when either PA inhibits the PP2Cs or Ca^2+^_c_ directly inhibits the PP2Cs. The y-axis denotes the cumulative percentage of closure (CPC) over 50 timesteps (see Methods) in case of constitutive activation (CA) of different nodes. Open bars refer to the case where Ca^2+^_c_ inhibits the PP2Cs directly while hatched bars refer to the case where PA inhibits the PP2Cs.

We then turned to our model to elucidate possible pathways for indirect Ca^2+^_c_ inhibition of PP2Cs. We already know about one such indirect pathway: Ca^2+^_c_ → PA–● ABI1, wherein Ca^2+^_c_ contributes to PA production by upregulating PLDα and PLC (Fig 6A) [42–46], and PA is known to directly bind to and inhibit one of the PP2Cs (ABI1) [47]. Thus, we next considered the alternative hypothesis that PA binds to and inhibits additional PP2Cs (see S3 Text) and evaluated this hypothesis by simulations and stable motif analysis. We found that this assumption led to almost identical results as assuming that Ca^2+^_c_ directly inhibits the PP2Cs. The attractors of the model in the presence or absence of ABA in the case of inhibition of the PP2Cs by PA are identical to those in case of inhibition of the PP2Cs by Ca^2+^_c_, shown in S13 Table. The stable motif corresponding to closure in the absence of ABA is identical except for the replacement of the Ca^2+^c−● ABI2 edge with a PA–● ABI2 edge (Fig 4C). ROS and Ca^2+^_c_ remain internal and external drivers, respectively, of this stable motif. The only difference is that PA becomes an internal driver of the stable motif if Vacuolar acidification is already stabilized at ON.

Moreover, we found the same categorization of the constitutive activation of nodes as in the case where Ca^2+^_c_ directly inhibits the PP2Cs (fifth column of Tables 2 and S14). This means that the two model versions are equally supported by experimental results. As a further illustration of this point, Fig 6B compares the cumulative percentage of closure for the baseline and for constitutive activation of multiple nodes in two versions of the model: the version in which Ca^2+^_c_ directly inhibits the PP2Cs (white bars) and the version in which PA inhibits the PP2Cs (hatched bars). As expected, constitutive activation of Ca^2+^_c_ (open and hatched bars labelled “Ca^2+^_c_” in Fig 6B) has the same effect in both versions of the model. This is because constitutive activation of Ca^2+^_c_ yields constitutive activation of PA. The effect of constitutive activation of multiple other nodes is very similar as well. Constitutive activation of PA yields a stronger effect in the model version where PA inhibits the PP2Cs. This is because constitutive activation of PA does not imply constitutive activation (sustained high concentration) of Ca^2+^_c_, nor of CIS or CaIM. Thus, constitutive activation of PA yields a lower probability of activating the stable motif associated with closure, and thus a lower percentage of closure, in the model version where Ca^2+^_c_ directly inhibits the PP2Cs than in the model version where PA directly inhibits the PP2Cs.

In summary, our modeling outcomes predict that the known inhibition of ABI1 by PA, plus putative inhibition of one or more of the other three PP2Cs by PA, would suffice to equal the outcome of positing direct inhibition of the same PP2Cs by Ca^2+^_c_ in terms of system dynamics. Calcium-induced PLC activation, which, according to our model, is upstream of PA production, occurs in both guard cells and mesophyll cells [46], thus the Ca^2+^_c_ → PA–● PP2C mechanism is consistent with, and a likely contributor to our experiments in cellular lysates. For ABI1, lipid binding experiments with truncated ABI1 constructs have demonstrated that PA binding occurs within the first 104 amino acids of the N-terminus, with the region of residues 67 to 97 being of particular interest given sequence similarity with known PA-binding regions identified in mammalian systems [47]. We therefore analyzed the similarity of ABI2, HAB1, and PP2CA to this region in ABI1. Of these, ABI2 has the greatest similarity to ABI1 in the first 104 amino acids (36% identity and 47% similarity based on pair-wise alignment), including in the domain from residues 67 to 97 (33% identity and 83% similarity) making ABI2 the most likely additional candidate for inhibition by PA.

### The model predicts an interplay between the stable motif and Ca^2+^_c_ oscillations

As described earlier, regularly or irregularly repeated Ca^2+^_c_ spikes yield the consistent activation of the closure-inducing stable motif, which then leads to closure, in all model versions where Ca^2+^_c_ inhibits (directly or indirectly) at least one PP2C other than ABI1. In all model versions including the original model [4], ABA leads to sustained Ca^2+^ influx through the membrane through the ABA–● AtRAC1 –● Actin reorganization → CaIM path [32,48] and also contributes to Ca^2+^_c_ induced Ca^2+^ release from internal stores [49]; thus in the presence of ABA, Ca^2+^_c_ exhibits sustained oscillations (see S2 Text), which then drive closure [20]. We next sought to determine whether it is possible that such closure-driving Ca^2+^_c_ patterns can be generated in the absence of ABA.

**Fig 7:**
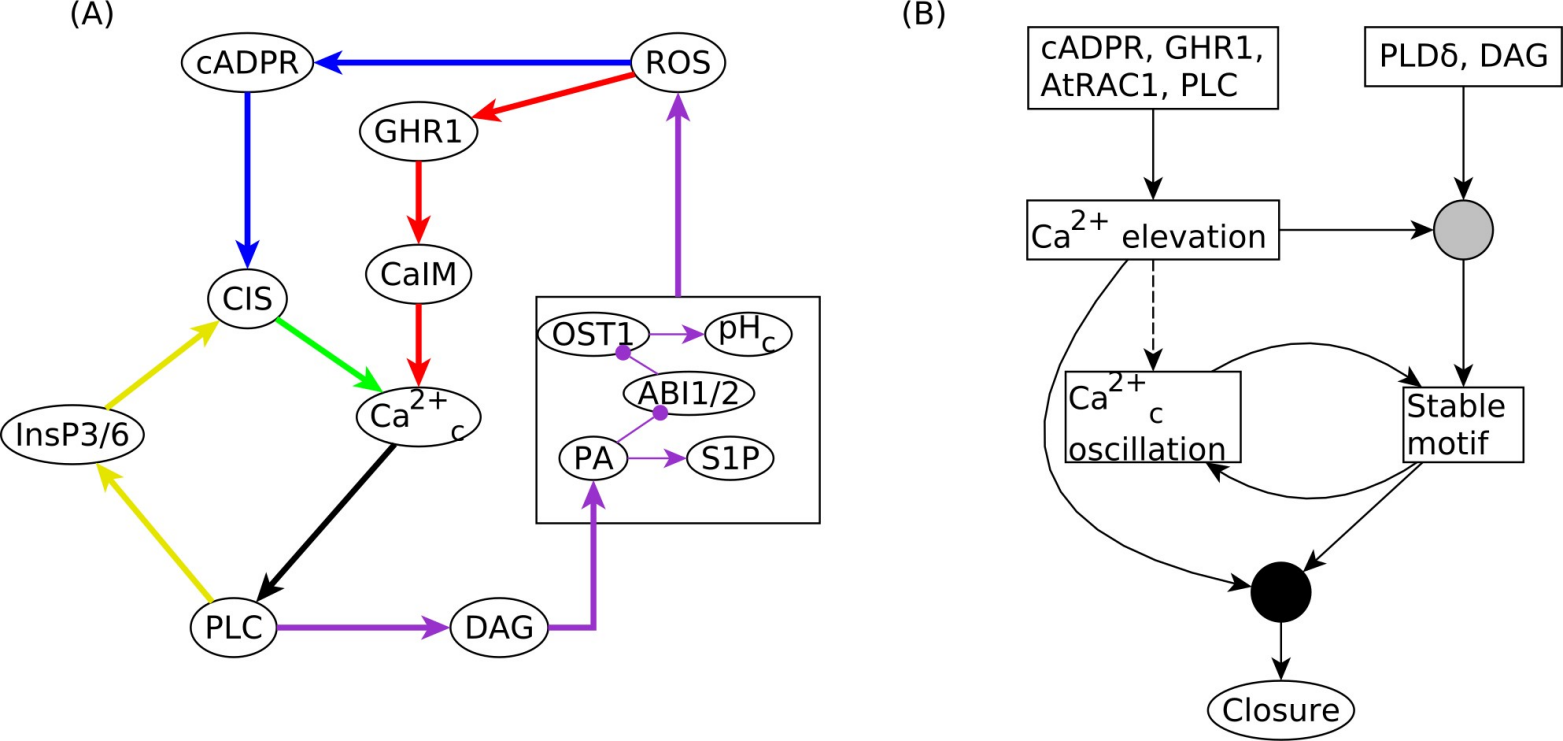
Multiple feedback loops mediate the role of Ca^2+^c in stomatal closure in the absence of ABA in the model version where PA inhibits ABI2. **(A)** Cytosolic Ca^2+^ is affected by three overlapping positive feedback loops. We highlight each feedback loop with a separate color and we separate the edges that participate in multiple feedback loops into as many copies as loops in which they participate. The shortest feedback loop (yellow), consists of the nodes Ca^2+^_c_, PLC, InsP3/6, and CIS. The two longer feedback loops (red or blue) share ROS, which in turn depends on the group of nodes shown inside the box on the right side of the panel. The edges among the nodes inside the box illustrate how PA plays an important role in regulating all of these nodes. The blue feedback loop is mediated by PLC, DAG, the PA-ABI1/2-OST1-pH_c_-S1P group of nodes, ROS, cADPR, and CIS, and overlaps the yellow feedback loop in the CIS→ Ca^2+^_c_ edge. The red feedback loop consists of Ca^2+^_c_, PLC, DAG, the PA-ABI1/2-OST1-pH_c_-S1P group of nodes, ROS, GHR1, and CaIM. The edge from Ca^2+^_c_ to PLC participates in all three feedback loops. **(B)** The logic backbone of the network that underlies stomatal closure in the absence of ABA. Sustained Ca^2+^_c_ oscillations lead to stabilization of the stable motif associated with closure (see S4 Text). In turn, stabilization of that motif due to one of its internal driver sets mediates the red and blue feedback loops in panel A and leads to sustained Ca^2+^_c_ oscillations. Initial activity of PLDδ or DAG can activate PA, which, when combined with Ca^2+^_c_ elevation, can lead to the stabilization of the motif. This is represented by a composite node (grey circle) for which Ca^2+^_c_ elevation for at least one time step and initial activity of PLDδ or DAG are necessary; this composite node is sufficient for stabilization of the stable motif. Initial activity of cADPR, GHR1, or PLC, or inactivity of AtRAC1 can induce Ca^2+^_c_ elevation. Due to the stochasticity incorporated by various possible update orders, Ca^2+^_c_ elevation has a low but non-zero probability (around 0.2) of activating the Ca^2+^_c_ → PLC → InsP3/6 → CIS loop, leading to Ca^2+^_c_ oscillations. This is represented by the dashed edge from Ca^2+^ elevation to Ca^2+^_c_ oscillation. Stomatal closure necessitates the activation of the stable motif and at least a transient Ca^2+^_c_ elevation (whose effect is sustained through the self-regulatory stable motifs MPK9/12, CPK3/21, Vacuolar acidification, and Microtubule depolymerization). This necessary requirement is depicted by a composite node (black circle) for which Ca^2+^_c_ elevation and stable motifs are each necessary, while the composite node itself is sufficient for closure.

Through simulations and causal logic analysis, we find that in the absence of ABA, a sufficient condition for Ca^2+^_c_ elevation is an initial condition wherein at least one of cADPR, GHR1, AtRAC1 (small GTPase RAC1), PLC (in the model version where Ca^2+^_c_ inhibits ABI2) or at least one of cADPR, GHR1, AtRAC1, PLC, PLDδ, DAG (in the model version where PA inhibits ABI2) is initiated in their state corresponding to stomatal closure (see S7 Text). Notably, AtRAC1 is inactivated during stomatal closure, while all the other nodes are activated (see S7 and S13 Tables). The conclusion that initial inactivity of AtRAC1 contributes to Ca^2+^_c_ increase and to stomatal closure is supported by the experimental observation that a loss of function *AtRAC1* mutant induced actin reorganization and stomatal closure [32]. Our analysis indicates that a necessary condition for repeated Ca^2+^_c_ elevation is the sustained activation of at least one of the three positive feedback loops incident on Ca^2+^_c_. As shown in Fig 7A, these feedback loops are Ca^2+^_c_ → PLC → InsP3 → CIS, Ca^2+^_c_ → PLC → DAG → PA → ROS → cADPR → CIS, and Ca^2+^_c_ → PLC → DAG → PA → ROS → GHR1 → CaIM → Ca^2+^_c_. For most of the edges participating in these feedback loops the activity of the source of the edge is sufficient to maintain the activity of the target (e.g. the activity of PLC is sufficient for the production of DAG). The PA→ ROS edge is an exception; the production of ROS has multiple other conditions, which are simultaneously satisfied only when the stable motif associated with closure has stabilized (see Fig 7A and S7 Text). This edge, and thus the stable motif, participates in two of the three Ca^2+^_c_ feedback loops.

PLC participates in all three Ca^2+^_c_ positive feedback loops, thus in the absence of ABA its simulated knockout leads to the stabilization of Ca^2+^_c_ in the OFF state. This prediction is supported by the experimental observation that pharmacological inhibition of PLC abolished oscillations in Ca^2+^_c_ induced by pathogenic elicitors [50,51]. We find that PLC and DAG are also important mediators of the effect of Ca^2+^_c_ on the stable motif associated with closure: the closure-inducing effect of an externally imposed Ca^2+^_c_ pulse or repeated Ca^2+^ spikes are dependent on the Ca^2+^_c_ → PLC → DAG → PA pathway in both model versions. If this pathway is disrupted, the percentage of closure in the model reduces to its baseline value (obtained in the absence of any signal or imposed Ca^2+^_c_ pattern). This model prediction is supported by the experimental observations that pharmacological inhibition of PLC impaired ABA-induced Ca^2+^_c_ oscillations and abolished ABA-induced stomatal closure [20].

Our analysis indicates that there exists a mutual reinforcement between the Ca^2+^_c_ patterns and the stable motif associated with closure in the absence of ABA, as summarized in Fig 7B. Sustained Ca^2+^_c_ oscillations are sufficient to stabilize the stable motif (as described in S4 and S6 Texts). In turn, the stable motif mediates two positive feedback loops centered on Ca^2+^_c_, leading to sustained Ca^2+^_c_ oscillations (as described above and in S7 Text). Stomatal closure necessitates the activation of the stable motif and at least a transient Ca^2+^_c_ elevation, whose effect is then sustained through the self-regulatory stable motifs of the nodes MPK9/12 (Mitogen activated Protein Kinases 9 & 12) and CPK3/21 (CALCIUM DEPENDENT PROTEIN KINASES 3 & 21), Vacuolar acidification and Microtubule depolymerization. Thus, according to the logic backbone of the network corresponding to stomatal closure in the absence of ABA, sustained Ca^2+^_c_ oscillations are sufficient, but not necessary, to induce closure. This is consistent with the observation of closure induced by induced Ca^2+^_c_ oscillations [11], and with both the Ca^2+^_c_ dependent and Ca^2+^_c_ independent aspects of ABA response [52–54].

## Discussion

Here we evaluated our previously posited hypothesis that Ca^2+^_c_ inhibits multiple proteins in the PP2C family [4]. We first derived an accessible reduced model that maintained the properties of our original model. We then found that the reduced model augmented with the assumption that Ca^2+^_c_ inhibits one or more additional PP2C proteins agrees with all the observations that were previously discrepancies. Our combined computational and experimental analyses have led us to a refined hypothesis, in which Ca^2+^_c_ elevation results in production of PA, which binds and inhibits ABI1 and a second PP2C, plausibly ABI2, thus engendering strong Ca^2+^_c_-induced stomatal closure in the absence of ABA. Comparing the performance of the two model versions in settings of constitutive activation of a node, we find that in each setting the model wherein PA is inhibiting ABI2 yields a larger or equal percentage of closure. Indeed, PA’s inhibition of ABI2 may be more effective than Ca^2+^_c_ inhibition of ABI2 since Ca^2+^_c_ elevation cannot be sustained for an extended period because of the negative feedback loop with Ca^2+^ ATPase. Because PA-binding motifs are not highly conserved [47], the possibility that one or both of HAB1 and PP2CA are inhibited by PA also cannot be ruled out. The existence of alternative or additional putative mediators of an inhibitory effect from Ca^2+^_c_ to a PP2C also cannot be ruled out (see S3 Text). Denoting the putative mediator by X and considering, as an example, that the target PP2C is ABI2, there are two possible logic paths: Ca^2+^_c_ activates X and X inhibits ABI2, or Ca^2+^_c_ inhibits X, which is a required activator of ABI2. In the future, these new predictions can be further tested by a variety of wet bench approaches.

The hypothesized inhibition of additional PP2Cs by Ca^2+^_c_ introduces a discrepancy with observations in case of knockout of the node RCARs, which denotes the ABA receptors PYR/PYL/RCAR. In the presence of ABA, the combined knockout of multiple PYR/PYL/RCAR receptors is experimentally known to impose lack of ABA sensitivity, while the model simulations indicate hyposensitivity, reaching delayed closure (S13 Table). This discrepancy would decrease if one of the five ABA-regulated nodes with no known connections to RCARs, i.e. SPHK1/2, PI3P5K (PHOSPHATIDYLINOSITOL 3-PHOSPHATE 5-KINASE), PEPC (PHOSPHOENOLPYRUVATE CARBOXYLASE), Malate or AtRAC1 (which therefore are currently independent of RCARs by model logic) would be in fact RCARs-dependent. For example, if ABA’s inhibition of AtRAC1 were mediated by RCARs, knockout of RCARs would yield reduced ABA sensitivity in the model (see caption of S14 Table). These results therefore point toward prioritization of wet bench experiments designed to test the ABA-dependence of these nodes. Alternatively, it is possible that hyposensitivity rather than insensitivity to PYR/PYL/RCAR receptor knockout reflects biological reality. For example, a quadruple PYR/PYL/RCAR knockout retained partial sensitivity to ABA in inhibition of stomatal opening [55], suggesting possible involvement of other ABA receptors. Whether these receptors are other members of the PYR/PYL/RCAR family or a different type of receptor remains an open question [56,57].

While the majority of the experimental analysis to date focused on stomatal closure in the presence of ABA, which was consequently the focus of our previous model [4] and logic backbone analysis [16], here we analyzed the conditions that allow closure in the absence of ABA. Our finding of inhibition of the PP2C proteins by Ca^2+^_c_ fills a knowledge gap: augmenting the previous model with this finding restores agreement with experimental observations of closure in response to externally supplied Ca^2+^_,_ PA, NO, S1P, cADPR, or InsP3, constitutive activity of PLDδ, or externally induced Ca^2+^_c_ oscillations. The augmented models can serve as a foundation for future analysis of any signal or perturbation that yields stomatal closure.

For example, one interesting outcome of the augmented models is that they yield an increased baseline percentage of closure (e.g., 21.5% in Fig 3B), indicating that in a fraction of simulations the stable motif associated with closure is activated despite the absence of ABA and of constitutive activation of any nodes. In our models, seventeen nodes are initialized in a randomly selected state, simply because there is a lack of experimental data regarding their state in open stomata (see Methods). We identified and summarized in Fig 7B the multiple feedback mechanisms through which trajectories that start from initial activation of certain nodes (or inactivation in case of AtRAC1) may ultimately lead to closure. Future investigation of the resting state of these nodes will clarify the biological probability of these trajectories.

The augmented models and their analyses in response to various Ca^2+^_c_ patterns provide insight into the mechanism through which sustained Ca^2+^_c_ oscillations (whether regular or irregular) can drive the system to closure. Namely, the models suggest that repeated Ca^2+^_c_ increase can be translated into a stable activation pattern through multiple self- or mutually reinforcing positive feedback loops (see Figs 2B and 7). Our computational results are consistent with experimental results indicating that defined Ca^2+^_c_ oscillations are important for maintaining the closed state of stomata [11]. Our analysis implies that, if via other signaling pathways e.g. due to high CO_2_ [58,59], pathogenic elicitors [50,51], or methyl jasmonate [60] the guard cell shows suitable Ca^2+^_c_ patterns, it will likely stabilize in the closed stomate state. In depth elucidation of the temporal and quantitative aspects of Ca^2+^_c_ dynamics will require a tight integration of experimental and computational investigation.

## Methods

### Network reduction

Source nodes are defined as nodes that regulate (have outgoing edges to) other nodes but themselves have no documented regulation, i.e. have no incoming edges. The original model contains 22 source nodes that are assumed to maintain a constant state (e.g. constant presence of a certain molecule). As proven in [18], elimination of a source node by substituting the assumed constant state into the regulatory function of its target(s) preserves the attractor repertoire of Boolean networks. Our previous study of the logic structure of a Boolean network [16] implies that this reduction does not affect the trajectories of the unperturbed system. We used logic analysis to identify the cases where the effects of perturbing a source node (e.g. the effect of depleting a normally abundant molecule) are equally captured by perturbing its target node(s). We only reduce a source node if the reduction preserves agreement with experimental evidence on perturbing the source node. There are two cases of source nodes that we reduce in this work; in each case we encode the reduced source node into the label of the resulting node. The first is the case where the source node has exactly one successor (i.e. exactly one outgoing edge). Here, the notation of the resultant node encodes whether the relationship from source node to the successor node in the original model is activating (positive) or inhibiting (negative). Examples: B[A] means that A is the source node and B is its target in the original model and there is a positive edge A → B; C[A,~ B] denotes that A and B are source nodes and C is their shared target with edges A → C and B–● C. The list of the 17 reduced source nodes in this category along with the logic implication of each on its target is shown in S2 Table. Second is the case where the source node has multiple outgoing edges. These source nodes are eliminated only if in the original model the observed effect of knockout (KO) or constitutive activation (CA) of the source node is logically equivalent to the effect of the combined perturbation of its successor nodes. We use lowercase letters to mark these cases. For example, if A is a source node that has outgoing edges to B and C, and A is assumed to be ON in the original model, then A is removed from the network and the label B is changed to B{a} and the label C is changed to C{a}. These cases are detailed in S3 Table.

Following [18] and [16], we use two ways of reducing internal nodes of the network while preserving the underlying logic of the system and hence conserving the dynamics. One of these methods operates by merging two nodes related by an equivalence relationship, that is, an edge A → B such that A is the only regulator of B and B is the only successor of A. For example, f_ADPRc_ = 8-nitro-cGMP, which means that the state of ADPRc follows the state of 8-nitro-cGMP with a time delay. These two nodes can be collapsed into one node, which we denote as 8-nitro-cGMP → ADPRc. Perturbation (i.e. knockout or constitutive activation) of the resulting node equivalently represents the respective perturbation of each of the merged nodes. The 8 node pairs in this category are detailed in S4 Table. The other reduction method for internal nodes focuses on the case where a node has only one regulator, but the regulator has multiple outgoing edges. An example is an edge A → B where A is the only regulator of B, but A has successors other than B. A and B are merged into A[→B]. The set of successors (targets) of the new node A[→B] is the union of the set of successors of A and the set of successors of B while the regulators of A[→B] are the same as the regulators of A. This reduction is made only if experimental observations support that the effect of perturbing node A is qualitatively the same as the effect of perturbing node B. The three cases in this category, along with the references to experiments, are described in S5 Table. In addition to these reduction methods, we also merged the source nodes CPK6 and CPK23 into one node named CPK6/23 because they have the same successor nodes.

### Network components

The strongly connected component (SCC) of a network represents a subnetwork in which every pair of nodes A and B has both a path from A to B and a path from B to A. The in-component of the SCC is the set of nodes that can reach the SCC through paths, and the out-component is the set of nodes that can be reached from the SCC through paths. For example, ABA is in the in-component of the SCC shown in Fig 2 while Closure is in its out-component.

### Simulations

The simulations were executed using the Python library BooleanNet [61]. The SBML and BooleanNet files of all the model versions, along with the python code used to run the simulations, are available on the github repository /parulm/Stomata_PP2Cs. The initial state of the nodes in the reduced model is derived from the initial state of the nodes in [4], and reflects the existing knowledge regarding open stomata. Thirty-two of the 49 nodes are initialized in a particular state (ON or OFF); these nodes, their states, and the justification for the initial state are listed in S1 Text. The remaining 17 nodes, namely AnionEM, AtRAC1, cADPR, DAG, Depolarization, GHR1, KEV, KOUT, PEPC, PLC, PLDδ, QUAC1, SLAC1, SLAH3, TCTP, V-ATPase, V-PPase, are randomly initialized as ON or OFF. With these states as the starting point, the states of the nodes of the network are updated asynchronously, in a randomly selected order for 50 consecutive time steps. The order of node update is chosen randomly at each time step, giving equal probability to all update orders [62]. Update of a node means that the state of the node (ON or OFF) is updated using the Boolean regulatory function (update rule), evaluated using the current states of the regulator nodes. The Boolean update rules of the reduced network are given in S1 Text. Each additional inhibition is implemented by adding a term to the update rule of the inhibited node. Specifically, inhibition by Ca^2+^_c_ is implemented by adding “and not Ca^2+^_c_” to the update rule of the target node, and inhibition by PA is implemented by adding “and not PA” to the update rule. We performed 4500 simulations, each starting with a randomly selected state of the above mentioned 17 nodes. Although this number of simulations covers a small fraction of the total possible states visited by the system, we verified that this amount of sampling is sufficient to reflect the decisions made by the system. We determined the fraction of simulations in which the node Closure is in the ON state at each time step. For easier understanding we represent this fraction as a percentage and refer to it as the percentage of closure.

To characterize the nine nodes whose state oscillates in the long-term dynamics of the system, we used an ensemble of 4000 simulations of 100 time steps. We determined the length of their ON and OFF periods (i.e. the number of consecutive time steps for which the node is ON or OFF, respectively) in the last 40 time steps of the simulation, when the system is certain to have reached the relevant attractor. We then calculated the average duration of the ON and OFF period, respectively. For example, in the particular Ca^2+^_c_ time series [1, 1, 0, 1, 1, 0, 1, 0, 1, 1] the average ON period is 1.75 time steps and the average OFF period is 1 time step.

### Simulation of node constitutive activation and knockout

The constitutive activation (CA) of a node is simulated by setting its state to ON and keeping it fixed to ON throughout the entire simulation–this can be thought of as equivalent to providing a biomolecule in non-limiting quantity or, for an enzyme, providing the enzyme in non-limiting quantity and in the active state, during an experiment. The knockout (KO) of a node is simulated by setting its state to OFF and maintaining the OFF state throughout the experiment. A separate set of 4500 simulations was run for each of the different fixed states (constitutive activation or knockout) of various nodes. These in silico mutations are categorized into different response categories by their steady state percentage of closure and by their cumulative percentage of closure (CPC) values. The CPC is the summed fraction of simulations in which Closure = ON over the 50 timesteps–this is identical to the area under the curve in the simulation results plot on, for example, Fig 3.

To classify the various responses to node constitutive activation or knockout, we used our previously defined categories [4]. To evaluate the variance in the simulation results of the unperturbed system, we performed 10 sets of simulations (where each set consisted of 4500 simulations over 50 time-steps) and determined the mean and standard deviation of the percentage of closure and CPC. These values inform the range of the close to wild type (or close to baseline) response category. In applying this criterion, we also considered the natural gaps in the results and the logical implications in the model.

In the simulations in the presence of ABA, hypersensitive refers to the case when the simulation reaches 100% closure faster (in fewer time steps) compared to the simulation of the unperturbed (wild type) system. Close to wild type is the response category when the CPC lies within two standard deviations of the average wild type CPC. Hyposensitivity refers to reaching 100% closure more slowly than the wild type system. Reduced sensitivity to ABA is the case when the percentage of closure stabilizes at a value much less than 100%, while insensitivity means 0% closure in all simulations over all time-steps. In the simulations in the absence of ABA, we refer to the scenario where no node is kept constitutively active as the baseline. As 17 nodes in the simulation start from a random state, the stable motif corresponding to Closure = ON may have a chance to stabilize, and so can lead to a small non-zero percentage of closure in the baseline case. Significantly increased response is the case when the percentage of closure reaches 100%. Slightly increased response is the case when the final percentage of closure is higher than the baseline but less than 100%; these values are generally in the 40–60% range. Close to baseline is the case when the percentage of closure and CPC are within two standard deviations of the respective baseline values. Decreased response is noted when the percentage of closure is less than the baseline. Significantly decreased response is the case when there is no closure, i.e., the CPC is 0.

### Evaluation of the consistency between the simulated and experimentally-observed effect of constitutive activation or knockout of a node

Hypersensitivity to ABA is considered consistent with experimental observation of an increased degree of stomatal closure (i.e., aperture decrease) compared to wild type at the same concentration of ABA. Close to wild type response to ABA in a simulation is considered consistent with stomatal closure that is not statistically significantly different from the response of the wild type at the same concentration of ABA. Because the variability of experimental conditions does not allow a ranking of reductions in closure response, hyposensitivity, reduced sensitivity and insensitivity to ABA are all considered consistent with experimental observation of decreased stomatal closure compared to wild type.

As the few experimental studies performed in the absence of ABA focus on stomatal closure, they do not allow a clear separation into more than two response categories. Accordingly, we group the response categories into two broad classes: closure (which includes slightly and significantly increased response compared to baseline) and absence of closure (which includes close to baseline response, decreased response and significantly decreased response compared to baseline, i.e., opening). Slightly or significantly increased response compared to baseline are considered consistent with experimental observation of stomatal closure (decreased aperture compared to the wild type) in the absence of ABA. Close to baseline response, decreased and significantly decreased response are considered consistent with a stomatal aperture measured in the absence of ABA that is not statistically significantly different from, or is greater than that of the wild type.

### Stable motif, driver node, and attractor analysis

Stable motifs can be identified by finding strongly connected components (SCCs) in an expanded network that expresses the regulatory function of each node [19]. We used the software library available on https://github.com/jgtz/StableMotifs to determine the stable motifs of all the model versions and the attractors determined by these stable motifs. We verified the obtained attractors by simulations. We used the logic theory developed in [16] and the associated algorithms, available on https://github.com/parulm/suff_necc, to determine the internal and external drivers of stable motifs.

### Plant materials

*Arabidopsis thaliana* Columbia-0 (Col-0) plants were grown on soil (Fafard mix) under 12-h light/12-h dark photoperiod at 23/20°C, 65% humidity, and 75 μmol/m^2^s light intensity for four weeks. A total of 20 expanded leaf 5 and leaf 6 were cut into 0.5 mm strips using a new razor blade and digested in 10 ml enzyme solution (1% cellulase R10, 0.3% macerozyme R10 from Yakult-Japan, 0.4 M mannitol, 20 mM KCl, 20 mM MES, pH 5.7, 10 mM CaCl2, 1 mM β-mercaptoethanol and 0.1% BSA, Sigma A6793) in the dark for 3 h to generate mesophyll protoplasts as previously described [63].

### PP2C assays

To prepare phosphorylated casein substrate, casein kinase 1δ (1 μg, aa 1–294, Sigma, #14–520, 10 μg/10 μl) and 300 μCi gamma-^32^P-ATP (3000Ci/mmol, PerkinElmer, BLU002001MC) in 30 μl were added to 170 μl reaction mix (50 mM Tris-HCl pH 7.5, 10 mM MgCl_2_, 5 mM DTT, 200 μg casein, Sigma, #C4765), and the reaction was incubated at 30°C for 1 h. Casein kinase was inactivated by heating at 70oC for 10 min. After casein kinase inactivation, 10 volumes of cold 20% TCA were added, kept on ice for 10 min for protein precipitation, and then spun for 1 min in a Denville 260D microfuge at 12 Krpm. The supernatant was removed and the pellet, containing radiolabeled casein (^32^P-casein), was rinsed with 300 μl 20% TCA, followed by brief centrifugation to remove TCA. The pellet was resuspended in 200 μl buffer (200 mM Tris, pH 7.5 and 1 mM EDTA) and transferred to a G-25 Column. The G-25 column was placed in a 2.0 ml tube and spun for 30 sec at 600 X g at room temperature to remove free gamma-^32^P-ATP. The final concentration of ^32^P-casein was ~1 μg/μl [41].

For the preparation of cell lysates, mesophyll protoplasts (2 ml, 4 x 105) were settled in 10 ml WI (0.5 M mannitol, 20 mM KCl, 4 mM MES, pH 5.7) solution in a 10 cm Petri dish for 30 min at room temperature. Protoplasts were collected by centrifugation at 100 X g for 2 min, and washed with 2 ml MMG (0.4 M mannitol, 15 mM MgCl_2_, 4 mM MES, pH 5.7) solution three times to remove extracellular calcium. To prepare the cell lysate for the PP2C assay, 100 μl of lysis buffer (20 mM Tris-HCl, pH 7.5, 20 mM KCl, 1 mM EDTA, 10 mM DTT, 0.5% Triton X-100, 50% glycerol, Roche protease inhibitor cocktail) was added to the protoplast pellet and vortexed for 5 sec. Lysis was allowed to proceed for 15 min on ice [41].

For each PP2C reaction, 2.5 μl of protoplast lysate was added in 22.5 μl reaction mix (50 mM Tris-HCl pH 7.5, 10 mM MgCl_2_, 5 mM DTT, 0.1 μg ^32^P-casein with various CaCl_2_ concentrations, or 1 μM Okadaic acid or 100 nM Calyculin A (to inhibit PP1 and PP2A), followed by incubation in a shaker at 30oC, 600 rpm for 50 min. After the reaction, 2 ml cold 20% TCA was added, the reaction was kept on ice for 10 min, and then spun for 1 min in a Denville 260D microfuge at 12 Krpm and all supernatant, containing free ^32^P (released from ^32^P-casein by PP2C), was removed. The casein pellet was resuspended in 1 ml scintillation cocktail (UNIVERSOL^TM^-ESLIQUID, MP Biomedicals) and each sample was counted for 3 min in a Beckman LS6500 multi-purpose scintillation Counter [41].

## Supporting information

S1 Table The full names corresponding to the abbreviated node names in the ABA induced closure network, reproduced from [4] for convenience. (DOCX)

S2 Table List of cases where a source node that has a single successor (target) node is reduced and merged with its successor node. (DOCX)

S3 Table List of cases where the reduced source node has multiple target nodes. (DOCX)

S4 Table Node pairs connected by an equivalence relationship in the original model that are merged during reduction. (DOCX)

S5 Table List of node pairs that are merged where the regulator is the only regulator of the target, and the regulator also has additional outgoing edges. (DOCX)

S6 Table Composition of the in-component, the strongly connected component, and out-component of the reduced network. (DOCX)

S7 Table Attractors of the reduced model in the presence and absence of ABA. (DOCX)

S8 Table The effect of simulated node interventions (knockout or constitutive activation) in the presence of ABA in the full model supports the validity of network reduction. (DOCX)

S9 Table Comparison of the outcomes of the full and reduced models in the presence of ABA in cases of node knockout or constitutive activation for which there is known experimental evidence. (DOCX)

S10 Table Comparison of the outcomes of the full and reduced models in the presence of ABA for all possible cases of node knockout or constitutive activation. (DOCX)

S11 Table Comparison of the outcomes of the full and reduced model in the absence of ABA for all possible cases of node knockout or constitutive activation. (DOCX)

S12 Table Simulation results for constitutive activation of each node with CPC values when Ca^2+^c inhibits different PP2Cs. (DOCX)

S13 Table Attractors of the model version where Ca^2+^c directly inhibits ABI2 in the presence and absence of ABA. (DOCX)

S14 Table Simulation results for node knockout and constitutive activation for which experimental results exist, in the presence of ABA in the model version where Ca^2+^c directly inhibits ABI2. (DOCX)

S15 Table Simulation results for constitutive activation of each node when PA inhibits different PP2Cs. (DOCX)

S1 Fig Stable motifs of the reduced model. (TIF)

S1 Text Boolean update rules and initial states used in the simulations of the reduced model. (DOCX)

S2 Text Average ON/OFF time periods of nodes driven by a negative feedback loop. (DOCX)

S3 Text Justification of the hypothesized inhibitory edges(s) and analysis of alternative possibilities. (DOCX)

S4 Text Exploration of the effect of Ca^2+^c patterns on stomatal closure in the model version where Ca^2+^c inhibits ABI2. (DOCX)

S5 Text Theoretical analysis of the ABA-independent closure-inducing stable motif in case of Ca^2+^c -initiated inhibition of the PP2Cs. (DOCX)

S6 Text Exploration of the types of Ca^2+^c patterns that can drive closure in the absence of ABA in the model version where PA inhibits ABI2. (DOCX)

S7 Text Generation and maintenance of Ca^2+^c oscillations in the absence of ABA. (DOCX)
