# Supplementary material for: Model-driven discovery of calcium-related protein-phosphatase inhibition in plant guard cell signaling
Source: PLoS Comput Biol. 2019 Oct 28;15(10):e1007429. doi: 10.1371/journal.pcbi.1007429 (PMC6837631; doi:10.1371/journal.pcbi.1007429)
Supplement: S2 Table — (DOCX) [file pcbi.1007429.s002.docx]

**Table S2.** **List of cases where a source node that has a single successor (target) node is reduced and merged with its successor node.**

The first column indicates the source node that is eliminated. The second column indicates the assumed fixed state of the source node in the original model, based on the available experimental evidence. During reduction, this constant state is substituted in the regulatory function of the successor node. The notation of the merged node (fourth column) represents the nature and logic implication (defined in [1]) of the edge between the source and its successor node (given in the third column). Here s = sufficient, n = necessary, si=sufficient inhibitor, ni= necessary inhibitor, sn= sufficient and necessary, and m = neither sufficient nor necessary on its own but has a logical implication in combination with other regulators. Reduction of B→A is denoted A[B] and reduction of B–●A is denoted A[~B]. The experimental evidence of experimental perturbation of the source node, i.e. knockout (KO) or constitutive activation (CA), is listed in the fifth column. Since these source nodes do not exist independently in the reduced network, for each of the cases where there are experiments about the source node, we also identify the logically equivalent observation regarding the source node’s successor (sixth column). In the cases where there is no equivalent experiment possible (because there is no logic implication) we list and cite the experiment that has the closest effect as the source node KO or CA (last column). Overall, the experimental observations regarding the reduced source nodes are equivalently reflected in 7 cases and are closely approximated in two cases (GAPC1/2 and MRP5). There are no experimental observations in 7 cases.

| **The source node that is eliminated** | **State of the source node assumed in [2]** | **The edge that is collapsed** | **Notation of the merged node** | **Experimental evidence regarding the eliminated source node** | **Logically equivalent observation** | **Observed outcome of the closest experiment** |
| --- | --- | --- | --- | --- | --- | --- |
| ABH1 | ON | ABH1–●CaIM; ni | CaIM [~ABH1] | *ABH1* KO causes ABA hyper-sensitivity [3] | ABA hyper-sensitivity to constitutive CaIM |  |
| ARP complex | ON | ARP Complex → Actin Reorganization; n | Actin Reorganization [ARP Complex] | *ARP* Complex KO causes reduced sensitivity to ABA [4] | Loss of Actin Reorganization causes reduced sensitivity to ABA |  |
| DAGK | ON | DAGK → PA; m | PA [DAGK] | None | No equivalent experiment |  |
| ERA1 | ON | ERA1–●CaIM; ni | CaIM [~ABH1, ~ERA1] | *ERA1* KO causes ABA hypersensitivity [5] | CaIM CA causes ABA hypersensitivity |  |
| GAPC1/2 | ON | GAPC1/2 → PLDδ; m | PLDδ [GAPC1/2] | *GAPC* KO causes ABA hyposensitivity [6] | No equivalent experiment | *PLDδ* KO causes reduced sensitivity to ABA [6] |
| GCR1 | ON | GCR1–● GPA1; ni | GPA1 [~GCR1] | *GCR1* KO causes ABA hypersensitivity [7] | GPA1 CA causes ABA hypersensitivity |  |
| GEF1/4/10 | OFF | GEF 1/4/10 → ROP11; sn | ROP11 [GEF1/4/10] | CA of GEF1/4/10 causes ABA hyposensitivity [8,9] | CA of ROP11 causes ABA hyposensitivity [10] |  |
| GTP | ON | GTP → cGMP; n | cGMP [GTP] | None | cGMP KO gives the same response as GTP KO |  |
| MRP5 | ON | MRP5 → CaIM; m | CaIM [~ABH1, ~ERA1, MRP5] | *MRP5* KO causes ABA hyposensitivity [11] | No equivalent experiment | Loss of CaIM causes reduced sensitivity to ABA [12] |
| NAD^+^ | ON | NAD^+^→ cADPR; n | cADPR[NAD^+^] | None | cADPR KO gives the same results as NAD^+^ KO |  |
| Nitrite | ON | Nitrite → NO; n | NO [Nitrite] | None | NO KO gives the same response as Nitrite KO |  |
| NtSyp121 | ON | NtSyp121 → CaIM; m | CaIM [~ABH1, ~ERA1, MRP5, NtSyp121] | None | No equivalent experiment |  |
| PC | ON | PC → PA; m | PA [DAGK, PC] | None | No equivalent experiment |  |
| RCN1 | ON | RCN1 → RBOH; n | RBOH [RCN1] | *RCN1* KO causes reduced sensitivity to ABA [13] | *RBOH* KO causes reduced sensitivity to ABA [14] |  |
| SCAB1 | ON | SCAB1 → Actin Reorganization; n | Actin Reorganization [ARP Complex, SCAB1] | *SCAB1* KO yields reduced sensitivity to ABA [15] | Loss of Actin Reorganization yields reduced sensitivity to ABA |  |
| SPP1 | OFF | SPP1 –● S1P; si | S1P [~SPP1] | SPP1 KO yields equivalent to WT response [16] | No equivalent experiment |  |
| Sph | ON | Sph→ S1P; n | S1P [~SPP1, Sph] | None | Loss of S1P gives the same results as loss of Sph |  |

1. Maheshwari P, Albert R. A framework to find the logic backbone of a biological network. BMC Syst Biol. 2017;11(1):122.

2. Albert R, Acharya BR, Jeon BW, Zanudo JGT, Zhu M, Osman K, et al. A new discrete dynamic model of ABA-induced stomatal closure predicts key feedback loops. PLoS Biol. 2017;15(9):e2003451.

3. Hugouvieux V, Kwak JM, Schroeder JI. An mRNA cap binding protein, ABH1, modulates early abscisic acid signal transduction in Arabidopsis. Cell. 2001;106(4):477-87.

4. Jiang K, Sorefan K, Deeks MJ, Bevan MW, Hussey PJ, Hetherington AM. The ARP2/3 complex mediates guard cell actin reorganization and stomatal movement in Arabidopsis. Plant Cell. 2012;24(5):2031-40.

5. Pei ZM, Ghassemian M, Kwak CM, McCourt P, Schroeder JI. Role of farnesyltransferase in ABA regulation of guard cell anion channels and plant water loss. Science. 1998;282(5387):287-90.

6. Guo L, Devaiah SP, Narasimhan R, Pan X, Zhang Y, Zhang W, et al. Cytosolic glyceraldehyde-3-phosphate dehydrogenases interact with phospholipase Ddelta to transduce hydrogen peroxide signals in the Arabidopsis response to stress. Plant Cell. 2012;24(5):2200-12.

7. Pandey S, Assmann SM. The Arabidopsis putative G protein-coupled receptor GCR1 interacts with the G protein alpha subunit GPA1 and regulates abscisic acid signaling. Plant Cell. 2004;16(6):1616-32.

8. Li Z, Liu D. ROPGEF1 and ROPGEF4 are functional regulators of ROP11 GTPase in ABA-mediated stomatal closure in Arabidopsis. FEBS Lett. 2012;586(9):1253-8.

9. Yu F, Qian L, Nibau C, Duan Q, Kita D, Levasseur K, et al. FERONIA receptor kinase pathway suppresses abscisic acid signaling in Arabidopsis by activating ABI2 phosphatase. Proc Natl Acad Sci U S A. 2012;109(36):14693-8.

10. Li Z, Kang J, Sui N, Liu D. ROP11 GTPase is a negative regulator of multiple ABA responses in Arabidopsis. J Integr Plant Biol. 2012;54(3):169-79.

11. Suh SJ, Wang YF, Frelet A, Leonhardt N, Klein M, Forestier C, et al. The ATP binding cassette transporter AtMRP5 modulates anion and calcium channel activities in Arabidopsis guard cells. J Biol Chem. 2007;282(3):1916-24.

12. Cousson A. Two potential Ca(2+)-mobilizing processes depend on the abscisic acid concentration and growth temperature in the Arabidopsis stomatal guard cell. J Plant Physiol. 2003;160(5):493-501.

13. Kwak JM, Moon JH, Murata Y, Kuchitsu K, Leonhardt N, DeLong A, et al. Disruption of a guard cell-expressed protein phosphatase 2A regulatory subunit, RCN1, confers abscisic acid insensitivity in Arabidopsis. Plant Cell. 2002;14(11):2849-61.

14. Kwak JM, Mori IC, Pei ZM, Leonhardt N, Torres MA, Dangl JL, et al. NADPH oxidase AtrbohD and AtrbohF genes function in ROS-dependent ABA signaling in Arabidopsis. EMBO J. 2003;22(11):2623-33.

15. Zhao Y, Zhao S, Mao T, Qu X, Cao W, Zhang L, et al. The plant-specific actin binding protein SCAB1 stabilizes actin filaments and regulates stomatal movement in Arabidopsis. Plant Cell. 2011;23(6):2314-30.

16. Nakagawa N, Kato M, Takahashi Y, Shimazaki K, Tamura K, Tokuji Y, et al. Degradation of long-chain base 1-phosphate (LCBP) in Arabidopsis: functional characterization of LCBP phosphatase involved in the dehydration stress response. J Plant Res. 2012;125(3):439-49.
