# Supplementary material for: Model-driven discovery of calcium-related protein-phosphatase inhibition in plant guard cell signaling
Source: PLoS Comput Biol. 2019 Oct 28;15(10):e1007429. doi: 10.1371/journal.pcbi.1007429 (PMC6837631; doi:10.1371/journal.pcbi.1007429)
Supplement: S3 Table — (DOCX) [file pcbi.1007429.s003.docx]

**Table S3. List of cases where the reduced source node has multiple target nodes.**

Here, multiple nodes are affected by the elimination of the source node. The second column specifies the constant state of the source node in the original model. The third column lists all the target nodes of the corresponding source node and the label inside the parentheses denotes the logic implication of the edge from the source to the target (defined in [1]): s = sufficient; n = necessary and m = neither sufficient nor necessary on its own but has a logic implication in combination with other regulators. For convenience, these cases are marked by alphabetic notations, listed in column four. The fifth column lists the experimental evidence present in the literature for impacts of knockout (KO) or constitutive activation (CA) of the eliminated source node. Since the source node’s constant state affects multiple target nodes, the corresponding equivalent experiment (i.e. experiments that are expected to have the same effect in the logical framework) for the target nodes may be combinatorial. The expected results of these equivalent experiments are listed in the sixth column. We also indicate and cite logically close experiments in the last column.

| **Source** | **State of the source node** | **Targets of the source node, with edge type indicated in parentheses** | **Notation** | **Experimental evidence regarding the eliminated source node** | **Logically equivalent observation** | **Observed outcome of the closest experiment for the targets** |
| --- | --- | --- | --- | --- | --- | --- |
| NADPH | ON | ROS (n), NO[Nitrite] (n) | {a} | None | No equivalent experiment |  |
| PtdInsP3 | ON | RBOH[RCN1] (n), Actin Reorganization[ARP Complex, SCAB1] (m) | {b} | PtdInsP3 loss causes reduced sensitivity to ABA [2] | Double loss of *RBOH* and Actin Reorganization causes reduced sensitivity to ABA | *RBOH* KO causes reduced sensitivity to ABA [3] |
| PtdInsP4[→PtdIns(4,5)P2] | ON | InsP3[→InsP6] (n), DAG (n), Actin Reorganization[ARP_Complex,SCAB1] (m) | {c} | PtdInsP4 loss causes hyposensitivity to ABA [2] | Triple loss of InsP3, DAG and Actin Reorganization causes hyposensitivity to ABA | InsP3 loss causes hyposensitivity to ABA [4,5] |

1. Maheshwari P, Albert R. A framework to find the logic backbone of a biological network. BMC Syst Biol. 2017;11(1):122.

2. Jung JY, Kim YW, Kwak JM, Hwang JU, Young J, Schroeder JI, et al. Phosphatidylinositol 3- and 4-phosphate are required for normal stomatal movements. Plant Cell. 2002;14(10):2399-412.

3. Kwak JM, Mori IC, Pei ZM, Leonhardt N, Torres MA, Dangl JL, et al. NADPH oxidase AtrbohD and AtrbohF genes function in ROS-dependent ABA signaling in Arabidopsis. EMBO J. 2003;22(11):2623-33.

4. Hunt L, Mills LN, Pical C, Leckie CP, Aitken FL, Kopka J, et al. Phospholipase C is required for the control of stomatal aperture by ABA. Plant J. 2003;34(1):47-55.

5. Staxen I, Pical C, Montgomery LT, Gray JE, Hetherington AM, McAinsh MR. Abscisic acid induces oscillations in guard-cell cytosolic free calcium that involve phosphoinositide-specific phospholipase C. Proc Natl Acad Sci U S A. 1999;96(4):1779-84.
