# Supplementary material for: Model-driven discovery of calcium-related protein-phosphatase inhibition in plant guard cell signaling
Source: PLoS Comput Biol. 2019 Oct 28;15(10):e1007429. doi: 10.1371/journal.pcbi.1007429 (PMC6837631; doi:10.1371/journal.pcbi.1007429)
Supplement: S4 Table — (DOCX) [file pcbi.1007429.s004.docx]

**Table S4. Node pairs connected by an equivalence relationship in the original model that are merged during reduction.**

All these edges are activating, e.g. A → B, where the regulatory function of B is f_B_ = A, which means that A is both sufficient and necessary for B. In addition, B is the sole target of A. The merged node is labeled as “A→B” (third column). Any experiments that perturbed the state of the regulator node are equivalently reflected by the identical perturbation of the merged node. The fourth column lists and cites the experiments that perturb the eliminated regulator node. The fifth column lists the corresponding equivalent experiment in the reduced network involving the merged node. The sixth column lists and cites experiments that perturb the target node and the seventh column lists the equivalent experiment in the reduced network. There is a single experimental observation out of 18 (namely, close to wild type ABA sensitivity in case of GPA1) not equivalently reflected in the reduced network. This discrepancy is also present in the full model; it is not due to the reduction.

| **Regula-tor node** | **Target node** | **Notation of the merged node** | **Experimental evidence for the eliminated regulator node** | **Logically equivalent observation** | **Experimental evidence for the target node** | **Logically equivalent observation** |
| --- | --- | --- | --- | --- | --- | --- |
| PtdIns(3,5)P2 | V-PPase | PtdIns(3,5)P2→V-PPase | PtdIns(3,5)P2 loss causes hyposensitivity to ABA [1] | PtdIns(3,5)P2→V-PPase KO causes hyposensitivity to ABA | V-PPase KO causes ABA hyposensitivity [1] | PtdIns(3,5)P2→V-PPase KO causes hyposensitivity to ABA |
| 8-nitro-cGMP | ADPRc | 8-nitro-cGMP→ADPRc | None | None | ADPRc KO causes hyposensitivity to ABA [2,3] | 8-nitro-cGMP→  ADPRc KO causes hyposensitivity to ABA |
| S1P[~SPP1,Sph] | GPA1[~GCR1] | S1P[~SPP1,Sph]→GPA1[~GCR1] | S1P CA causes hypersensitivity to ABA [4,5] | S1P[~SPP1,Sph]→GPA1[~GCR1] CA causes hyper-sensitivity to ABA | *GPA1* KO causes close to WT stomatal closure response to ABA [6] | S1P[~SPP1,Sph]→  GPA1[~GCR1] KO causes close to WT response to ABA |
| NOGC1 | cGMP[GTP] | NOGC1→cGMP[GTP] | *NOGC1* KO causes hyposensitivity to ABA [7] | NOGC1→cGMP[GTP] KO causes hyposensitivity to ABA | cGMP CA causes close to WT response to ABA [7] | NOGC1→cGMP[GTP] CA causes close to WT response to ABA |
| PI3P5K | PtdIns35P2→V-Ppase | PI3P5K→PtdIns35P2→V-PPase | *PI3P5K* KO causes hyposensitivity to ABA [1] | PI3P5K→PtdIns(3,5)P2→V-Ppase KO causes hyposensitivity to ABA | V-PPase KO causes hyposensitivity to ABA [1] | PI3P5K→PtdIns(3,5)P2→V-Ppase KO causes hyposensitivity to ABA |
| 8-nitro-cGMP→  ADPRc | cADPR{a} | 8-nitro-cGMP→  ADPRc→  cADPR{a} | ADPRc KO causes hyposensitivity to ABA [2,3] | 8-nitro-cGMP→  ADPRc→cADPR{a} KO causes hyposensitivity to ABA | cADPR KO causes hyposensitivity to ABA [2] | 8-nitro-cGMP→  ADPRc→cADPR{a} KO causes hyposensitivity to ABA |
| RBOH[RCN1]{b} | ROS{a} | RBOH[RCN1]{b}→ROS{a} | RBOH/RCN1 KO causes reduced sensitivity to ABA [8-10] | RBOH[RCN1]{b}→ROS{a} KO causes reduced sensitivity to ABA | ROS KO causes reduced sensitivity to ABA [10] | RBOH[RCN1]{b}→ROS{a} KO causes reduced sensitivity to ABA |
| SPHK1/2 | S1P[~SPP1,Sph]→  GPA1[~GCR1] | SPHK1/2→S1P[~SPP1,Sph]→[~SPP1,Sph]→GPA1[~GCR1] | SPHK1/2 CA causes hypersensitivity to ABA [11,12];  SPHK1/2 KO causes reduced sensitivity to ABA [11,12] | SPHK1/2→S1P[~SPP1,Sph]→[~SPP1,Sph]→GPA1[~GCR1] CA causes hyper-sensitivity to ABA;  SPHK1/2→S1P[~SPP1,Sph]→[~SPP1,Sph]→GPA1[~GCR1] KO causes reduced sensitivity to ABA | S1P CA causes hypersensitivity to ABA [4,5];  GPA1 KO causes close to WT response to ABA [6] | SPHK1/2→S1P[~SPP1,Sph]→[~SPP1,Sph]→GPA1[~GCR1] CA causes hypersensitivity to ABA;  SPHK1/2→S1P[~SPP1,Sph]→[~SPP1,Sph]→GPA1[~GCR1] KO causes close to WT response to ABA |

1. Bak G, Lee EJ, Lee Y, Kato M, Segami S, Sze H, et al. Rapid structural changes and acidification of guard cell vacuoles during stomatal closure require phosphatidylinositol 3,5-bisphosphate. Plant Cell. 2013;25(6):2202-16.

2. Leckie CP, McAinsh MR, Allen GJ, Sanders D, Hetherington AM. Abscisic acid-induced stomatal closure mediated by cyclic ADP-ribose. Proc Natl Acad Sci U S A. 1998;95(26):15837-42.

3. Sanchez JP, Duque P, Chua NH. ABA activates ADPR cyclase and cADPR induces a subset of ABA-responsive genes in Arabidopsis. Plant J. 2004;38(3):381-95.

4. Coursol S, Fan LM, Le Stunff H, Spiegel S, Gilroy S, Assmann SM. Sphingolipid signalling in Arabidopsis guard cells involves heterotrimeric G proteins. Nature. 2003;423(6940):651-4.

5. Ng CKY, Carr K, McAinsh MR, Powell B, Hetherington AM. Drought-induced guard cell signal transduction involves sphingosine-1-phosphate. Nature. 2001;410(6828):596-9.

6. Wang XQ, Ullah H, Jones AM, Assmann SM. G protein regulation of ion channels and abscisic acid signaling in Arabidopsis guard cells. Science. 2001;292(5524):2070-2.

7. Joudoi T, Shichiri Y, Kamizono N, Akaike T, Sawa T, Yoshitake J, et al. Nitrated cyclic GMP modulates guard cell signaling in Arabidopsis. Plant Cell. 2013;25(2):558-71.

8. Albert I, Thakar J, Li S, Zhang R, Albert R. Boolean network simulations for life scientists. Source code for biology and medicine. 2008;3(1):16.

9. Kwak JM, Moon JH, Murata Y, Kuchitsu K, Leonhardt N, DeLong A, et al. Disruption of a guard cell-expressed protein phosphatase 2A regulatory subunit, RCN1, confers abscisic acid insensitivity in Arabidopsis. Plant Cell. 2002;14(11):2849-61.

10. Kwak JM, Mori IC, Pei ZM, Leonhardt N, Torres MA, Dangl JL, et al. NADPH oxidase AtrbohD and AtrbohF genes function in ROS-dependent ABA signaling in Arabidopsis. EMBO J. 2003;22(11):2623-33.

11. Guo L, Mishra G, Markham JE, Li M, Tawfall A, Welti R, et al. Connections between sphingosine kinase and phospholipase D in the abscisic acid signaling pathway in Arabidopsis. J Biol Chem. 2012;287(11):8286-96.

12. Worrall D, Liang YK, Alvarez S, Holroyd GH, Spiegel S, Panagopulos M, et al. Involvement of sphingosine kinase in plant cell signalling. Plant J. 2008;56(1):64-72.
