# Supplementary material for: Model-driven discovery of calcium-related protein-phosphatase inhibition in plant guard cell signaling
Source: PLoS Comput Biol. 2019 Oct 28;15(10):e1007429. doi: 10.1371/journal.pcbi.1007429 (PMC6837631; doi:10.1371/journal.pcbi.1007429)
Supplement: S5 Table — (DOCX) [file pcbi.1007429.s005.docx]

**Table S5. List of node pairs that are merged where the regulator is the only regulator of the target, and the regulator also has additional outgoing edges.**

For example, the node A has edges to B, C and D while the node C has only one regulator – the node A. Hence, we merge nodes A and C and use the notation “A[→C]” (see the third column for the specific cases). All the regulators of A are now the regulators of this new node A[→C] and the successors of this node are B, D, and all the successors of node C. Any experiments that perturbed the state of the regulator node (fourth column) is equivalently reflected by a perturbation of the merged node (fifth column). The sixth column cites experimental perturbations of the target node and the seventh column lists the equivalent experiment in the reduced network. In all the cases the merged node consistently unites the experimental observations for the merged regulator and target node.

| **Regula-tor node** | **Target node** | **Notation of the merged node** | **Experimental evidence for regulator** | **Logically equivalent observation** | **Experimental evidence for target** | **Logically equivalent experiment** |
| --- | --- | --- | --- | --- | --- | --- |
| InsP3 | InsP6 | InsP3[→InsP6] | InsP3 loss causes hyposensitivity to ABA [1,2] | InsP3[→InsP6] loss causes hyposensitivity to ABA | None | None |
| PtdInsP4 | PtdIns(4,5)P2 | PtdInsP4[→PtdIns(4,5)P2] | PtdInsP4 loss causes hyposensitivity to ABA [3] | PtdInsP4[→PtdIns(4,5)P2] loss causes hyposensitivity to ABA | PtdIns(4,5)P2 KO causes hypo-sensitivity to ABA [3,4] | PtdInsP4[→PtdIns(4,5)P2] KO causes hyposensitivity to ABA |
| OST1 | PIP2;1 | OST1[→PIP2;1] | *OST1* KO causes insensitivity to ABA [5,6] | OST1[→PIP2;1] KO causes insensitivity to ABA | Aquaporin (*PIP2;1*) KO causes insensitivity to ABA [7] | OST1[→PIP2;1] KO causes insensitivity to ABA |

1. Hunt L, Mills LN, Pical C, Leckie CP, Aitken FL, Kopka J, et al. Phospholipase C is required for the control of stomatal aperture by ABA. Plant J. 2003;34(1):47-55.

2. Staxen I, Pical C, Montgomery LT, Gray JE, Hetherington AM, McAinsh MR. Abscisic acid induces oscillations in guard-cell cytosolic free calcium that involve phosphoinositide-specific phospholipase C. Proc Natl Acad Sci U S A. 1999;96(4):1779-84.

3. Jung JY, Kim YW, Kwak JM, Hwang JU, Young J, Schroeder JI, et al. Phosphatidylinositol 3- and 4-phosphate are required for normal stomatal movements. Plant Cell. 2002;14(10):2399-412.

4. Bak G, Lee EJ, Lee Y, Kato M, Segami S, Sze H, et al. Rapid structural changes and acidification of guard cell vacuoles during stomatal closure require phosphatidylinositol 3,5-bisphosphate. Plant Cell. 2013;25(6):2202-16.

5. Li J, Wang XQ, Watson MB, Assmann SM. Regulation of abscisic acid-induced stomatal closure and anion channels by guard cell AAPK kinase. Science. 2000;287(5451):300-3.

6. Mustilli AC, Merlot S, Vavasseur A, Fenzi F, Giraudat J. Arabidopsis OST1 protein kinase mediates the regulation of stomatal aperture by abscisic acid and acts upstream of reactive oxygen species production. Plant Cell. 2002;14(12):3089-99.

7. Grondin A, Rodrigues O, Verdoucq L, Merlot S, Leonhardt N, Maurel C. Aquaporins Contribute to ABA-Triggered Stomatal Closure through OST1-Mediated Phosphorylation. Plant Cell. 2015;27(7):1945-54.
