# Supplementary material for: Model-driven discovery of calcium-related protein-phosphatase inhibition in plant guard cell signaling
Source: PLoS Comput Biol. 2019 Oct 28;15(10):e1007429. doi: 10.1371/journal.pcbi.1007429 (PMC6837631; doi:10.1371/journal.pcbi.1007429)
Supplement: S6 Table — (DOCX) [file pcbi.1007429.s006.docx]

**Table S6. Composition of the in-component, the strongly connected component, and the out-component of the reduced network.**

The largest strongly connected component (SCC) is the feedback-rich central part of the network. The in-component contains all the nodes that can reach the SCC via paths, and the out-component contains all nodes that can be reached from the SCC via paths. ABA is part of the in-component and Closure is part of the out-component. Two nodes do not belong to any of the three components: PEPC, which is regulated directly by ABA and cannot reach the SCC, and CPK6/23, which is a source node that cannot reach the SCC. The vast majority of paths that start at ABA and end in Closure, including all positive paths, pass through the SCC.

| **Component** | **Node count** | **Nodes** |
| --- | --- | --- |
| In-component | 4 | ABA, RCARs, ROP11, V-PPase |
| Strongly connected component | 27 | ABI1, ABI2, Actin Reorganization, AtRAC1, Ca^2+^_c_, Ca^2+^ ATPase, cADPR, CaIM, cGMP, CIS, DAG, GHR1, HAB1, InsP3/6, NIA1/2, NO, OST1, PA, pH_c_, PLC, PLDα, PLDδ, PP2CA, ROS, S1P, Vacuolar Acidification, V-ATPase |
| Out-component | 16 | AnionEM, Closure, CPK3/21, Depolarization, H_2_O Efflux, H^+^ ATPase, K^+^ Efflux, KEV, KOUT, Malate, MPK 9/12, Microtubule Depolymerization, QUAC1, TCTP, SLAH3, SLAC1 |
