# Supplementary material for: Model-driven discovery of calcium-related protein-phosphatase inhibition in plant guard cell signaling
Source: PLoS Comput Biol. 2019 Oct 28;15(10):e1007429. doi: 10.1371/journal.pcbi.1007429 (PMC6837631; doi:10.1371/journal.pcbi.1007429)
Supplement: S7 Table — (DOCX) [file pcbi.1007429.s007.docx]

**Table S7.  Attractors of the reduced model in the presence and absence of ABA.**

These attractors are equivalent to the attractors of the full model [1]. The nodes in orange font stabilize in a state opposite to their initial state (corresponding to open stomata) and the nodes in light green font stabilize in their initial state. The nodes that oscillate in the presence of ABA are driven by the negative feedback loop formed by Ca^2+^_c_ and Ca^2+^ ATPase. These nodes fall into three groups based on the average duration of a period of sustained ON or OFF state (see Methods and Text S2 for explication of those three groups). The nodes that oscillate in the absence of ABA are driven by the negative feedback loop between Depolarization and KOUT.

| **Long-term dynamics in the presence of ABA** | **Node count** | **Nodes** |
| --- | --- | --- |
| Stabilized in the ON state | 30 | Actin reorganization, AnionEM, cADPR, CaIM, CIS, Closure, cGMP, CPK3/21, CPK6/23, Depolarization, GHR1, H_2_O Efflux, K^+^ Efflux, KEV, KOUT, MPK 9/12, Microtubule Depolymerization, NIA1/2, NO, OST1, pH_c_, V-PPase, PA, PLDδ, RCARs, ROS, S1P, SLAC1, SLAH3, Vacuolar Acidification |
| Stabilized in the OFF state | 9 | ABI1, ABI2, AtRAC1, HAB1, H^+^ ATPase, Malate, PEPC, PP2CA, ROP11 |
| Oscillates with average ON/OFF period of 1.33 time steps | 2 | Ca^2+^_c_, Ca^2+^ ATPase |
| Oscillates with average ON/OFF period of 1.77 time steps | 5 | PLC, PLDα, QUAC1, TCTP, V-ATPase |
| Oscillates with average ON/OFF period of 2 time steps | 2 | DAG, InsP3/6 |
| **Long-term dynamics in the absence of ABA (baseline)** | **Node count** | **Nodes** |
| Stabilized in the ON state | 8 | ABI2, AtRAC1, CPK6/23, H^+^ ATPase, HAB1, Malate, PEPC, PP2CA |
| Stabilized in the OFF state | 31 | ABI1, Actin Reorganization, AnionEM, Ca^2+^, cADPR, CaIM, Ca^2+^ ATPase, cGMP, CIS, Closure, DAG, GHR1, H_2_O Efflux, InsP3/6, NIA1/2, NO, OST1, QUAC1, ROS, pH_c_, PA, V-PPase, PLC, PLDα, PLDδ, RCARs, ROP11, SLAC1, S1P, TCTP, V-ATPase |
| Stabilized in either the ON or OFF state | 6 | CPK3/21, MPK9/12, KEV, Microtubule Depolymerization, SLAH3, Vacuolar Acidification |
| Stabilized in the OFF state or oscillates | 3 | Depolarization, KOUT, K^+^ Efflux |

1. Albert R, Acharya BR, Jeon BW, Zanudo JGT, Zhu M, Osman K, et al. A new discrete dynamic model of ABA-induced stomatal closure predicts key feedback loops. PLoS Biol. 2017;15(9):e2003451.
