# Supplementary material for: Model-driven discovery of calcium-related protein-phosphatase inhibition in plant guard cell signaling
Source: PLoS Comput Biol. 2019 Oct 28;15(10):e1007429. doi: 10.1371/journal.pcbi.1007429 (PMC6837631; doi:10.1371/journal.pcbi.1007429)
Supplement: S8 Table — (DOCX) [file pcbi.1007429.s008.docx]

**Table S8.** **The effect of simulated node interventions (knockout or constitutive activation) in the presence of ABA in the full model supports the validity of network reduction.**

There are 81 nodes in the full model [1], of which all but ABA and Closure are candidates for simulated intervention. Knockout (KO) is simulated by keeping the relevant node OFF and constitutive activation (CA) is simulated by keeping the node state ON. The first column indicates the various response categories, which are the same as in Table 3 of [1]. Specifically, “equivalent to wild type” marks interventions that maintain the state of a source node in the fixed state it is assumed to be in the model of [1] (e.g. providing a molecule that is already present in a sufficient amount). “Close to wild type” indicates interventions under which the percentage of closure reaches 100% in the same amount of time as the wild type system. “Hypersensitivity” and “hyposensitivity” mark the cases where the percentage of closure reaches 100% faster, or slower, respectively, than in the wild type system. “Reduced sensitivity” indicates interventions under which the percentage of closure stabilizes at a value less than 100%, and “insensitivity” marks interventions under which the percentage of closure is 0%. Groups of nodes that are merged into a single node in the reduced model are marked by the same number (indicated in parentheses). The cases of intervention where the merged node reflects a corresponding intervention on a node eliminated during the merger in a logically equivalent manner are marked by an appended “e” to the number; if there are two such cases they are also marked with “1” and “2”.

All the nodes in the first response category are source (unregulated) nodes, and are assumed to be in a fixed state in the original model. In the reduced model all of these nodes are eliminated by merging them into their target nodes. To keep the number of groups below 20, we did not mark with numbers the mergers for which there are no logically equivalent interventions (e.g. the merger of DAGK into PA). The scarcity of notations “e” in the first response category indicates that the reduced model eliminates the majority of the cases where intervention on a source node is equivalent with the absence of intervention. This is a minor loss of information as these simulated interventions were tautological. The vast majority of the non-tautological interventions (such as elimination of a molecule that would otherwise be present in a sufficient amount) yield the same result for logically equivalent interventions of the source node and its target(s). Examples include the groups marked with 1e to 9e and 11e. Two types of discrepancy exist. First when merging a source node into its target node that has other regulators, it is possible that KO of the source node yields a milder defect than KO of the target node. This happens for DAGK, GAPC1/2, MRP5, and NtSyp121, respectively, whose KO yields ABA hyposensitivity in the full model, while knockout of their respective target nodes, PA, PLDδ, or CaIM (which is regulated by both MRP5 and NtSyp121) yields decreased sensitivity. This is not surprising given the additional regulators of the target node. A second type of discrepancy is exhibited by groups 10e and 12e, when a source node has two targets, and the KO of the source node has the same effect with the KO of only one of the targets. For example, NADPH is a necessary regulator of both ROS and NO and the depletion of NADPH has the same effect as KO of ROS but the KO of NO has a much milder effect. This is expected since the complete effect of the KO of the source node would actually be the sum of the effects of KO of the target nodes.

We indicate with orange and green two representative chains of three nodes merged into a single node (see Tables S2-S5). The two shades of each color distinguish between the two interventions (KO and CA). The relevant intervention of all nodes in each group yields the same effect (e.g. ABA hypersensitivity for the three nodes of group 15e2), illustrating these interventions’ logical equivalence and justifying the merger of these nodes.

| **Response category** | **Identity of the node and its relevant manipulation** |
| --- | --- |
| Equivalent to wild type | GTP CA(6), Sph CA, SCAB1 CA(9), GEF1/4/10 KO(5e1), CPK6 CA(18e2), GAPC CA, Nitrite CA(7), GCR1 CA(4), MRP5 CA, RCN1 CA(8), NAD^+^  CA, PtdInsP3 CA(11), SPP1 KO, PtdInsP4 CA(12), PC CA, CPK23 CA(18e2), NADPH CA(10), ARP complex CA(2), ABH1 CA(1), NtSyp121 CA, ERA1 CA(3), DAGK CA |
| Hypersensitivity | Vacuolar Acidification CA, SphK1/2 CA(15e2), GPA1 CA(4e,15e2), PI3P5K CA(13e2), NIA1/2 KO, 8-nitro-cGMP CA(14e2), HAB1 KO, RCARs CA, H^+^ ATPase KO, V-ATPase CA, GCR1 KO(4e), PtdIns(3,5)P2 CA(13e2), Depolarization CA, TCTP CA, S1P/PhytoS1P CA(15e2), Microtubule Depolymerization CA, V-PPase CA(13e2) , ABI1 KO , OST1 CA(20e2), ABI2 KO , K^+^ Efflux CA, AtRAC1 KO , SLAH3 CA, ADPRc CA(14e2), cADPR CA(14e2), InsP3 CA(12, 19e2) , ERA1 KO(3e), Actin Reorganization CA(2), InsP6 CA(19e2), ABH1 KO(1e), CIS CA, PA CA, CaIM CA(1e, 3e), NIA1/2 CA, DAG CA(12), NO CA (7), PLDδ CA, PLDα CA, PLC CA , QUAC1 CA, Ca^2+^ ATPase KO, RBOH CA(8,17e2), GHR1 CA, ROS CA(10,17e2), SLAC1 CA , AnionEM CA, H_2_O Efflux CA, Ca^2+^_c_ CA |
| Close to wild type | NtSyp121 KO, CPK3/21 CA, MRP5 KO, InsP3 KO(12e, 19e1), pH_c_ CA, HAB1 CA, cGMP KO(6e,16e1), PEPC KO, V-ATPase KO, CPK6 KO(18e1), CPK23 KO(18e1), NOGC1 CA(16e2), Aquaporin(PIP2;1) CA(20e2), Malate KO, NO KO(7e,10e), ROP11 KO(5e1), KEV CA, cGMP CA(6,16e2), PEPC CA, PtdIns(4,5)P2 CA, Nitrite KO(7e), GTP KO(6e), KOUT CA, PP2CA KO, NOGC1 KO(16e1) |
| Hyposensitivity | ROP11 CA(5e2), GEF1/4/10 CA(5e2), QUAC1 KO, CPK3/21 KO, SLAH3 KO, PLC KO, PtdIns(4,5)P2 KO, PtdInsP4 KO(12e), DAG KO(12e), DAGK KO, PLDα KO, V-PPase KO(13e1), CIS KO, PtdIns(3,5)P2 KO(13e1), cADPR KO(14e1), PI3P5K KO(13e1), GAPC KO, NAD^+^ KO, ADPRc KO(14e1), MPK9/12 CA, InsP6 KO(19e1), 8-nitro-cGMP KO(14e1) |
| Reduced sensitivity | Vacuolar Acidification KO, pH_c_ KO, ABI2 CA, H^+^ ATPase CA, CaIM KO(1,3), GPA1 KO(4,15e1), MPK9/12 KO, PP2CA CA, S1P/PhytoS1P KO(15e1), SphK1/2 KO(15e1), PtdInsP3 KO(11e), GHR1 KO, SLAC1 KO, RCN1 KO(8e), Sph KO, ROS KO(10e,17e1), PA KO, RBOH KO(8e,11e,17e1), SPP1 CA, NADPH KO(10e), ARP complex KO(2e), AtRAC1 CA, Actin Reorganization KO(2e,9e), SCAB1 KO(9e), PC KO, PLDδ KO |
| Insensitivity | Malate CA, H_2_O Efflux KO, Microtubule Depolymerization KO, TCTP KO, K^+^ Efflux KO, KEV KO, AnionEM KO, KOUT KO, Aquaporin(PIP2;1) KO(20e1), OST1 KO(20e1), Depolarization KO, RCARs KO, ABI1 CA, Ca^2+^ ATPase CA, Ca^2+^ _c_ KO |

1. Albert R, Acharya BR, Jeon BW, Zanudo JGT, Zhu M, Osman K, et al. A new discrete dynamic model of ABA-induced stomatal closure predicts key feedback loops. PLoS Biol. 2017;15(9):e2003451.
