# Supplementary material for: Model-driven discovery of calcium-related protein-phosphatase inhibition in plant guard cell signaling
Source: PLoS Comput Biol. 2019 Oct 28;15(10):e1007429. doi: 10.1371/journal.pcbi.1007429 (PMC6837631; doi:10.1371/journal.pcbi.1007429)
Supplement: S9 Table — (DOCX) [file pcbi.1007429.s009.docx]

**Table S9. Comparison of the outcomes of the full and reduced models in the presence of ABA in cases of node knockout or constitutive activation for which there is known experimental evidence.**

There were 76 relevant experimental observations reported in [1], which translate to 48 experimental observations for nodes of the reduced model (see Tables S2-S5). The results of simulated knockout or constitutive activation are compared with the simulated outcomes for the full model [1] and relevant experiments are cited. The first column names the various response categories, which are the same as in Table 3 of [1] and the simulation procedure is the same, as explained in the Methods section. Wild type (WT) refers to the unperturbed system. To evaluate the variance in the results (due to the stochasticity present in the initial conditions and order of node updates, see Methods), we performed 10 sets of simulations, where each set consisted of 4500 simulations over 50 time-steps. The average cumulative percentage of closure (CPC) for the WT simulation was 44.33, with a standard deviation of 0.023. Hypersensitive refers to the case when the simulation reaches 100% closure faster (in fewer time steps) compared to the WT simulation. Close to WT is the response category when the CPC lies within two standard deviations of the average WT CPC. Hyposensitivity refers to reaching 100% closure (i.e. all simulations) more slowly than the WT. Reduced sensitivity is the case when not all simulations can reach closure, i.e., the percentage of closure stabilizes at a value much less than 100%, while insensitivity means 0% closure in all simulations over all time-steps. The second column lists the number of cases in each category. The perturbations in the third column are organized in increasing order of their CPC values within each category. The fourth column lists the CPC range of each category. There were four cases (of 48 comparisons) where the simulation of the reduced model did not agree with the simulation of the full model in [1], these are listed in the last column. For each entry in this column, the response category according to the full model is listed in parentheses. In all the cases the full and reduced models agree that 100% closure will eventually be reached; the disagreement is only in the prediction whether the time to reach closure is slightly longer, slightly shorter, or close to the time to closure of the wild type system. This disagreement is likely due to the slightly different degree of stochasticity in the two models. The full model initializes 26 of the 80 nodes randomly (33%), while the reduced model initializes 17 of 49 nodes randomly (35%). We note that in two cases the reduced model agrees better with experimental results than the full model. For example, the reduced model predicts that NIA1/2 knockout (KO) (in boldface) leads to a close to WT sensitivity to ABA. This agrees with experiments [2], while its response category originally predicted in [1], namely hypersensitive, disagreed with experimental results. Nodes that are reduced or merged are underlined and their complete name and composition are given in Tables S2-S5.

| Response category | Number of cases | Cases of node KO or CA in this response category that agree with the full model | CPC range (0-50) | Cases of disagreement with the full model |
| --- | --- | --- | --- | --- |
| Hypersensitive | 9 | RCARs CA [3], TCTP CA [4], Microtubule Depolymerization CA [5,6], ABI2 KO [7,8], OST1 CA [9], ABI1 KO [7,8], PA CA [10], PLDα CA [10,11] | 44.44-44.8 | **PP2CA KO** (close to WT) [12] |
| Close to wild type | 8 | cGMP CA [13], NO KO [2], ROP11 KO [14], V-ATPase KO [15,16], cGMP KO [13] | 44.27-44.37 | S1P CA (hyper) [17-20], **NIA1/2 KO** (hyper) [2], HAB1 KO (hyper) [21,22] |
| Hyposensitive | 9 | ROP11 CA [16], QUAC1 KO [23,24], SLAH3 KO [25], V-PPase KO [15], PLC KO [26,27], PLDα KO [11], CIS KO [28,29], cADPR KO [30], InsP3/6 KO [26,27] | 41.36-44.15 |  |
| Reduced sensitivity | 15 | Vacuolar Acidification KO [15], CaIM KO [31], pH_c_ KO [32], ABI2 CA [33], Actin Reorganization KO [34], AtRAC1 CA [35], H^+^ATPase CA [36], GHR1 KO [37], ROS KO [38], SLAC1 KO [39], S1P KO [17-20], PA KO [10], PP2CA CA [12], MPK9/12 KO [40], PLDδ KO [41] | 10.28-38.12 |  |
| Insensitive | 7 | RCARs KO [42,43], K^+^efflux KO [44], OST1 KO [45,46], KOUT KO [44], Microtubule Depolymerization KO [6], Ca^2+^_c_ KO [28,29], ABI1 CA [33] | 0-0.01 |  |

1. Albert R, Acharya BR, Jeon BW, Zanudo JGT, Zhu M, Osman K, et al. A new discrete dynamic model of ABA-induced stomatal closure predicts key feedback loops. PLoS Biol. 2017;15(9):e2003451.

2. Desikan R, Griffiths R, Hancock J, Neill S. A new role for an old enzyme: nitrate reductase-mediated nitric oxide generation is required for abscisic acid-induced stomatal closure in Arabidopsis thaliana. Proc Natl Acad Sci U S A. 2002;99(25):16314-8.

3. Lim CW, Lee SC. Arabidopsis abscisic acid receptors play an important role in disease resistance. Plant molecular biology. 2015;88(3):313-24.

4. Du Z, Aghoram K, Outlaw Jr WH, Biophysics. In VivoPhosphorylation of Phosphoenolpyruvate Carboxylase in Guard Cells ofVicia fabaL. Is Enhanced by Fusicoccin and Suppressed by Abscisic Acid. Archives of Biochemistry. 1997;337(2):345-50.

5. Eisinger W, Ehrhardt D, Briggs W. Microtubules are essential for guard-cell function in Vicia and Arabidopsis. Molecular plant. 2012;5(3):601-10.

6. Jiang Y, Wu K, Lin F, Qu Y, Liu X, Zhang Q. Phosphatidic acid integrates calcium signaling and microtubule dynamics into regulating ABA-induced stomatal closure in Arabidopsis. Planta. 2014;239(3):565-75.

7. Gosti F, Beaudoin N, Serizet C, Webb AA, Vartanian N, Giraudat J. ABI1 protein phosphatase 2C is a negative regulator of abscisic acid signaling. The Plant Cell. 1999;11(10):1897-909.

8. Merlot S, Gosti F, Guerrier D, Vavasseur A, Giraudat J. The ABI1 and ABI2 protein phosphatases 2C act in a negative feedback regulatory loop of the abscisic acid signalling pathway. The Plant Journal. 2001;25(3):295-303.

9. Acharya BR, Jeon BW, Zhang W, Assmann SM. Open Stomata 1 (OST1) is limiting in abscisic acid responses of Arabidopsis guard cells. New Phytologist. 2013;200(4):1049-63.

10. Jacob T, Ritchie S, Assmann SM, Gilroy S. Abscisic acid signal transduction in guard cells is mediated by phospholipase D activity. Proceedings of the National Academy of Sciences. 1999;96(21):12192-7.

11. Mishra G, Zhang W, Deng F, Zhao J, Wang X. A bifurcating pathway directs abscisic acid effects on stomatal closure and opening in Arabidopsis. Science. 2006;312(5771):264-6.

12. Kuhn JM, Boisson-Dernier A, Dizon MB, Maktabi MH, Schroeder JI. The protein phosphatase AtPP2CA negatively regulates abscisic acid signal transduction in Arabidopsis, and effects of abh1 on AtPP2CA mRNA. Plant physiology. 2006;140(1):127-39.

13. Joudoi T, Shichiri Y, Kamizono N, Akaike T, Sawa T, Yoshitake J, et al. Nitrated cyclic GMP modulates guard cell signaling in Arabidopsis. Plant Cell. 2013;25(2):558-71.

14. Li Z, Gao X, Chinnusamy V, Bressan R, Wang ZX, Zhu JK, et al. ROP11 GTPase negatively regulates ABA signaling by protecting ABI1 phosphatase activity from inhibition by the ABA receptor RCAR1/PYL9 in Arabidopsis. J Integr Plant Biol. 2012;54(3):180-8.

15. Bak G, Lee EJ, Lee Y, Kato M, Segami S, Sze H, et al. Rapid structural changes and acidification of guard cell vacuoles during stomatal closure require phosphatidylinositol 3,5-bisphosphate. Plant Cell. 2013;25(6):2202-16.

16. Li Z, Kang J, Sui N, Liu D. ROP11 GTPase is a negative regulator of multiple ABA responses in Arabidopsis. J Integr Plant Biol. 2012;54(3):169-79.

17. Coursol S, Fan LM, Le Stunff H, Spiegel S, Gilroy S, Assmann SM. Sphingolipid signalling in Arabidopsis guard cells involves heterotrimeric G proteins. Nature. 2003;423(6940):651-4.

18. Guo L, Mishra G, Markham JE, Li M, Tawfall A, Welti R, et al. Connections between sphingosine kinase and phospholipase D in the abscisic acid signaling pathway in Arabidopsis. J Biol Chem. 2012;287(11):8286-96.

19. Ng CKY, Carr K, McAinsh MR, Powell B, Hetherington AM. Drought-induced guard cell signal transduction involves sphingosine-1-phosphate. Nature. 2001;410(6828):596-9.

20. Worrall D, Liang YK, Alvarez S, Holroyd GH, Spiegel S, Panagopulos M, et al. Involvement of sphingosine kinase in plant cell signalling. Plant J. 2008;56(1):64-72.

21. Rubio S, Rodrigues A, Saez A, Dizon MB, Galle A, Kim T-H, et al. Triple loss of function of protein phosphatases type 2C leads to partial constitutive response to endogenous abscisic acid. Plant physiology. 2009;150(3):1345-55.

22. Saez A, Robert N, Maktabi MH, Schroeder JI, Serrano R, Rodriguez PL. Enhancement of abscisic acid sensitivity and reduction of water consumption in Arabidopsis by combined inactivation of the protein phosphatases type 2C ABI1 and HAB1. Plant physiology. 2006;141(4):1389-99.

23. Meyer S, Mumm P, Imes D, Endler A, Weder B, Al‐Rasheid KA, et al. AtALMT12 represents an R‐type anion channel required for stomatal movement in Arabidopsis guard cells. The Plant Journal. 2010;63(6):1054-62.

24. Sasaki T, Mori IC, Furuichi T, Munemasa S, Toyooka K, Matsuoka K, et al. Closing plant stomata requires a homolog of an aluminum-activated malate transporter. Plant & cell physiology. 2010;51(3):354-65.

25. Geiger D, Maierhofer T, AL-Rasheid KA, Scherzer S, Mumm P, Liese A, et al. Stomatal closure by fast abscisic acid signaling is mediated by the guard cell anion channel SLAH3 and the receptor RCAR1. Sci Signal. 2011;4(173):ra32-ra.

26. Hunt L, Mills LN, Pical C, Leckie CP, Aitken FL, Kopka J, et al. Phospholipase C is required for the control of stomatal aperture by ABA. Plant J. 2003;34(1):47-55.

27. Staxen I, Pical C, Montgomery LT, Gray JE, Hetherington AM, McAinsh MR. Abscisic acid induces oscillations in guard-cell cytosolic free calcium that involve phosphoinositide-specific phospholipase C. Proc Natl Acad Sci U S A. 1999;96(4):1779-84.

28. Siegel RS, Xue S, Murata Y, Yang Y, Nishimura N, Wang A, et al. Calcium elevation‐dependent and attenuated resting calcium‐dependent abscisic acid induction of stomatal closure and abscisic acid‐induced enhancement of calcium sensitivities of S‐type anion and inward‐rectifying K+ channels in Arabidopsis guard cells. The Plant Journal. 2009;59(2):207-20.

29. Webb AA, Larman MG, Montgomery LT, Taylor JE, Hetherington AM. The role of calcium in ABA‐induced gene expression and stomatal movements. The Plant Journal. 2001;26(3):351-62.

30. Leckie CP, McAinsh MR, Allen GJ, Sanders D, Hetherington AM. Abscisic acid-induced stomatal closure mediated by cyclic ADP-ribose. Proc Natl Acad Sci U S A. 1998;95(26):15837-42.

31. Cousson A. Two potential Ca(2+)-mobilizing processes depend on the abscisic acid concentration and growth temperature in the Arabidopsis stomatal guard cell. J Plant Physiol. 2003;160(5):493-501.

32. Wang XQ, Ullah H, Jones AM, Assmann SM. G protein regulation of ion channels and abscisic acid signaling in Arabidopsis guard cells. Science. 2001;292(5524):2070-2.

33. Allen GJ, Kuchitsu K, Chu SP, Murata Y, Schroeder JI. Arabidopsis abi1-1 and abi2-1 phosphatase mutations reduce abscisic acid–induced cytoplasmic calcium rises in guard cells. The Plant Cell. 1999;11(9):1785-98.

34. Jiang K, Sorefan K, Deeks MJ, Bevan MW, Hussey PJ, Hetherington AM. The ARP2/3 complex mediates guard cell actin reorganization and stomatal movement in Arabidopsis. Plant Cell. 2012;24(5):2031-40.

35. Lemichez E, Wu Y, Sanchez J-P, Mettouchi A, Mathur J, Chua N-H. Inactivation of AtRac1 by abscisic acid is essential for stomatal closure. Genes & development. 2001;15(14):1808-16.

36. MacRobbie EA, Smyth WD. Effects of fusicoccin on ion fluxes in guard cells. New phytologist. 2010;186(3):636-47.

37. Hua D, Wang C, He J, Liao H, Duan Y, Zhu Z, et al. A plasma membrane receptor kinase, GHR1, mediates abscisic acid-and hydrogen peroxide-regulated stomatal movement in Arabidopsis. The Plant Cell. 2012:tpc. 112.100107.

38. Kwak JM, Mori IC, Pei ZM, Leonhardt N, Torres MA, Dangl JL, et al. NADPH oxidase AtrbohD and AtrbohF genes function in ROS-dependent ABA signaling in Arabidopsis. EMBO J. 2003;22(11):2623-33.

39. Vahisalu T, Kollist H, Wang Y-F, Nishimura N, Chan W-Y, Valerio G, et al. SLAC1 is required for plant guard cell S-type anion channel function in stomatal signalling. Nature. 2008;452(7186):487.

40. Jammes F, Song C, Shin D, Munemasa S, Takeda K, Gu D, et al. MAP kinases MPK9 and MPK12 are preferentially expressed in guard cells and positively regulate ROS-mediated ABA signaling. Proceedings of the National Academy of sciences. 2009;106(48):20520-5.

41. Guo L, Devaiah SP, Narasimhan R, Pan X, Zhang Y, Zhang W, et al. Cytosolic glyceraldehyde-3-phosphate dehydrogenases interact with phospholipase Ddelta to transduce hydrogen peroxide signals in the Arabidopsis response to stress. Plant Cell. 2012;24(5):2200-12.

42. Gonzalez-Guzman M, Pizzio GA, Antoni R, Vera-Sirera F, Merilo E, Bassel GW, et al. Arabidopsis PYR/PYL/RCAR receptors play a major role in quantitative regulation of stomatal aperture and transcriptional response to abscisic acid. The Plant Cell. 2012:tpc. 112.098574.

43. Nishimura N, Sarkeshik A, Nito K, Park SY, Wang A, Carvalho PC, et al. PYR/PYL/RCAR family members are major in‐vivo ABI1 protein phosphatase 2C‐interacting proteins in Arabidopsis. The Plant Journal. 2010;61(2):290-9.

44. Hosy E, Vavasseur A, Mouline K, Dreyer I, Gaymard F, Porée F, et al. The Arabidopsis outward K+ channel GORK is involved in regulation of stomatal movements and plant transpiration. Proceedings of the National Academy of Sciences. 2003;100(9):5549-54.

45. Li J, Wang XQ, Watson MB, Assmann SM. Regulation of abscisic acid-induced stomatal closure and anion channels by guard cell AAPK kinase. Science. 2000;287(5451):300-3.

46. Merlot S, Mustilli AC, Genty B, North H, Lefebvre V, Sotta B, et al. Use of infrared thermal imaging to isolate Arabidopsis mutants defective in stomatal regulation. The plant journal. 2002;30(5):601-9.
