# Supplementary material for: Model-driven discovery of calcium-related protein-phosphatase inhibition in plant guard cell signaling
Source: PLoS Comput Biol. 2019 Oct 28;15(10):e1007429. doi: 10.1371/journal.pcbi.1007429 (PMC6837631; doi:10.1371/journal.pcbi.1007429)
Supplement: S10 Table — (DOCX) [file pcbi.1007429.s010.docx]

**Table S10. Comparison of the outcomes of the full and reduced models in the presence of ABA for all possible cases of node knockout or constitutive activation.**

There are 49 nodes in the reduced model, and 47 of these are candidates for intervention (all except the signal ABA and the outcome node Closure). Thus, the total number of interventions is 94. The results of simulated knockout (KO) or constitutive activation (CA) are compared with the simulated outcomes for the full model [1]. The first column names the various response categories, which are the same as in Table 3 of [1] and in Table S8, and the simulation procedure is the same, as explained in the Methods section. The second column indicates the number of cases of node knockout or constitutive activation in each response category and the third column lists these cases. Wild type (WT) refers to the unperturbed system. The cumulative percentage of closure (CPC) for the WT simulation was 44.28 and the standard deviation was 0.04. The perturbations in the third column are organized in increasing order of their CPC values within each category. The fourth column lists the CPC range of each categories. There were 10 cases (of a total of 94) where the simulation of the reduced model did not agree with the simulation of the full model in [1], these are marked in bold and the full model’s categorization is given in parenthesis. In all of these 10 cases the full and reduced models agree that 100% closure will eventually be reached; the disagreement is only in the prediction whether the time to reach closure is slightly longer, slightly shorter, or close to the time to closure of the wild type system. As noted in the caption of Table S9, in some of these cases the reduced model may reflect the biological reality better than the full model.

| Response category | Number of cases | Cases of node knockout (KO) or constitutive activation (CA) in this response category | CPC range (0-50) |
| --- | --- | --- | --- |
| Hypersensitive | 34 | Depolarization CA, H^+^ ATPase KO, RCARs CA, ABI2 KO, TCTP CA, **PP2CA KO** (close to WT), K^+^ efflux CA, Microtubule Depolymerization CA, OST1 CA, **KOUT CA** (close to WT), **CPK3/21 CA** (close to WT), SLAH3 CA, ABI1 KO, AtRAC1 KO, PLDδ CA, NO CA, PLDα CA, PA CA, NIA1/2 CA, DAG CA, InsP3/6 CA, cADPR CA, Actin Reorganization CA, PLC CA, QUAC1 CA, CaIM CA, CIS CA, Ca^2+^ ATPase KO, ROS CA, GHR1 CA, SLAC1 CA, AnionEM CA, Ca^2+^_c_ CA, H_2_O Efflux CA | 44.42-47.3 |
| Close to wild type | 19 | **NIA1/2 KO** (hyper), KEV CA, pH_c_ CA, HAB1 CA, NO KO, **V-ATPase CA** (hyper), Malate KO, PEPC KO, cGMP KO, ROP11 KO, **S1P CA** (hyper), cGMP CA, **HAB1 KO** (hyper), V-ATPase KO, **V-PPase CA** (hyper), CPK6/23 CA, **Vacuolar Acidification CA** (hyper), PEPC CA, **MPK 9/12CA** (hypo) | 44.25-44.39 |
| Hyposensitive | 12 | ROP11 CA, CPK6/23 KO, CPK3/21 KO, QUAC1 KO, SLAH3 KO, V-PPase KO, PLDα KO, PLC KO, DAG KO, CIS KO, cADPR KO, InsP3/6 KO | 41.38-44.16 |
| Reduced sensitivity | 15 | Vacuolar Acidification KO, CaIM KO, pH_c_ KO, ABI2 CA, AtRAC1 CA, Actin Reorganization KO, H^+^ ATPase CA, ROS KO, MPK9/12 KO, GHR1 KO, SLAC1 KO, PA KO, PP2CA CA, S1P KO, PLDδ KO | 10.55-38.08 |
| Insensitive | 14 | Depolarization KO, TCTP KO, OST1 KO, Malate CA, AnionEM KO, K^+^ efflux KO, Ca^2+^ ATPase CA, KEV KO, KOUT KO, H_2_O Efflux KO, RCARs KO, Microtubule Depolymerization KO, Ca^2+^_c_ KO, ABI1 CA | 0-0.01 |

1. Albert R, Acharya BR, Jeon BW, Zanudo JGT, Zhu M, Osman K, et al. A new discrete dynamic model of ABA-induced stomatal closure predicts key feedback loops. PLoS Biol. 2017;15(9):e2003451.
