# Supplementary material for: Model-driven discovery of calcium-related protein-phosphatase inhibition in plant guard cell signaling
Source: PLoS Comput Biol. 2019 Oct 28;15(10):e1007429. doi: 10.1371/journal.pcbi.1007429 (PMC6837631; doi:10.1371/journal.pcbi.1007429)
Supplement: S11 Table — (DOCX) [file pcbi.1007429.s011.docx]

**Table S11. Comparison of the outcomes of the full and reduced models in the absence of ABA for all possible cases of node knockout (KO) or constitutive activation (CA).**

As in Table S10, the total number of interventions is 94. The first column lists the response categories. Baseline is the case where no node is constitutively active or knocked out and ABA=OFF; this case yields 0% closure in all the simulations. “Same as baseline” is the case when the KO or CA of the node gives the same effect as baseline, i.e. 0% closure. “Increased compared to baseline” is the case when KO or CA of the node leads to a non-zero final percentage of closure. The node categorizations agree with the complete model. Albert et al. [1] used the additional category “Slightly increased response”, which had a small transient nonzero percentage of closure but final percentage of closure of 0%, and CPC <0.03. This category included constitutive activity of TCTP, Microtubule depolymerization, PLDδ, CIS, Ca^2+^_c_. We consider these results consistent with the “Same as baseline” category of the reduced model. The second column indicates the number of interventions in each response category and the third column lists the corresponding interventions. All the results of the reduced model agree the results of the full model.

| Response category | Number of cases | Cases of node KO or CA – all are consistent with the full model | CPC range |
| --- | --- | --- | --- |
| Same as baseline | 87 | PEPC KO, TCTP KO, SLAH3 KO, KEV KO, KOUT KO, PA CA, H_2_O Efflux KO, InsP3/6 KO, cADPR KO, Malate KO, PP2CA CA, CPK6/23 CA, Ca^2+^_c_ KO, SLAC1 KO, HAB1 CA, K^+^ efflux KO, ROP11 KO, GHR1 CA, DAG KO, Actin Reorganization CA, Actin Reorganization KO, AtRAC1 CA, ABI2 CA, QUAC1 KO, K^+^ efflux CA, NIA1/2 CA, Vacuolar Acidification KO, CPK3/21 CA, ABI1 KO, AnionEM CA, cGMP KO, RCARs KO, AtRAC1 KO, NO KO, V-PPase KO, CPK3/21 KO, CIS CA, V-PPase CA, SLAH3 CA, pH_c_ CA, CPK6/23 KO, pH_c_ KO, AnionEM KO, DAG CA, Ca^2+^_c_ CA, Microtubule Depolymerization CA, PLC CA, S1P KO, MPK9/12 KO, H^+^ ATPase KO, Ca^2+^ ATPase CA, Malate CA, H^+^ ATPase CA, KEV CA, Ca^2+^ ATPase KO, MPK9/12 CA, PLC KO, PA KO, CaIM CA, Vacuolar Acidification CA, Microtubule Depolymerization KO, PLDα CA, KOUT CA, ABI1 CA, V-ATPase KO, S1P CA, PLDδ KO, CaIM KO, PLDα KO, Depolarization CA, GHR1 KO, PLDδ CA, TCTP CA, cADPR CA, Depolarization KO, OST1 KO, SLAC1 CA, QUAC1 CA, CIS KO, cGMP CA, NO CA, PEPC CA, ROP11 CA, NIA1/2 KO, ROS KO, V-ATPase CA, InsP3/6 CA | 0.0-0.0 |
| Increased compared to baseline | 7 | HAB1 KO, PP2CA KO, OST1 CA, ABI2 KO, RCARs CA, H_2_O Efflux CA, ROS CA | 9.1-44.32 |

1. Albert R, Acharya BR, Jeon BW, Zanudo JGT, Zhu M, Osman K, et al. A new discrete dynamic model of ABA-induced stomatal closure predicts key feedback loops. PLoS Biol. 2017;15(9):e2003451.
