# Supplementary material for: Model-driven discovery of calcium-related protein-phosphatase inhibition in plant guard cell signaling
Source: PLoS Comput Biol. 2019 Oct 28;15(10):e1007429. doi: 10.1371/journal.pcbi.1007429 (PMC6837631; doi:10.1371/journal.pcbi.1007429)
Supplement: S12 Table — (DOCX) [file pcbi.1007429.s012.docx]

**Table S12. Simulation results for constitutive activation of each node in the absence of ABA when Ca^2+^_c_ inhibits different PP2Cs.**

There are 5 different simulation scenarios: Ca^2+^_c_ inhibits ABI1; Ca^2+^_c_ inhibits ABI2; Ca^2+^_c_ inhibits HAB1; Ca^2+^_c_ inhibits PP2CA; Ca^2+^_c_ inhibits all four PP2Cs (ABI1, ABI2, HAB1, and PP2CA). For each of these cases, there was a baseline case, that is, the case when no node is kept constitutively active. At the bottom of the table, highlighted in green, are the mean and standard deviation of the baseline percentage of closure and baseline cumulative percentage of closure (CPC) based on 10 groups of 4500 simulations (see Methods). We have classified the simulations for constitutive activation of different nodes in 5 different categories: close to the baseline (within two standard deviations), slightly increased, significantly increased (the cases where 100% closure is reached), decreased and significantly decreased – see the Methods section for more details on this. The CPC value of each simulation is indicated in brackets. For comparison we also include within the “Significantly increased” category the CPC values for ABA induced closure in the five scenarios. Bold and black font indicates the cases that are consistent with experimental observations while cases shown in bold and red font disagree with experiment (see Methods for a description of how we evaluate consistency). The literature references in each case are the same as those listed in Table 2 and hence are not repeated here. No experimental evidence exists for the cases shown with regular font. The results presented in the last four columns of this table are highly similar except for the expected variation in the CPC in the case of constitutive activation of a node that would be otherwise inhibited by Ca^2+^_c_.

| Category | Ca –●ABI1 | | Ca –●ABI2 | | Ca –●HAB1 | | Ca –● PP2CA | | Ca –● all | |
| --- | --- | --- | --- | --- | --- | --- | --- | --- | --- | --- |
| Close to baseline | AnionEM (0), CPK3/21 (0), QUAC1 (0), cGMP (0), SLAH3 (0), MPK9/12 (0), **TCTP (0)**, CPK6/23 (0), HAB1 (0), **ROP11 (0)**, PEPC (0), KEV (0), **Microtubule Depolymerization (0)**, K^+^efflux (0), Depolarization (0), KOUT (0), SLAC1 (0), PLDδ (0), V-PPase (0), **pH_c_ (0)**, **PLDα (0)**, DAG (0), Vacuolar Acidification (0), **PA (0)**, V-ATPase (0), **NO (0)**, NIA1/2 (0), **S1P (0), Ca^2+^_c_ (0)**, **InsP3/6 (0)**, PLC (0), GHR1 (0), CIS (0), **cADPR (0)**, CaIM (0), Actin Reorganization (0), **AtRAC1 (0)**, **H^+^ ATPase (0)**, **PP2CA (0), ABI2 (0)**, Ca^2+^ ATPase (0), **ABI1 (0)**, Malate (0) | | MPK9/12 (8.9), CPK6/23 (8.96), KEV (9.07), **TCTP (9.11),** HAB1 (9.16), SLAH3 (9.19), KOUT (9.23), CPK3/21 (9.24), cGMP (9.28), **ROP11 (9.33),** SLAC1 (9.34), PEPC (9.4), Depolarization (9.43), K^+^efflux (9.83), AnionEM (9.9), QUAC1 (9.9), **Microtubule Depolymerization (9.91),** | | Depolarization (6.07), K^+^ efflux (6.1), **Microtubule Depolymerization (6.2),** PEPC (6.29), KOUT (6.29), SLAH3 (6.3), cGMP (6.48), CPK3/21 (6.49), MPK (6.57), CPK6/23 (6.68), **ROP11 (6.71)**, KEV (6.86), AnionEM (7.05), **TCTP (7.14),** QUAC1 (7.14), V-ATPase (7.34), Vacuolar Acidification (7.4), SLAC1 (7.49) | | KEV (6.12), Depolarization (6.36), **ROP11 (6.42)**, CPK6/23 (6.5), KOUT (6.53), K^+^efflux (6.58), MPK9/12 (6.58), PEPC (6.59), HAB1 (6.62), cGMP (6.67), SLAH3 (6.74), CPK3/21 (6.77), **Microtubule Depolymerization (6.8), TCTP (6.85),** QUAC1 (7.08), AnionEM (7.12), V-ATPase (7.44), SLAC1 (7.44), Vacuolar Acidification (7.48), V-PPase (7.53), | | Depolarization (10.64), HAB1 (10.86), PEPC (10.91), CPK6/23 (10.91), KOUT (10.93), K^+^efflux (11.08), **ROP11 (11.14)**, **TCTP (11.17), Microtubule Depolymerization (11.19)**, SLAH3 (11.26), KEV (11.44), MPK9/12 (11.51), CPK3/21 (11.57), QUAC1 (11.92), NOGC1 (12.08), AnionEM (12.13), SLAC1 (12.31) | |
| Slightly increased | OST1 (9.65), RCARs (16.77), H_2_O Efflux (28.98) | | V-ATPase (10.34), **pH_c_ (10.42)**, Vacuolar Acidification (10.46), V-PPase (10.53), **S1P (11.97),** OST1 (12.17), **PA (13.8)**, DAG (14.43), NIA1/2 (14.49), PLDδ (14.75), **NO (14.49), PLDα (14.64)**, RCARs (16.83), H_2_O Efflux (29.69) | | V-PPase (7.99), **S1P (8.41),** OST1 (9.41), NIA1/2 (9.88), PLDδ (10.1), **NO (10.21), pH_c_ (10.35)**, **PLDα (10.51)**, **PA (10.53)**, DAG (10.95), RCARs (16.89), H_2_O Efflux (29.9) | | **S1P (8.64),** OST1 (9.24), NIA1/2 (9.65), **NO (9.76),** DAG (9.92), PLDδ (10.08), **PLDα (10.11)**, **PA (10.75)**, **pH_c_ (11.14)**, RCARs (17.29), H_2_O Efflux (30.15) | | OST1 (12.69), V-PPase (12.82), Vacuolar Acidification (12.84), V-ATPase (12.86), **S1P (14.06), pH_c_ (14.97)**, NIA1/2 (15.81), **PLDα (16.46)**, PLDδ (16.47), DAG (16.5), **NO (16.75),** RCARs (16.78), **PA (16.81)**, H_2_O Efflux (29.97) | |
| Significantly increased | ABA (44.33), **ROS (44.4)** | | **cADPR (37.6)**, Actin Reorganization (37.7), **InsP3/6 (37.83)**, GHR1 (37.94), CaIM (38.37), CIS (38.53), **ROS (39.94),** PLC (41.83), **Ca^2+^_c_ (41.97)**, ABA (44.31) | | Actin Reorganization (34.03), GHR1 (34.4), CaIM (34.82), CIS (35.11), PLC (39.86), **cADPR (39.93)**, **InsP3/6 (39.96)**, ABA (44.33), **ROS (44.38), Ca^2+^_c_ (46.09)** | | Actin Reorganization (34.49), GHR1 (34.79), CaIM (34.99), CIS (35.14), **InsP3/6 (39.94)**, **cADPR (39.96)**, PLC (40.01), ABA (44.32), **ROS (44.41), Ca^2+^_c_ (46.57)** | | Actin Reorganization (40.46), **cADPR (40.53)**, **InsP3/6 (40.55)**, CIS (41.29), CaIM (41.32), GHR1 (41.32), PLC (42.91), ABA (44.39), **ROS (44.40), Ca^2+^_c_ (46.63)** | |
| Decreased | - | | **PP2CA (3.39), H^+^ ATPase (4.64)**, **AtRAC1 (7.44)** | | **PP2CA (2.58), H^+^ ATPase (3.25), AtRAC1 (5.43)** | | **H^+^ ATPase (3.24), AtRAC1 (5.75)** | | **PP2CA (4.2), H^+^ATPase (5.71), AtRAC1 (9.41)** | |
| Significantly decreased | - | | Ca^2+^ ATPase (0.0), **ABI1 (0.0),** Malate (0.0), **ABI2 (0.0)** | | Ca^2+^ ATPase (0.0), **ABI1 (0.0),** HAB1 (0.0), Malate (0.0), **ABI2 (0.31)** | | Ca^2+^ ATPase (0.0), **ABI1 (0.0), PP2CA (0.0),** Malate (0.0), **ABI2 (0.29)** | | Ca^2+^ ATPase (0.0), **ABI1 (0.0**), Malate (0.0), **ABI2 (0.45)** | |
| Mean baseline % closure and CPC | 0% | 0 | 21.3% | 9.15 | 15.4% | 6.55 | 15.2% | 6.48 | 25.6% | 11.19 |
| Standard deviation of the baseline % closure and CPC | 0% | 0 | 0.8% | 0.34 | 0.53% | 0.22 | 0.47% | 0.2 | 0.48% | 0.21 |
