# Supplementary material for: Model-driven discovery of calcium-related protein-phosphatase inhibition in plant guard cell signaling
Source: PLoS Comput Biol. 2019 Oct 28;15(10):e1007429. doi: 10.1371/journal.pcbi.1007429 (PMC6837631; doi:10.1371/journal.pcbi.1007429)
Supplement: S13 Table — (DOCX) [file pcbi.1007429.s013.docx]

**Table S13. Attractors of the model version in which Ca^2+^_c_ directly inhibits ABI2 in the presence and absence of ABA.**

In this model version, three attractors are reachable when 17 nodes are initialized randomly (due to lack of information on their resting states). The attractor corresponding to closure in the presence of ABA is identical to the corresponding one in the original reduced model (top part of Table S7). There is a new attractor, corresponding to closure in the absence of ABA. This attractor differs from the ABA-induced closure attractor in the state of five nodes, shown in blue font. These nodes need the ABA signal to achieve their closure-associated states. The rest of the nodes can be stabilized by the autonomous dynamics of the system. In both attractors, the nodes that oscillate do so as a result of the oscillation of Ca^2+^_c_ (see Text S2). The attractor corresponding to lack of closure in the absence of ABA is identical to the corresponding attractor in the original reduced model (bottom part of Table S7). The nodes in orange font stabilize in a state opposite to their initial state (corresponding to open stomata) and the nodes in light green font stabilize in their initial state. Two of the five nodes whose final state differs from their state corresponding ABA-induced closure, namely RCARs and Actin Reorganization, stabilize in the same state as their initial states; the remaining three were initialized randomly so no such categorization can be made. The attractors corresponding to the model versions where Ca^2+^ directly inhibits HAB1 or PP2CA, or inhibits multiple PP2Cs, are the same as the ones reported in this table. In the model version where Ca^2+^ directly inhibits ABI1 the attractor corresponding to closure in the absence of ABA is not reachable from the specified initial condition (the same way as in the original reduced model). The attractors corresponding to the model versions where PA inhibits multiple PP2Cs are also the same as those shown in this table.

| **Attractor associated to closure in the presence of ABA** | **Node count** | **Nodes** |
| --- | --- | --- |
| Stabilized in the ON state | 30 | Actin reorganization, AnionEM, cADPR, CaIM, CIS, Closure, cGMP, CPK3/21, CPK6/23, Depolarization, GHR1, H_2_O Efflux, K^+^ Efflux, KEV, KOUT, MPK 9/12, Microtubule Depolymerization, NIA1/2, NO, OST1, pH_c_, V-PPase, PA, PLDδ, RCARs, ROS, S1P, SLAC1, SLAH3, Vacuolar Acidification |
| Stabilized in the OFF state | 9 | ABI1, ABI2, AtRAC1, HAB1, H^+^ ATPase, Malate, PEPC, PP2CA, ROP11 |
| Oscillates with average ON/OFF period of 1.33 time steps | 3 | Ca^2+^_c_, Ca^2+^ ATPase |
| Oscillates with average ON/OFF period of 1.77 time steps | 3 | PLC, PLDα, QUAC1, TCTP, V-ATPase |
| Oscillates with average ON/OFF period of 2 time steps | 3 | DAG, InsP3/6 |
| **Attractor associated to closure in the absence of ABA** | **Node count** | **Nodes** |
| Stabilized in the ON state | 29 | AnionEM, AtRAC1, cADPR, CaIM, CIS, Closure, cGMP, CPK3/21, CPK6/23, Depolarization, GHR1, H_2_O Efflux, K^+^ Efflux, KEV, KOUT, MPK 9/12, Microtubule Depolymerization, NIA1/2, NO, OST1, pH_c_, PA, PEPC, PLDδ, ROS, S1P, SLAC1, SLAH3, Vacuolar Acidification |
| Stabilized in the OFF state | 10 | Actin reorganization, ABI1, ABI2, HAB1, H^+^ ATPase, Malate, PP2CA, RCARs, ROP11, V-PPase |
| Oscillates with average ON/OFF period of 1.33 time steps | 2 | Ca^2+^_c_, Ca^2+^ ATPase |
| Oscillates with average ON/OFF period of 1.77 time steps | 5 | PLC, PLDα, QUAC1, TCTP, V-ATPase |
| Oscillates with average ON/OFF period of 2 time steps | 2 | DAG, InsP3/6 |
| **Attractor associated to lack of closure in the absence of ABA** | **Node count** | **Nodes** |
| Stabilized in the ON state | 8 | ABI2, AtRAC1, CPK6/23, H^+^ ATPase, HAB1, Malate, PEPC, PP2CA |
| Stabilized in the OFF state | 31 | ABI1, Actin Reorganization, AnionEM, Ca^2+^, cADPR, CaIM, Ca^2+^ ATPase, cGMP, CIS, Closure, DAG, GHR1, H_2_O Efflux, InsP3/6, NIA1/2, NO, OST1, QUAC1, ROS, pH_c_, PA, V-PPase, PLC, PLDα, PLDδ, RCARs, ROP11, SLAC1, S1P, TCTP, V-ATPase |
| Stabilized in either the ON or OFF state | 6 | CPK3/21, MPK9/12, KEV, Microtubule Depolymerization, SLAH3, Vacuolar Acidification |
| Stabilized in the OFF state or oscillates | 3 | Depolarization, KOUT, K^+^ Efflux |
