# Supplementary material for: Model-driven discovery of calcium-related protein-phosphatase inhibition in plant guard cell signaling
Source: PLoS Comput Biol. 2019 Oct 28;15(10):e1007429. doi: 10.1371/journal.pcbi.1007429 (PMC6837631; doi:10.1371/journal.pcbi.1007429)
Supplement: S14 Table — (DOCX) [file pcbi.1007429.s014.docx]

**Table S14. Simulation results for node knockout and constitutive activation for which experimental results exist, in the presence of ABA in the model version where Ca^2+^_c_ directly inhibits ABI2.**

There were 76 relevant experimental observations reported in [1], which translate to 48 experimental observations for nodes of the reduced model (see Tables S2-S5). Wild type (WT) refers to the unperturbed system, which, in this case includes the assumption that Ca^2+^_c_ inhibits ABI2. We performed 4500 simulations over 50 time-steps; the cumulative percentage of closure (CPC) for the WT simulation was 44.35 with a standard deviation of 0.04. The first column lists the response categories, which are defined as in Methods and Table S9. The second column lists the number of cases in each of the response categories. The third column lists the node perturbations that belong to this category in increasing order of CPC values. Node perturbations in bold font agree with experiments (with relevant references cited) while those in regular font disagree. The last column lists the CPC range corresponding to each of the response category. Most of the simulation results (39 of 48) agree with experiments, but there are 9 discrepancies. Eight of these discrepancies are shared with the original reduced model. The discrepancies are divided in two categories marked by *a* or *b* in parentheses. Type *a* refers to the cases where the model predicts lower sensitivity to ABA than experimental observation, for example the model predicts close to wild type response when experiments indicate ABA hypersensitivity. Type *b* indicates cases where the model predicts higher ABA sensitivity than experimental observation, for example close to wild type response when experiments indicate ABA hyposensitivity. There is a single discrepancy exhibited by this model version and not by the original reduced model, shown in italic font, referring to knockout of the node RCARs, which denotes the ABA receptors PYR/PYL/RCAR. In the presence of ABA, the combined knockout of multiple PYR/PYL/RCAR receptors is experimentally known to show ABA insensitivity [2,3], i.e., lack of stomatal closure despite ample supply of ABA, although a quadruple PYR/PYL/RCAR still shows partial sensitivity to ABA under some circumstances (inhibition of stomatal opening by ABA) [4]. The model simulations indicate hyposensitivity, eventually reaching closure in all simulations, albeit more slowly than the wild type system (in 30 time steps instead of 17). According to current knowledge incorporated in the model, ABA affects multiple nodes (S1P, AtRAC1, V-PPase, PEPC, Malate) without known connections to RCARs, and thus these influences will be maintained in the model even if RCARs are knocked out. The node AtRAC1, for example, being inhibited by ABA, will stabilize in the OFF state despite RCARs being knocked out. This leads to the ON state of the node CaIM via Actin Reorganization. CaIM is able to sustain Ca^2+^_c_ oscillations (see Text S3), which can lead to closure (see Text S4). If future wet bench research identifies RCAR-dependence of AtRAC1, the effect of RCARs’ knockout in a model updated to reflect this dependence would be equivalent to the combined effect of knockout of RCARs and constitutive activation of AtRAC1 in the current model, which yields reduced sensitivity to ABA (with final percentage of closure at 23% and CPC value of 10.24). Simultaneous KO of V-PPase, or constitutive activation of PEPC, or adaptation of the regulatory function of S1P or Malate (e.g. changing the function of Malate from Malate* = PEPC and not ABA and not AnionEM to Malate* = PEPC and not AnionEM) yields an additional minor reduction in ABA sensitivity. The model versions wherein Ca^2+^_c_ directly inhibits HAB1, PP2CA or multiple PP2Cs, or PA inhibits ABI2, HAB1, PP2CA or multiple PP2Cs, yield the same categorization of each node knockout and constitutive activation in the presence of ABA as this table. Thus, these model versions are equally supported by experimental observations.

| Response category | Number of cases | Cases of node knockout (KO) or constitutive activation (CA) in this response category | CPC range (0-50) |
| --- | --- | --- | --- |
| Hypersensitive | 9 | **RCARs CA** [5], **HAB1 KO** [6,7], **TCTP CA** [8], **ABI2 KO** [9,10], **OST1 CA** [11], Microtubule Depolymerization CA (b) [12,13], **ABI1 KO** [9,10], **PA CA** [14], **PLDα CA** [14,15] | 44.45-44.86 |
| Close to wild type | 8 | **NIA1/2 KO** [16], ROP11 KO (a) [17], S1P CA (a) [18-21], V-ATPase KO (b) [22,23], **cGMP** **CA** [24], cGMP KO (b) [24], NO KO (b) [16], PP2CA KO (a) [25] | 44.28-44.37 |
| Hyposensitive | 10 | *RCARs KO (b)* [2,3], **ROP11 CA** [23], SLAH3 KO (a) [26], **QUAC1 KO** [27,28], **V-PPase KO** [22], **PLDα KO** [15], **PLC KO** [29,30], **CIS KO** [31,32], **InsP3/6 KO** [29,30], **cADPR KO** [33,34] | 40.01-44.21 |
| Reduced sensitivity | 15 | **Vacuolar Acidification KO** [22], **CaIM KO** [35], **pH_c_ KO** [36], **ABI2 CA** [37], **Actin Reorganization KO** [38], **AtRAC1 CA** [39], **H^+^ ATPase CA** [40], **GHR1 KO** [41], **SLAC1 KO** [42], **S1P KO** [18-21]**, PA KO** [14], **ROS KO** [43], **PP2CA CA** [25], **MPK9/12 KO** [44], **PLDδ KO** [45] | 10.49-38.2 |
| Insensitive | 6 | **Ca^2+^_c_ KO** [31,46], **OST1 KO** [47,48], **K^+^ efflux KO** [49], **Microtubule Depolymerization KO** [13], **KOUT KO** [49], **ABI1 CA** [37] | 0-0.01 |

1. Albert R, Acharya BR, Jeon BW, Zanudo JGT, Zhu M, Osman K, et al. A new discrete dynamic model of ABA-induced stomatal closure predicts key feedback loops. PLoS Biol. 2017;15(9):e2003451.

2. Gonzalez-Guzman M, Pizzio GA, Antoni R, Vera-Sirera F, Merilo E, Bassel GW, et al. Arabidopsis PYR/PYL/RCAR receptors play a major role in quantitative regulation of stomatal aperture and transcriptional response to abscisic acid. The Plant Cell. 2012:tpc. 112.098574.

3. Nishimura N, Sarkeshik A, Nito K, Park SY, Wang A, Carvalho PC, et al. PYR/PYL/RCAR family members are major in‐vivo ABI1 protein phosphatase 2C‐interacting proteins in Arabidopsis. The Plant Journal. 2010;61(2):290-9.

4. Ye Y, Adachi Y, Ye W, Hayashi M, Nakamura Y, Kinoshita T, et al. Difference in abscisic acid perception mechanisms between closure induction and opening inhibition of stomata. Plant physiology. 2013:pp. 113.223826.

5. Lim CW, Lee SC. Arabidopsis abscisic acid receptors play an important role in disease resistance. Plant molecular biology. 2015;88(3):313-24.

6. Rubio S, Rodrigues A, Saez A, Dizon MB, Galle A, Kim T-H, et al. Triple loss of function of protein phosphatases type 2C leads to partial constitutive response to endogenous abscisic acid. Plant physiology. 2009;150(3):1345-55.

7. Saez A, Robert N, Maktabi MH, Schroeder JI, Serrano R, Rodriguez PL. Enhancement of abscisic acid sensitivity and reduction of water consumption in Arabidopsis by combined inactivation of the protein phosphatases type 2C ABI1 and HAB1. Plant physiology. 2006;141(4):1389-99.

8. Du Z, Aghoram K, Outlaw Jr WH, Biophysics. In VivoPhosphorylation of Phosphoenolpyruvate Carboxylase in Guard Cells ofVicia fabaL. Is Enhanced by Fusicoccin and Suppressed by Abscisic Acid. Archives of Biochemistry. 1997;337(2):345-50.

9. Gosti F, Beaudoin N, Serizet C, Webb AA, Vartanian N, Giraudat J. ABI1 protein phosphatase 2C is a negative regulator of abscisic acid signaling. The Plant Cell. 1999;11(10):1897-909.

10. Merlot S, Gosti F, Guerrier D, Vavasseur A, Giraudat J. The ABI1 and ABI2 protein phosphatases 2C act in a negative feedback regulatory loop of the abscisic acid signalling pathway. The Plant Journal. 2001;25(3):295-303.

11. Acharya BR, Jeon BW, Zhang W, Assmann SM. Open Stomata 1 (OST1) is limiting in abscisic acid responses of Arabidopsis guard cells. New Phytologist. 2013;200(4):1049-63.

12. Eisinger W, Ehrhardt D, Briggs W. Microtubules are essential for guard-cell function in Vicia and Arabidopsis. Molecular plant. 2012;5(3):601-10.

13. Jiang Y, Wu K, Lin F, Qu Y, Liu X, Zhang Q. Phosphatidic acid integrates calcium signaling and microtubule dynamics into regulating ABA-induced stomatal closure in Arabidopsis. Planta. 2014;239(3):565-75.

14. Jacob T, Ritchie S, Assmann SM, Gilroy S. Abscisic acid signal transduction in guard cells is mediated by phospholipase D activity. Proceedings of the National Academy of Sciences. 1999;96(21):12192-7.

15. Mishra G, Zhang W, Deng F, Zhao J, Wang X. A bifurcating pathway directs abscisic acid effects on stomatal closure and opening in Arabidopsis. Science. 2006;312(5771):264-6.

16. Desikan R, Griffiths R, Hancock J, Neill S. A new role for an old enzyme: nitrate reductase-mediated nitric oxide generation is required for abscisic acid-induced stomatal closure in Arabidopsis thaliana. Proc Natl Acad Sci U S A. 2002;99(25):16314-8.

17. Li Z, Gao X, Chinnusamy V, Bressan R, Wang ZX, Zhu JK, et al. ROP11 GTPase negatively regulates ABA signaling by protecting ABI1 phosphatase activity from inhibition by the ABA receptor RCAR1/PYL9 in Arabidopsis. J Integr Plant Biol. 2012;54(3):180-8.

18. Coursol S, Fan LM, Le Stunff H, Spiegel S, Gilroy S, Assmann SM. Sphingolipid signalling in Arabidopsis guard cells involves heterotrimeric G proteins. Nature. 2003;423(6940):651-4.

19. Guo L, Mishra G, Markham JE, Li M, Tawfall A, Welti R, et al. Connections between sphingosine kinase and phospholipase D in the abscisic acid signaling pathway in Arabidopsis. J Biol Chem. 2012;287(11):8286-96.

20. Ng CKY, Carr K, McAinsh MR, Powell B, Hetherington AM. Drought-induced guard cell signal transduction involves sphingosine-1-phosphate. Nature. 2001;410(6828):596-9.

21. Worrall D, Liang YK, Alvarez S, Holroyd GH, Spiegel S, Panagopulos M, et al. Involvement of sphingosine kinase in plant cell signalling. Plant J. 2008;56(1):64-72.

22. Bak G, Lee EJ, Lee Y, Kato M, Segami S, Sze H, et al. Rapid structural changes and acidification of guard cell vacuoles during stomatal closure require phosphatidylinositol 3,5-bisphosphate. Plant Cell. 2013;25(6):2202-16.

23. Li Z, Kang J, Sui N, Liu D. ROP11 GTPase is a negative regulator of multiple ABA responses in Arabidopsis. J Integr Plant Biol. 2012;54(3):169-79.

24. Joudoi T, Shichiri Y, Kamizono N, Akaike T, Sawa T, Yoshitake J, et al. Nitrated cyclic GMP modulates guard cell signaling in Arabidopsis. Plant Cell. 2013;25(2):558-71.

25. Kuhn JM, Boisson-Dernier A, Dizon MB, Maktabi MH, Schroeder JI. The protein phosphatase AtPP2CA negatively regulates abscisic acid signal transduction in Arabidopsis, and effects of abh1 on AtPP2CA mRNA. Plant physiology. 2006;140(1):127-39.

26. Geiger D, Maierhofer T, AL-Rasheid KA, Scherzer S, Mumm P, Liese A, et al. Stomatal closure by fast abscisic acid signaling is mediated by the guard cell anion channel SLAH3 and the receptor RCAR1. Sci Signal. 2011;4(173):ra32-ra.

27. Meyer S, Mumm P, Imes D, Endler A, Weder B, Al‐Rasheid KA, et al. AtALMT12 represents an R‐type anion channel required for stomatal movement in Arabidopsis guard cells. The Plant Journal. 2010;63(6):1054-62.

28. Sasaki T, Mori IC, Furuichi T, Munemasa S, Toyooka K, Matsuoka K, et al. Closing plant stomata requires a homolog of an aluminum-activated malate transporter. Plant & cell physiology. 2010;51(3):354-65.

29. Hunt L, Mills LN, Pical C, Leckie CP, Aitken FL, Kopka J, et al. Phospholipase C is required for the control of stomatal aperture by ABA. Plant J. 2003;34(1):47-55.

30. Staxen I, Pical C, Montgomery LT, Gray JE, Hetherington AM, McAinsh MR. Abscisic acid induces oscillations in guard-cell cytosolic free calcium that involve phosphoinositide-specific phospholipase C. Proc Natl Acad Sci U S A. 1999;96(4):1779-84.

31. Siegel RS, Xue S, Murata Y, Yang Y, Nishimura N, Wang A, et al. Calcium elevation‐dependent and attenuated resting calcium‐dependent abscisic acid induction of stomatal closure and abscisic acid‐induced enhancement of calcium sensitivities of S‐type anion and inward‐rectifying K+ channels in Arabidopsis guard cells. The Plant Journal. 2009;59(2):207-20.

32. Wang Y, Noguchi K, Ono N, Inoue S-i, Terashima I, Kinoshita T. Overexpression of plasma membrane H+-ATPase in guard cells promotes light-induced stomatal opening and enhances plant growth. Proceedings of the National Academy of Sciences. 2014;111(1):533-8.

33. Leckie CP, McAinsh MR, Allen GJ, Sanders D, Hetherington AM. Abscisic acid-induced stomatal closure mediated by cyclic ADP-ribose. Proc Natl Acad Sci U S A. 1998;95(26):15837-42.

34. Sanchez JP, Duque P, Chua NH. ABA activates ADPR cyclase and cADPR induces a subset of ABA-responsive genes in Arabidopsis. Plant J. 2004;38(3):381-95.

35. Cousson A. Two potential Ca(2+)-mobilizing processes depend on the abscisic acid concentration and growth temperature in the Arabidopsis stomatal guard cell. J Plant Physiol. 2003;160(5):493-501.

36. Wang XQ, Ullah H, Jones AM, Assmann SM. G protein regulation of ion channels and abscisic acid signaling in Arabidopsis guard cells. Science. 2001;292(5524):2070-2.

37. Allen GJ, Kuchitsu K, Chu SP, Murata Y, Schroeder JI. Arabidopsis abi1-1 and abi2-1 phosphatase mutations reduce abscisic acid–induced cytoplasmic calcium rises in guard cells. The Plant Cell. 1999;11(9):1785-98.

38. Jiang K, Sorefan K, Deeks MJ, Bevan MW, Hussey PJ, Hetherington AM. The ARP2/3 complex mediates guard cell actin reorganization and stomatal movement in Arabidopsis. Plant Cell. 2012;24(5):2031-40.

39. Lemichez E, Wu Y, Sanchez J-P, Mettouchi A, Mathur J, Chua N-H. Inactivation of AtRac1 by abscisic acid is essential for stomatal closure. Genes & development. 2001;15(14):1808-16.

40. MacRobbie EA, Smyth WD. Effects of fusicoccin on ion fluxes in guard cells. New phytologist. 2010;186(3):636-47.

41. Hua D, Wang C, He J, Liao H, Duan Y, Zhu Z, et al. A plasma membrane receptor kinase, GHR1, mediates abscisic acid-and hydrogen peroxide-regulated stomatal movement in Arabidopsis. The Plant Cell. 2012:tpc. 112.100107.

42. Vahisalu T, Kollist H, Wang Y-F, Nishimura N, Chan W-Y, Valerio G, et al. SLAC1 is required for plant guard cell S-type anion channel function in stomatal signalling. Nature. 2008;452(7186):487.

43. Kwak JM, Mori IC, Pei ZM, Leonhardt N, Torres MA, Dangl JL, et al. NADPH oxidase AtrbohD and AtrbohF genes function in ROS-dependent ABA signaling in Arabidopsis. EMBO J. 2003;22(11):2623-33.

44. Jammes F, Song C, Shin D, Munemasa S, Takeda K, Gu D, et al. MAP kinases MPK9 and MPK12 are preferentially expressed in guard cells and positively regulate ROS-mediated ABA signaling. Proceedings of the National Academy of sciences. 2009;106(48):20520-5.

45. Guo L, Devaiah SP, Narasimhan R, Pan X, Zhang Y, Zhang W, et al. Cytosolic glyceraldehyde-3-phosphate dehydrogenases interact with phospholipase Ddelta to transduce hydrogen peroxide signals in the Arabidopsis response to stress. Plant Cell. 2012;24(5):2200-12.

46. Webb AA, Larman MG, Montgomery LT, Taylor JE, Hetherington AM. The role of calcium in ABA‐induced gene expression and stomatal movements. The Plant Journal. 2001;26(3):351-62.

47. Li J, Wang XQ, Watson MB, Assmann SM. Regulation of abscisic acid-induced stomatal closure and anion channels by guard cell AAPK kinase. Science. 2000;287(5451):300-3.

48. Merlot S, Mustilli AC, Genty B, North H, Lefebvre V, Sotta B, et al. Use of infrared thermal imaging to isolate Arabidopsis mutants defective in stomatal regulation. The plant journal. 2002;30(5):601-9.

49. Hosy E, Vavasseur A, Mouline K, Dreyer I, Gaymard F, Porée F, et al. The Arabidopsis outward K+ channel GORK is involved in regulation of stomatal movements and plant transpiration. Proceedings of the National Academy of Sciences. 2003;100(9):5549-54.
