# Supplementary material for: Model-driven discovery of calcium-related protein-phosphatase inhibition in plant guard cell signaling
Source: PLoS Comput Biol. 2019 Oct 28;15(10):e1007429. doi: 10.1371/journal.pcbi.1007429 (PMC6837631; doi:10.1371/journal.pcbi.1007429)
Supplement: S15 Table — (DOCX) [file pcbi.1007429.s015.docx]

**Table S15. Simulation results for constitutive activity of each node when PA inhibits different PP2Cs.**

Comparison between experimental results and model simulation outcomes upon simulated constitutive activation (CA) of all nodes in the model for which corresponding experimental evidence on constitutive activation in the absence of ABA exists, under different assumptions regarding PA inhibition of PP2Cs. The first column lists the various response categories (see Methods). Simulated constitutive activation of the nodes shown in bold font agrees with experimental observations while the others do not. The agreement of the response categorizations in Table 2, Table S12 and this table shows that PA inhibiting the PP2Cs (two or more) has the same effect as Ca^2+^_c_ inhibiting the PP2Cs directly and is equally supported by experiments.

| Response category | No added inhibition | | PA –●ABI2 | | PA –● HAB1 | | PA –●PP2CA | | PA –●all | |
| --- | --- | --- | --- | --- | --- | --- | --- | --- | --- | --- |
| Close to baseline | **TCTP** **(0),** **ROP11** (**0), Microtubule Depolymerization (0),** Ca^2+^_c_ (0), InsP3/6 (0), cADPR (0), pH_c_ (0), **PLDα** (0), PA (0), NO (0), S1P (0), **AtRAC1 (0), H^+^ ATPase (0), PP2CA (0), ABI1 (0), ABI2 (0)** | | **ROP11 (12.55),**  **Microtubule Depolymerization (12.58), TCTP (12.81)** | | **Microtubule Depolymerization (7.45), TCTP (7.85), ROP11 (7.97)** | | **ROP11 (7.59), Microtubule Depolymerization (7.92), TCTP (8.17)** | | **ROP11 (13.51),**  **Microtubule Depolymerization (13.59), TCTP (13.76)** | |
| Slightly increased |  | | **S1P (14.93), pHc (15.94), PA (30.35), NO (30.39), PLDα (30.91)** | | **S1P (9.09), PLDα (13.23), PA (14.09), pHc (16.32), NO (13.65)** | | **S1P (9.2), PLDα (13.65), PA (14.09), pHc (15.74), NO (13.8)** | | **S1P (16.2), pHc (18.68), PA (30.82), NO (30.46), PLDα (30.74)** | |
| Significantly increased | **ROS (44.4)** | | **cADPR (39.79), InsP3/6 (39.98), ROS (44.36), Ca^2+^_c_ (45.49)** | | **InsP3/6 (36.67), cADPR (36.69), ROS (44.37), Ca^2+^_c_ (45.44)** | | **InsP3/6 (36.75), cADPR (36.86), ROS (44.44), Ca^2+^_c_ (45.46)** | | **cADPR (40.69), InsP3/6 (40.82), ROS (44.39), Ca^2+^_c_ (45.78)** | |
| Decreased |  | | **PP2CA (5.11), H^+^ ATPase (6.59), AtRAC1 (10.89)** | | **PP2CA (3.15), H^+^ ATPase (4.05), AtRAC1 (6.64)** | | **H^+^ ATPase (3.91), AtRAC1 (6.71)** | | **PP2CA (5.52), H^+^ ATPase (7.24), AtRAC1 (12.1)** | |
| Significantly decreased |  | | **ABI1 (0.0), ABI2 (0.0)** | | **ABI1 (0.0), ABI2 (0.4)** | | **ABI1 (0.0), PP2CA (0.0), ABI2 (0.47)** | | **ABI1 (0.01), ABI2 (0.47)** | |
| Mean baseline percentage of closure and CPC | 0% | 0 | 30.28% | 13.05 | 18.79% | 8.04 | 18.45% | 7.91 | 32.46% | 14.11 |
| Standard deviation of the baseline percentage of closure and CPC | 0% | 0 | 0.38% | 0.15 | 0.55% | 0.23 | 0.63% | 0.27 | 0.8% | 0.35 |
