# Supplementary material for: Model-driven discovery of calcium-related protein-phosphatase inhibition in plant guard cell signaling
Source: PLoS Comput Biol. 2019 Oct 28;15(10):e1007429. doi: 10.1371/journal.pcbi.1007429 (PMC6837631; doi:10.1371/journal.pcbi.1007429)
Supplement: S1 Text — (DOCX) [file pcbi.1007429.s017.docx]

**Text S1. Boolean update rules and initial states used in the simulations of the reduced model**

***Justification for the states of the source nodes***

We reproduce for convenience the justifications provided in [1]. The nodes that are collapsed during network reduction (and thus are absent from the reduced network) are underlined. See Tables S1-S5 for the details of the reduction. In the reduced model CPK6 and CPK23 are merged into the node CPK6/23, whose state is the same as the shared state of CPK6 and CPK23, namely ON.

*Source nodes assumed to be ON*

**ABA**

ABA is a phytohormone, the primary signal for the model, which plays a vital positive regulatory role for stomatal closure by propagating the message through ABA receptors via signaling cascades, which in turn trigger the change in osmotic potential of stomatal guard cells in response to drought, leading to guard cell volume loss and stomatal closure. Once activated, this node is assumed to be in a sustained ON state to model the sustained presence of the signal [2,3].

**ABH1**

*ABH1* encodes an mRNA binding protein in Arabidopsis. The *abh1* (*AB*A *h*ypersensitive) loss-of-function mutant shows greater increases in cytosolic calcium and stomatal closure activation in response to ABA as compared to wild type. We assume that the wild type state of ABH1 is ON [4]. Because upstream regulators of ABH1 have not been identified, it is modeled as a source node.

**ARP Complex**

*arp2 (hsr3)* loss-of-function mutant exhibits reduced sensitivity to ABA and CaCl_2_ induced stomatal closure [5]. ARP2 is part of a protein complex involved in actin reorganization. In this particular case, the behavior of one subunit is assumed to describe the behavior of the protein complex. We assume that the wild type state of the ARP complex is ON.

**CPK6**

ABI1, ABI2, and PP2CA have been implicated as negative regulators of CPK6-mediated phosphorylation and thus activation of slow anion channels [6,7]. A recent report indicates that PP2Cs (which function as negative regulators in guard cell ABA signaling) do not downregulate CPK6 kinase activity directly [6]. Thus, we do not include ABI1, ABI2 and PP2CA as direct negative regulators of CPK6 in our model. CPK6 activity shows no or weak dependence on Ca^2+^_c_ [7]. As CPK6 is active at resting Ca^2+^_c_ levels, we assume that CPK6 is always on.

**CPK23**

ABI1 and ABI2 phosphatases have been implicated as negative regulators of CPK23-mediated activation of slow anion channels [8]. A recent report indicates that PP2Cs (which function as negative regulators in guard cell ABA signaling) do not downregulate CPK23 directly [9]. Thus, we do not include ABI1 and ABI2 as direct negative regulators of CPK23 in our model. CPK23 activity shows little or weak dependence on Ca^2+^ [7,8]. Hence, we assume that CPK23 is always on.

**DAGK**

DAGK is the enzyme that uses DAG as substrate for production of PA. We assume that DAGK is normally ON.

**ERA1**

ERA1 encodes a farnesyltransferase beta subunit. At low ABA concentrations, greater increases in cytosolic calcium and enhanced stomatal closure were observed in the *era1-2* knockout mutant in comparison to the wild type [10]. This mutant also shows hypersensitivity in ABA activation of slow anion channels [11]. We assume that the normal (wild type) state of ERA1 is ON.

**GAPC1/2**

GAPC1 and GAPC2 (glyceraldehyde-3-phosphate dehydrogenases) can interact with PLDδ [12]. We assume that these GAPCs are normally ON.

**GCR1**

GCR1 is a negative regulator of the sphingosine-1-phosphate (S1P) signal in guard cells and it can interact with GPA1. The *gcr1* loss-of-function mutant shows stronger ABA response than the wild type at moderate ABA concentrations, but interaction with GCR1 does not block G protein signaling [13]. We assume that unperturbed GCR1 is in the ON state.

**GTP**

GTP is the substrate for cGMP production. We assume that a sufficient amount of substrate is present, thus we assign this node the ON state.

**MRP5**

MRP5 encodes an ATP-binding cassette (ABC) transporter. The *mrp5* loss of function mutant shows partially impaired ABA-induced stomatal closure and assays of non-stretch-activated plasma membrane Ca^2+^ permeable channels show loss of activation in the *mrp5* mutant [14]. We assume that the undisrupted state of MRP5 is ON.

**NADPH**

NADPH is a coenzyme that is required for NO production. We assume that sufficient amount of co-enzyme is always present.

**Nitrite**

Nitrite is a substrate required for NO production. We assume that a sufficient amount of substrate is always present.

**NtSyp121**

Dominant-negative expression of the NtSyp121-Sp2 fragment inhibits opening of Ca^2+^ permeable channels [15] indicating that NtSyp121 is a positive regulator of CaIM. We assume that unperturbed NtSyp121 is in the ON state.

**PC**

PC is the substrate for PA production by both PLDα and PLDδ. We assume that a sufficient amount of substrate is always present.

**PtdInsP3, PtdInsP4**

LY294002 (inhibitor of PtdInsP3 biosynthesis) and wortmanin (inhibitor of PtdInsP4 biosynthesis) inhibit ABA-induced actin reorganization in guard cells, indicating that PtdInsP3 and PtdInsP4 are positive regulators of actin reorganization [16]. We assume that sufficient amounts of PtdInsP3 and PtdInsP4 are always present.

**RCN1**

RCN1 encodes the regulatory A subunit of a PP2A phosphatase. A T-DNA disruption mutant of *rcn1* showed hyposensitivity to ABA and lower ROS production [17]. We assume that undisrupted RCN1 is in the ON state. Because upstream regulators of RCN1 in guard cells have not been identified, it is modeled as a source node.

**SCAB1**

SCAB1 is an actin binding protein. The *scab1* loss-of-function mutant shows delayed stomatal closure and the mutant also shows slower actin reorganization in response to ABA in comparison to wild type plants [18]. We assume that undisrupted SCAB1 is in the ON state.

**Sph**

Sphingosine (Sph) is the substrate that is phosphorylated to produce S1P. SPHKs (1/2) use Sph for production of S1P. We assume that sufficient substrate is always present.

*Source nodes assumed to be OFF*

**GEF 1/4/10**

The *gef1gef4* double mutant (loss-of-function) and *gef1/4/10* triple mutant (loss-of-function) are hypersensitive in ABA-mediated stomatal responses. The *gef* double and triple mutants do not affect stomatal apertures in the absence of exogenous ABA [19-21]. We assume that the normal activity of GEF1/4/10 is below the threshold necessary to activate its downstream target ROP11, thus we assign it the OFF state. Assuming otherwise would not recapitulate the observed inhibitory effect of ABA on both ABI1 and ABI2 (see the rules for ABI1 and ABI2).

**SPP1**

Guard cells of the *atspp1* loss-of-function mutant show slightly enhanced stomatal closure over wild type in response to ABA. SPP1 encodes a long-chain base 1-phosphatase (LCBP) which has been implicated as a negative regulator of S1P accumulation in plants [22]. We assume that the normal activity of SPP1 is below the threshold required for efficient downregulation of S1P, and assign it the OFF state. Our model would capture the observed slight enhancement in stomatal closure in response to ABA in the *spp1* knockout [22] if we assumed that SPP1 is normally active (ON). However, this assumption, coupled with the knowledge that SPP1 is a negative regulator of S1P, would lead to the insufficiency of ABA to lead to S1P production, which contradicts observations [23,24].

***Boolean update rules of the reduced model with complete node names.***

Please see Tables S1-S5 for explanation of the node names. The update rules of the full model and their biological explanation can be found in the SI file S2 Text of [1]. The update rules of the reduced model are derived by following the reduction steps outlined in the Methods section of the main paper and detailed in Tables S2-S5. These rules do not use formatting such as superscripts and subscripts, so that they can be read into a Python code.

RCARs* = ABA

PEPC* = not ABA

PI3P5K->PtdIns(3,5)P2->V-PPase* = ABA

ABI1* = not PA[DAGK,PC] and (not RCARs or ROP11[GEF1/4/10]) and not RBOH[RCN1]{b}->ROS{a} and pHc

ABI2* = (not RCARs or ROP11[GEF1/4/10]) and not RBOH[RCN1]{b}->ROS{a}

HAB1* = not RCARs and not RBOH[RCN1]{b}->ROS{a}

PP2CA* = not RCARs and not RBOH[RCN1]{b}->ROS{a}

OST1[->PIP2;1]* = (not ABI1 and not HAB1) or (not PP2CA and not ABI2) or (not ABI1 and not ABI2) or (not HAB1 and not PP2CA) or (not HAB1 and not ABI2) or (not ABI1 and not PP2CA)

RBOH[RCN1]{b}->ROS{a}* = pHc and not ABI1 and OST1[->PIP2;1] and SPHK1/2->S1P[~SPP1,Sph]->GPA1[~GCR1] and PA[DAGK,PC]

GHR1* = not ABI2 and RBOH[RCN1]{b}->ROS{a}

NO[Nitrite]{a}* = NIA1/2

NIA1/2* = RBOH[RCN1]{b}->ROS{a}

NOGC1->cGMP[GTP]* = NO[Nitrite]{a}

8-nitro-cGMP->ADPRc->cADPR{a}* = NOGC1->cGMP[GTP] and RBOH[RCN1]{b}->ROS{a} and NO[Nitrite]{a}

CIS* = InsP3[->InsP6]{c} or 8-nitro-cGMP->ADPRc->cADPR{a}

CaIM[~ABH1,~ERA1,MRP5,NtSyp121]* = Actin_Reorganization[ARP_Complex,SCAB1]{b,c} or GHR1

Ca2+* = (CIS or CaIM[~ABH1,~ERA1,MRP5,NtSyp121]) and not Ca2+_ATPase

Ca2+_ATPase* = Ca2+

CPK3/21* = Ca2+ or CPK3/21

MPK9/12* = Ca2+ or MPK9/12

PLC* = Ca2+

DAG{c}* = PLC

InsP3[->InsP6]{c}* = PLC

PLDa* = SPHK1/2->S1P[~SPP1,Sph]->GPA1[~GCR1] and Ca2+

PLDdel[GAPC1/2]* = NO[Nitrite]{a} or RBOH[RCN1]{b}->ROS{a}

PA[DAGK,PC]* = PLDdel[GAPC1/2] or PLDa or DAG{c}

SPHK1/2->S1P[~SPP1,Sph]->GPA1[~GCR1]* = PA[DAGK,PC] or ABA

V-ATPase* = Ca2+

TCTP* = Ca2+

Microtubule_Depolymerization* = TCTP or Microtubule_Depolymerization

pHc* = ((OST1[->PIP2;1] and not ABI2 and not ABI1) or Ca2+) and Vacuolar_Acidification

H+_ATPase* = not pHc and not Ca2+ and not RBOH[RCN1]{b}->ROS{a}

AtRAC1* = not ABA or ABI1

Actin_Reorganization[ARP_Complex,SCAB1]{b,c}* = not AtRAC1

SLAC1* = (CPK6/23 or CPK3/21) and MPK9/12 and OST1[->PIP2;1] and GHR1 and not ABI1 and not PP2CA and not ABI2 and pHc

QUAC1* = OST1[->PIP2;1] and Ca2+

SLAH3* = CPK6/23 and CPK3/21 and not ABI1

AnionEM* = SLAC1 or QUAC1 and SLAH3

Malate* = PEPC and not ABA and not AnionEM

KEV* = Vacuolar_Acidification or Ca2+

Depolarization* = (AnionEM or Ca2+ or KEV) and (not H+_ATPase or not K+_efflux)

KOUT* = (not NO[Nitrite]{a} or not RBOH[RCN1]{b}->ROS{a} or pHc) and Depolarization

K+_efflux* = KEV and KOUT

H2O_Efflux* = AnionEM and OST1[->PIP2;1] and K+_efflux and not Malate

Closure* = Microtubule_Depolymerization and H2O_Efflux

Vacuolar_Acidification* = PI3P5K->PtdIns(3,5)P2->V-PPase or V-ATPase or Vacuolar_Acidification

***Initial states of nodes in the reduced model***

As in the non-reduced model, the initial state of the system aims to represent open stomata.

*Initial states of the nodes that are the same in the full and reduced models, reproduced from* [1]

Closure = H_2_O_Efflux = Microtubule Depolymerization = Ca^2+_^ATPase = RCARs = pH_c_ = Ca^2+^_c_ = K^+^ efflux = CIS = Vacuolar Acidification = PLDα = NIA1/2 = CPK3/21 = MPK9/12 = OFF

Malate = H^+^ ATPase = ABI1 = ABI2 = HAB1 = PP2CA = CPK6/23 = ON

Nodes initiated in a randomly selected state: AnionEM, AtRAC1, Depolarization, GHR1, KEV, KOUT, PEPC, PLC, QUAC1, SLAC1, SLAH3, TCTP, V-ATPase

*Justification for choice of initial states of merged nodes in the reduced network*

For each node listed in Tables S2 and S3, where a source node is reduced, we use the initial state of the target node as the initial state of the merged node. This is because the state of the source node is already incorporated in the reduction process. The only exception is ROP11, whose sole regulator is GEF1/4/10, with the update function ROP11*=GEF1/4/10. GEF1/4/10 was OFF in the full model. ROP11 was randomly initiated, but adopted the OFF state at the first time it was updated. To account for ROP11’s dependence on GEF1/4/10, we initialize the merged node ROP11[GEF1/4/10] as OFF.

The full list of initial conditions of merged nodes in Tables S2 and S3 is

NO = CaIM = PA = ROP11 = Actin Reorganization = OFF

Nodes initiated randomly: PLDδ, DAG

For nodes listed in Table S4, the choice of initial state is made on a case-to-case basis and each case is listed below:

**cADPR:** This node represents the merger of three nodes, 8-nitro-cGMP→ADPRc→cADPR. The rules for each of the nodes are: ADPRc*= 8-nitro-cGMP; cADPR*= ADPRc. The initial state of all of these nodes in the full model is random, thus the merged node cADPR is initialized as random.

**S1P:** This node represents the merger of three nodes, SPHK1/2→S1P→GPA1. The rules are: GPA1*= S1P; S1P*= SPHK1/2. The initial states of the three nodes in the full model are: GPA1 = S1P = OFF; SPHK1/2 initiated randomly. In the reduced model, the node SPHK1/2→S1P→GPA1 (proxy name S1P) was initialized as OFF, since this is the choice that is closest to an initial state of open stomata.

**cGMP:** This node represents the merger of two nodes, NOGC1→cGMP. The rule is: cGMP*= NOGC1. The initial states of the two nodes in the full model are: cGMP = NOGC1 = OFF, thus the merged node is initialized as OFF.

**V-PPase:** This node represents the merger of three nodes, PI3P5K→PtdIns(3,5)P2→V-PPase. The rules are: V-PPase*= PtIns(3,5)P2; PtdIns(3,5)P2*= PI3P5K. The initial states of the three nodes in the full model are randomly chosen, thus the merged node is also initialized randomly.

**ROS**: This node represents the merger of two nodes: RBOH→ROS. The rule is: ROS*= RBOH. The initial states in the full model are: RBOH = ROS = OFF, thus the merged node is also initialized as OFF.

For the two nodes in Table S5 that are retained in the reduced model, namely, InsP3/6 and OST1, the initial state of the merged node is assumed to be the same as the initial state of the regulator node. This is because, in the next time step, the target node state will be equal to the initial state of the regulator node, hence it is logical to confer on both nodes (i.e. on the merged node) the initial state of the regulator node. Accordingly, InsP3/6 and OST1 are initially OFF.

The full model initializes 28 nodes (of the 80 nodes other than ABA) as OFF, 25 as ON and 27 randomly, while the reduced model initializes 24 nodes (of the 48 nodes other than ABA) as OFF, 7 as ON and 17 randomly. These 17 nodes are AnionEM, AtRAC1, Depolarization, GHR1, KEV, KOUT, PEPC, PLC, QUAC1, SLAC1, SLAH3, TCTP, V-ATPase, PLDδ, DAG, cADPR, V-PPase.

1. Albert R, Acharya BR, Jeon BW, Zanudo JGT, Zhu M, Osman K, et al. A new discrete dynamic model of ABA-induced stomatal closure predicts key feedback loops. PLoS Biol. 2017;15(9):e2003451.

2. Ma Y, Szostkiewicz I, Korte A, Moes D, Yang Y, Christmann A, et al. Regulators of PP2C phosphatase activity function as abscisic acid sensors. Science. 2009;324(5930):1064-8.

3. Park SY, Fung P, Nishimura N, Jensen DR, Fujii H, Zhao Y, et al. Abscisic acid inhibits type 2C protein phosphatases via the PYR/PYL family of START proteins. Science. 2009;324(5930):1068-71.

4. Hugouvieux V, Kwak JM, Schroeder JI. An mRNA cap binding protein, ABH1, modulates early abscisic acid signal transduction in Arabidopsis. Cell. 2001;106(4):477-87.

5. Jiang K, Sorefan K, Deeks MJ, Bevan MW, Hussey PJ, Hetherington AM. The ARP2/3 complex mediates guard cell actin reorganization and stomatal movement in Arabidopsis. Plant Cell. 2012;24(5):2031-40.

6. Brandt B, Brodsky DE, Xue S, Negi J, Iba K, Kangasjarvi J, et al. Reconstitution of abscisic acid activation of SLAC1 anion channel by CPK6 and OST1 kinases and branched ABI1 PP2C phosphatase action. Proc Natl Acad Sci U S A. 2012;109(26):10593-8.

7. Scherzer S, Maierhofer T, Al-Rasheid KA, Geiger D, Hedrich R. Multiple calcium-dependent kinases modulate ABA-activated guard cell anion channels. Mol Plant. 2012;5(6):1409-12.

8. Geiger D, Scherzer S, Mumm P, Marten I, Ache P, Matschi S, et al. Guard cell anion channel SLAC1 is regulated by CDPK protein kinases with distinct Ca2+ affinities. Proc Natl Acad Sci U S A. 2010;107(17):8023-8.

9. Brandt B, Munemasa S, Wang C, Nguyen D, Yong T, Yang PG, et al. Calcium specificity signaling mechanisms in abscisic acid signal transduction in Arabidopsis guard cells. eLife. 2015;4.

10. Allen GJ, Murata Y, Chu SP, Nafisi M, Schroeder JI. Hypersensitivity of abscisic acid-induced cytosolic calcium increases in the Arabidopsis farnesyltransferase mutant era1-2. Plant Cell. 2002;14(7):1649-62.

11. Pei ZM, Ghassemian M, Kwak CM, McCourt P, Schroeder JI. Role of farnesyltransferase in ABA regulation of guard cell anion channels and plant water loss. Science. 1998;282(5387):287-90.

12. Guo L, Devaiah SP, Narasimhan R, Pan X, Zhang Y, Zhang W, et al. Cytosolic glyceraldehyde-3-phosphate dehydrogenases interact with phospholipase Ddelta to transduce hydrogen peroxide signals in the Arabidopsis response to stress. Plant Cell. 2012;24(5):2200-12.

13. Pandey S, Assmann SM. The Arabidopsis putative G protein-coupled receptor GCR1 interacts with the G protein alpha subunit GPA1 and regulates abscisic acid signaling. Plant Cell. 2004;16(6):1616-32.

14. Suh SJ, Wang YF, Frelet A, Leonhardt N, Klein M, Forestier C, et al. The ATP binding cassette transporter AtMRP5 modulates anion and calcium channel activities in Arabidopsis guard cells. J Biol Chem. 2007;282(3):1916-24.

15. Sokolovski S, Hills A, Gay RA, Blatt MR. Functional interaction of the SNARE protein NtSyp121 in Ca2+ channel gating, Ca2+ transients and ABA signalling of stomatal guard cells. Mol Plant. 2008;1(2):347-58.

16. Choi Y, Lee Y, Jeon BW, Staiger CJ, Lee Y. Phosphatidylinositol 3- and 4-phosphate modulate actin filament reorganization in guard cells of day flower. Plant Cell Environ. 2008;31(3):366-77.

17. Saito N, Munemasa S, Nakamura Y, Shimoishi Y, Mori IC, Murata Y. Roles of RCN1, regulatory A subunit of protein phosphatase 2A, in methyl jasmonate signaling and signal crosstalk between methyl jasmonate and abscisic acid. Plant Cell Physiol. 2008;49(9):1396-401.

18. Zhao Y, Zhao S, Mao T, Qu X, Cao W, Zhang L, et al. The plant-specific actin binding protein SCAB1 stabilizes actin filaments and regulates stomatal movement in Arabidopsis. Plant Cell. 2011;23(6):2314-30.

19. Li Z, Gao X, Chinnusamy V, Bressan R, Wang ZX, Zhu JK, et al. ROP11 GTPase negatively regulates ABA signaling by protecting ABI1 phosphatase activity from inhibition by the ABA receptor RCAR1/PYL9 in Arabidopsis. J Integr Plant Biol. 2012;54(3):180-8.

20. Li Z, Liu D. ROPGEF1 and ROPGEF4 are functional regulators of ROP11 GTPase in ABA-mediated stomatal closure in Arabidopsis. FEBS Lett. 2012;586(9):1253-8.

21. Yu F, Qian L, Nibau C, Duan Q, Kita D, Levasseur K, et al. FERONIA receptor kinase pathway suppresses abscisic acid signaling in Arabidopsis by activating ABI2 phosphatase. Proc Natl Acad Sci U S A. 2012;109(36):14693-8.

22. Nakagawa N, Kato M, Takahashi Y, Shimazaki K, Tamura K, Tokuji Y, et al. Degradation of long-chain base 1-phosphate (LCBP) in Arabidopsis: functional characterization of LCBP phosphatase involved in the dehydration stress response. J Plant Res. 2012;125(3):439-49.

23. Coursol S, Fan LM, Le Stunff H, Spiegel S, Gilroy S, Assmann SM. Sphingolipid signalling in Arabidopsis guard cells involves heterotrimeric G proteins. Nature. 2003;423(6940):651-4.

24. Worrall D, Liang YK, Alvarez S, Holroyd GH, Spiegel S, Panagopulos M, et al. Involvement of sphingosine kinase in plant cell signalling. Plant J. 2008;56(1):64-72.
