# Supplementary material for: Model-driven discovery of calcium-related protein-phosphatase inhibition in plant guard cell signaling
Source: PLoS Comput Biol. 2019 Oct 28;15(10):e1007429. doi: 10.1371/journal.pcbi.1007429 (PMC6837631; doi:10.1371/journal.pcbi.1007429)
Supplement: S2 Text — (DOCX) [file pcbi.1007429.s018.docx]

**Text S2. Average ON/OFF time periods of nodes driven by a negative feedback loop.**

Tables S7 and S13 list the nodes that oscillate in the attractor associated with closure in the presence or absence of ABA. These nodes fall into three categories. The first category contains Ca^2+^_c_ and Ca^2+^ ATPase, which both have average ON and OFF periods of 1.33 time steps. The second category contains PLC, PLDα, QUAC1, TCTP and V-ATPase, which have average ON/OFF periods of 1.77 time steps. All the nodes in the second category are directly regulated by Ca^2+^_c_. The third category contains DAG and InsP3/6, which have average ON/OFF periods of 2.2 time steps – both of these nodes are at a distance of 2 from Ca^2+^_c_. Importantly, in both attractors associated with closure (Table S7, Table S13), CaIM and CIS are stabilized in the ON state. In this section we study a small toy network to understand these oscillations.

Consider a network composed of two nodes that form a negative feedback loop. The corresponding regulatory functions are: A* = not B; B* = A (see Figure S2A). Here A corresponds to Ca^2+^_c_ in the case where the condition “CaIM or CIS” is permanently satisfied, and B corresponds to Ca^2+^ ATPase.


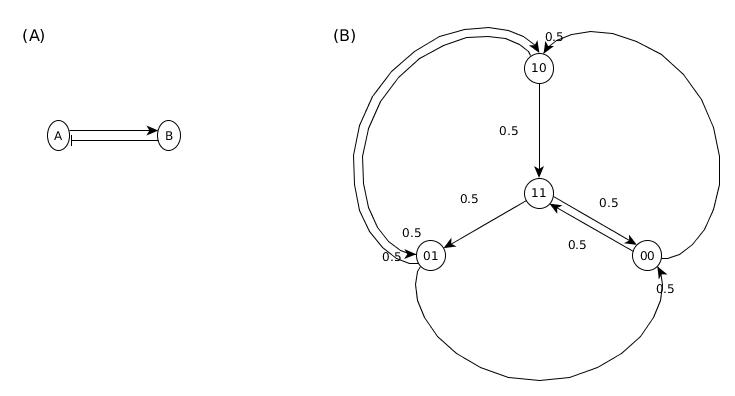


Fig S2: **Analysis of a toy network representing the oscillation of Ca^2+^_c_ in the negative feedback loop formed by Ca^2+^_c_ and Ca^2+^ ATPase.** (A) Network diagram, where node A represents Ca^2+^_c_ and B represents Ca^2+^ ATPase. (B) State transition graph for the two-node toy network in the random order asynchronous update scheme (the update scheme used in our analysis of the ABA network, see Methods). Each state is represented in the A,B order of nodes*.*

The possible trajectories of the network can be summarized in a state transition graph, whose nodes are the possible states of the system (the four states that range from A=B=0 to A=B=1), and whose directed edges indicate the allowed transitions among states. Each transition involves update of node A and B, in either the order A,B or B,A. For example, consider that the system is in the state 00, where the first number stands for the state of A and the second for the state of B. Following the order A,B, we first update A, yielding A=1, and then update B, yielding B=1; thus the state becomes 11. Conversely, if following the update order B,A, then we first update B which stays 0, and then update A yielding A=1, so the resultant state is 10. As both update orders have the same likelihood, the state transitions from 00 to 11 and to 10 have the same probability, 0.5. The full state transition graph is shown in Figure S2B, where the edges are marked with the probability of state transition.

The information encapsulated in the state transition graph can be used to determine the likelihood of ON and OFF periods of various durations. Let us start in the ON (1) state of A and assume that it was previously OFF (0). The ON state of node A implies that the system is either in state 11 or in state 10. If the system is in state 11, the possible next states are 01 or 00, both of which correspond to the OFF state of node A. Hence, starting in state 11, the duration of the ON period of A is 1 time step. If the system is in state 10, the possible next state of 01 (reached with probability 0.5) corresponds to the OFF state of node A, which implies an ON sequence of length 1. The other possible next state is 11 (also reached with probability 0.5), which implies an ON duration of 2 time steps. Hence, the average ON period for A is 0.5*1 + 0.5*2 = 1.5. According to our assumption, there must have been a transition from A=OFF to A=ON to get to either state 11 or 10. There are three such transitions: 00 → 10; 01 → 10; 00 → 11. Of these equally probable transitions, two lead to the 10 state and one leads to the 11 state. Hence, given that A is ON, the system is in state 10 with probability 0.67 and is in state 11 with probability 0.33. Hence, the overall average ON period le is 0.33*1 + 0.67*1.5 = 1.33 time steps. The average OFF period can be calculated with a similar argument and has the same value.

Let us now add a successor of node A to capture PLC (see Figure S3A). The updated Boolean rules are: A* = not B; B* = A; C* = A. This gives the new state transition diagram as shown in Figure S3B.


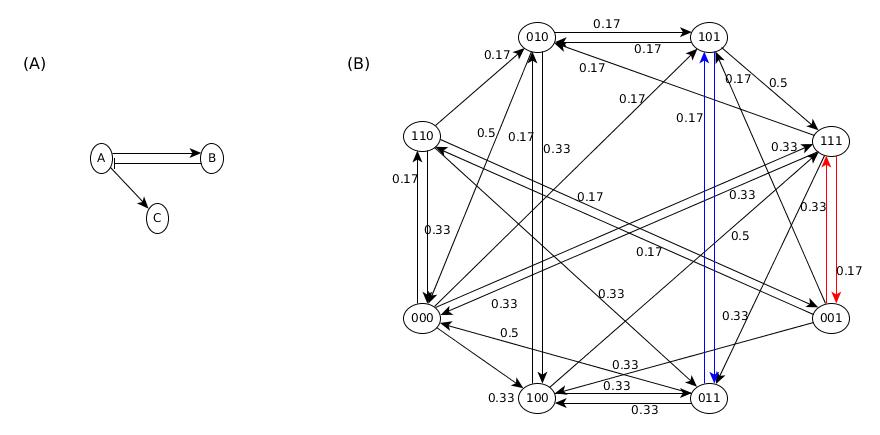


Fig S3**: A toy network representing the negative feedback loop along with a third node directly regulated by the oscillating node.** (A) Toy network with three nodes, two are part of a negative feedback loop and the third is at distance 1 from the oscillating node A. (B) State transition graph for the three-node toy network for random order asynchronous update. The states are represented in the ABC order of nodes.

When we similarly analyze the ON period of node A in the state transition graph of the three-node network, we obtain the same results. To understand the average ON period of node C, we note that that there are two two-state cycles of the three-node network wherein C=ON: the cycle between the states 001 and 111 (highlighted in red in Fig. S3B) and the cycle between 101 and 011 (highlighted in blue in Fig S3B). The system can spend a variable amount of time going back and forth among the states of the cycle, but all the while C stays ON. Hence, the average ON period for node C is larger than the average ON period of node A. The further away a node is from an oscillating node, the more cycles there will be in which the node state stays the same will be in the state transition graph, hence leading to longer ON and OFF periods.
