# Supplementary material for: Model-driven discovery of calcium-related protein-phosphatase inhibition in plant guard cell signaling
Source: PLoS Comput Biol. 2019 Oct 28;15(10):e1007429. doi: 10.1371/journal.pcbi.1007429 (PMC6837631; doi:10.1371/journal.pcbi.1007429)
Supplement: S3 Text — (DOCX) [file pcbi.1007429.s019.docx]

**Text S3. Justification of the hypothesized inhibitory edges(s) and analysis of alternative possibilities**

Our original model successfully recapitulated 13 instances of experimentally observed stomatal behavior upon constitutive activation of an internal node in the absence of ABA and did not recapitulate 9 experimental observations of closure [1]. We made two key observations in our previous analysis: (i) the 9 nodes whose constitutive activation can lead to closure according to experiments but not in the model participate in paths that converge to Ca^2+^ release from stores and (ii) even transient inactivation of the PP2C phosphatases is able to resolve 8 discrepancies. These observations suggest that there is a logic implication of sufficiency between the 9 nodes that were experimentally observed to lead to closure and the inactivation (OFF state) of the PP2Cs. As the original model did not exhibit such logic implication, the model needed to be augmented in such a way that this logic implication emerges. The fact that 3 of the 9 nodes, namely 8-nitro-cGMP, cADPR, and InsP3 are logically sufficient to induce an increase in cytosolic Ca^2+^, and Ca^2+^_c_ itself is in this group, suggests that adding a sufficient inhibitory relationship from Ca^2+^_c_ to the PP2Cs, which ensures the inactivation of PP2Cs every time there is activation of Ca^2+^_c_, may induce the desired logic implication. Indeed, we verified by simulations that the original model augmented with four inhibitory edges (one to each of ABI1, ABI2, HAB1, PP2CA) starting from Ca^2+^_c_ resolved 8 of the 9 discrepancies between model and experiment.

## In our current analysis we search for more parsimonious additions to the model. We first consider subsets of these four inhibitory edges, starting with individual edges from Ca^2+^_c_ to each of ABI1, ABI2, HAB1 or PP2CA. The results of this analysis are shown in the Results section “Model Predictions Concerning Ca^2+^_c_ Inhibiting One or More PP2Cs”. In a nutshell, we find all that the augmented models (with the exception of the model where Ca^2+^_c_ directly inhibits ABI1) resolve the previous discrepancies, and additionally they have a nonzero baseline probability of closure.

## Since our experimental results indicate that inhibition of PP2C activity by Ca^2+^_c_ does occur in vivo, but that the mechanism of inhibition is indirect, we then consider parsimonious ways of having an indirect sufficient inhibitory relationship between Ca^2+^_c_ and either of ABI2, HAB1 or PP2CA. There already is an indirect sufficient inhibitory relationship between Ca^2+^_c_ and ABI1, namely Ca^2+^_c_ → PA –● ABI1, wherein Ca^2+^_c_ induces PA production by upregulating PLDα and PLC (see Fig 6A), and PA directly binds to and inhibits ABI1. This pathway serves as the inspiration for assuming that PA may be inhibiting additional PP2C(s). As presented in the Results section “PP2C Inhibition Mediated by PA Gives Similar Results”, there is a strong matching between the results of each model version that posits direct inhibition of one or more PP2C by Ca^2+^_c_ with the model version where the same PP2C(s) are instead inhibited by PA.

To complete our analysis, we evaluate whether any alternative edge starting from Ca^2+^_c_ would also be able to solve the cases of discrepancy between model and experiments. Since obtaining closure in the absence of ABA will always need the activation of the stable motif associated with closure in the absence of ABA (Fig 4), any such edge would need to make Ca^2+^_c_ a driver of the stable motif. The stable motif has 8 nodes, of which PA is already regulated by Ca^2+^_c_ and ABI1 and ABI2 were already considered in previous analysis. We separately consider each of the remaining 5 nodes, namely PLDδ, S1P, ROS, PA, OST1, and augment their regulatory functions with a sufficient activating edge from Ca^2+^_c._ (As these nodes are active in the stable motif associated with closure, their negative regulation by Ca^2+^_c_ would not have a positive influence on the stable motif.) Ca^2+^_c_ becomes a driver of the stable motif (or, equivalently, external Ca^2+^ is able to lead to closure) in only one of the cases: Ca^2+^_c_ activation of ROS. This is consistent with our result that ROS is a driver of the stable motif associated with closure in the absence of ABA. In other words, in this scenario, activation of the Ca^2+^_c_ node leads to activation of the ROS node, which stabilizes the motif. ROS production has multiple documented necessary regulators (pH_c_, OST1, PI3K, RCN1); loss of any of these was observed to impair ROS production in response to ABA (see Text S2 of [1]). ROS production is also lost in an *abi1* dominant mutant (which confers ABI1 constitutive activity). Hence, it is unlikely that increase in Ca^2+^_c_ would be sufficient for ROS production (logic sufficiency means that increase in Ca^2+^_c_ would be able to induce ROS production even if pH_c_, OST1, PI3K, RCN1 are OFF and ABI1 is ON). So, we also test a weaker condition that does not contradict the biological information about ROS, namely that Ca^2+^_c_ is necessary but not sufficient for ROS production. This case does not recapitulate the experimental result of stomatal closure induced by Ca^2+^_c_.

The analysis described above supports our conclusion that the target of the new edge must be a PP2C. Yet, the existence of alternative or additional putative mediators of an inhibitory effect from Ca^2+^_c_ to a PP2C cannot be ruled out. Denoting the putative mediator by X and considering as an example that the target PP2C is ABI2, there are two possible logic paths through which Ca^2+^_c_ yields the inactivation of ABI2: (i) Ca^2+^_c_ is a sufficient activator of X and X is a sufficient inhibitor of ABI2, or (ii) Ca^2+^_c_ is a sufficient inhibitor of X, which is a required activator of ABI2. There are no candidates for node X in the original or the reduced model other than PA and ROS. Future work may identify other relevant nodes that may play this role.

1. Albert R, Acharya BR, Jeon BW, Zanudo JGT, Zhu M, Osman K, et al. A new discrete dynamic model of ABA-induced stomatal closure predicts key feedback loops. PLoS Biol. 2017;15(9):e2003451.
