# Supplementary material for: Model-driven discovery of calcium-related protein-phosphatase inhibition in plant guard cell signaling
Source: PLoS Comput Biol. 2019 Oct 28;15(10):e1007429. doi: 10.1371/journal.pcbi.1007429 (PMC6837631; doi:10.1371/journal.pcbi.1007429)
Supplement: S4 Text — (DOCX) [file pcbi.1007429.s020.docx]

**Text S4. Exploration of the effect of Ca^2+^_c_ patterns on stomatal closure in the model version where Ca^2+^_c_ inhibits ABI2**

Due to the negative feedback loop mediated by the node Ca^2+^ ATPase, the state of the Ca^2+^_c_ node cannot stabilize at 1. Instead, it has two possible behaviors: stabilizing at 0, in which case closure is not possible, or Ca^2+^_c_ oscillations with average ON/OFF period of 1.33 time steps (see Text S2). These possible behaviors are also illustrated in Tables S7 and S13. We investigated what type of Ca^2+^_c_ patterns can act as internal signals (can drive closure) in the absence of ABA in the model version where Ca^2+^_c_ inhibits ABI2.

For this analysis, we replaced the regulatory function of Ca^2+^_c_ with synthetically generated patterns of activation and monitored the rest of the system. We will use the term pulse to denote the sustained ON state of a node for a given time period (e.g. two time steps), after which the node stays fixed in the OFF state. We will use the term spike to denote the ON state of a node for just one time step, after which the node goes to the OFF state.

We first studied the effect of a single synthetically generated Ca^2+^_c_ pulse on stomatal closure. The first ON state of Ca^2+^_c_ in this pulse is at t=1. We found that the steady-state (final) percentage of closure increases as we increase the length of this pulse. When the pulse length is 5 time steps or more, we obtain 100% of closure (see Figure S4). In order to explain this effect, we investigate the nodes in the stable motif shown in Figure 4B. A first observation is that the ON state of Ca^2+^ for just one time step is enough to stabilize the Vacuolar Acidification motif in the ON state, which leads to Ca^2+^_c_ becoming an external driver node of this stable motif. We find that a Ca^2+^_c_ pulse of four or more time steps ensures the simultaneous activation of PA and pH_c_ and inactivation of ABI2 such that they collectively form a three-node driver of the stable motif. ABI2 becomes inactive within one time step after the increase of Ca^2+^_c_, pH_c_ increases within two time steps, and PA increases, through the Ca^2+^ → PLC → DAG → PA path, within three time steps. We observed that once the motif is stabilized the ON state of the Closure node can be maintained even if Ca^2+^_c_ later stabilizes in the OFF state.


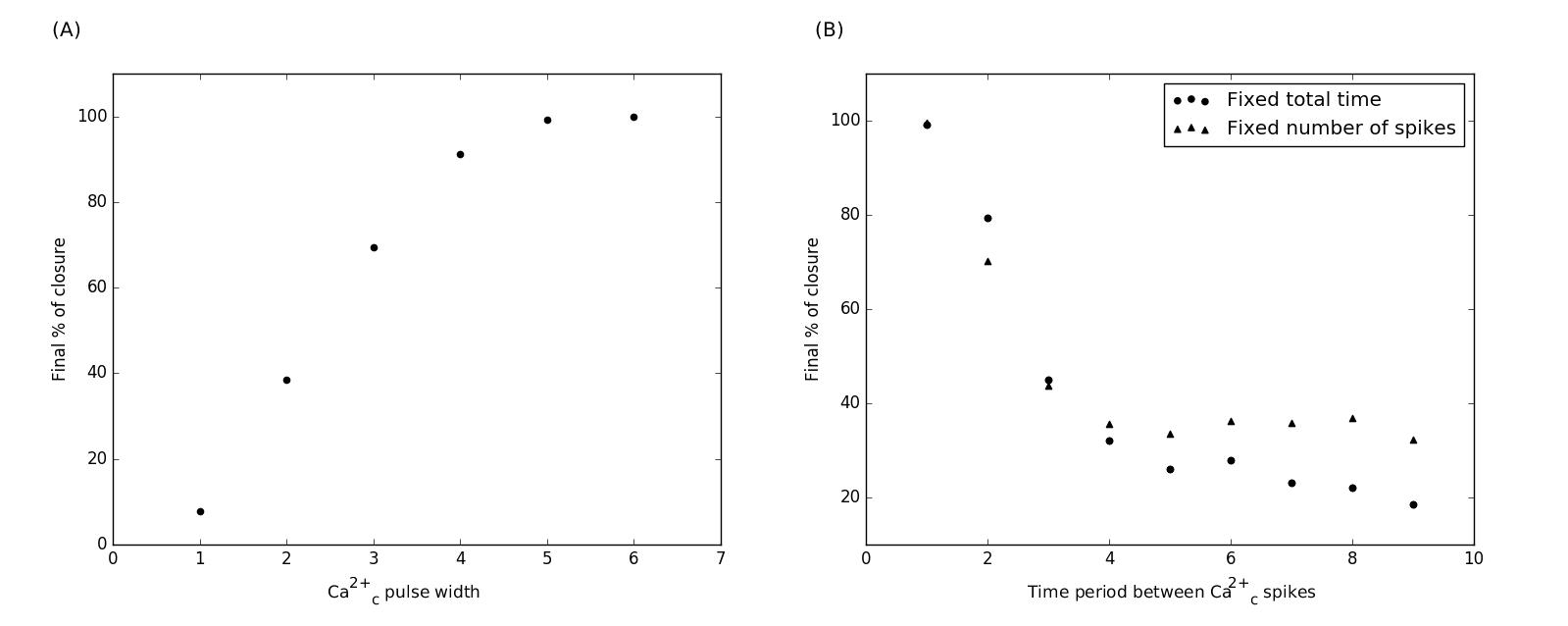


Fig S4: **Dependence of the final percentage of closure on parameters of various Ca^2+^_c_ patterns in the model version where Ca^2+^_c_ directly inhibits ABI2.** (A). Dependence of the final percentage of closure on the length of an imposed Ca^2+^_c_ pulse for the model version where Ca^2+^_c_ inhibits ABI2. We performed 500 simulations, each with a duration of 50 time steps. The final percentage of closure is calculated as the maximum of the percentage of closure in the last 5 time steps. The length of the Ca^2+^_c_ pulse means the time period for which Ca^2^_c_^+^ is fixed in the ON state and after which it is fixed in the OFF state. (B). The final percentage of closure as a function of the time period between two consecutive Ca^2+^_c_ spikes for the model version where Ca^2+^_c_ inhibits ABI2. The circles indicate the cases where the total time was fixed at 50 time steps, thus there are 17 spikes if the time between spikes is 2 time steps and five spikes if the time between spikes is 9 time steps. The triangles indicate the cases where the duration of the simulation was increased to accommodate a total number of 11 spikes. The final percentage of closure is calculated as the maximum of the percentage of closure in the last 5 time steps. Each of the data points corresponds to a set of 500 simulations.

Since sustained high Ca^2+^_c_ concentration is damaging to the cell, we next considered a more biologically appropriate pattern: multiple equally spaced Ca^2+^_c_ spikes (e.g. a single timestep of ON state followed by two time steps of OFF state, then again a single timestep of ON state, followed by two timesteps of OFF state, and so on) over 50 time steps (the duration of all the simulations). We found a significant percentage of closure if the time between spikes was relatively small (see Figure S4B). Specifically, if the time between consecutive Ca^2+^_c_ spikes equals one time step, the simulations result in ~100% stomatal closure. We observed that the percentage of closure reduces with increased spacing of spikes, reaching below baseline percentage (obtained in the absence of any signal) when there are only five spikes separated by 9 time steps. As an alternative, we also implemented a variable simulation duration in such a way to accommodate a fixed number of spikes (11) at different time delays. The results were consistent: high percentage of closure for short spacings and decreasing with longer spacing between spikes (see circular and triangular symbols on Figure S4B). These results indicate that a high frequency as well as a significant number of Ca^2+^_c_ spikes are necessary for consistent induction of closure. This conclusion agrees with the experimental observation that increasing numbers of membrane-hyperpolarization-induced Ca^2+^_c_ transients (ranging from one to nine) led to decreased stomatal apertures (more pronounced closure) [1]. When varying the durations of the periods of high and low Ca^2+^_c_ concentrations, the observed decrease in aperture was most pronounced when the two durations were equal [1]. This is consistent with the most effective simulated Ca^2+^_c_ pattern of one time step ON, one time step OFF. Future in depth analysis may uncover all the time scales relevant to the effectiveness of the Ca^2+^_c_ pattern and hint toward a theoretical basis for encoding the relationship between Ca^2+^_c_ oscillation parameters and the percentage of closure.

The ability of repeated Ca^2+^ spikes to lead to closure is also due to the stabilization of the stable motif of Figure 4B. A spike of Ca^2+^_c_ leads to a temporary inactivation of ABI2 and activation of pH_c_, and has a moderate chance to lead to the ON state of PA via the Ca^2+^ → PLC → DAG → PA sufficient path. With certain update orders in the asynchronous update, the feedback loops of the stable motif (e.g. PA → ROS → PLDα→ PA and ABI2–●pH_c_→ROS–●ABI2) enable pulses of PA and pH_c_ activity as well as ABI2 inactivity. Repeated instances of Ca^2+^_c_ spike-induced pulses can yield a co-incidence of PA = pH_c_ = 1, ABI2 =0, which form a driver of the stable motif.

Hence, we establish that Ca^2+^_c_ patterns can also be drivers of the stable motif in Figure 4A in a similar sense as a stabilized ON state of Ca^2+^_c_. To complete this analysis, we used the output of the two-node network described in Text S2 (namely, irregular oscillations with average ON and OFF periods of 1.33 time steps) as an input Ca^2+^_c_ pattern and observed the state of the Closure node. We ran a total of 1000 simulations covering 10 different output patterns of the two-node feedback network. We obtained a final probability of closure of 99.3%. This near-certain closure probability suggests that the near-parity of the ON and OFF periods is the key feature of a closure-driving Ca^2+^_c_ pattern.

1. Allen GJ, Chu SP, Harrington CL, Schumacher K, Hoffmann T, Tang YY, et al. A defined range of guard cell calcium oscillation parameters encodes stomatal movements. Nature. 2001;411(6841):1053.
