# Supplementary material for: Model-driven discovery of calcium-related protein-phosphatase inhibition in plant guard cell signaling
Source: PLoS Comput Biol. 2019 Oct 28;15(10):e1007429. doi: 10.1371/journal.pcbi.1007429 (PMC6837631; doi:10.1371/journal.pcbi.1007429)
Supplement: S5 Text — (DOCX) [file pcbi.1007429.s021.docx]

**Text S5. Theoretical analysis of the ABA-independent closure-inducing stable motif in case of Ca^2+^_c_ - initiated inhibition of the PP2Cs.**

Stable motifs are generalized positive feedback loops that maintain a fixed state of their constituent nodes once stabilized. These motifs can be identified by finding strongly connected (feedback-rich) components (SCC) in an expanded network that expresses the regulatory function of each node [1]. The stable motifs of a Boolean network can be used to determine the various attractors (e.g. steady states) of the system. This is because stabilization of a stable motif (or successive stabilization of a group of stable motifs) drives the entire network to a particular attractor. In the ABA signaling network [2], there exists a stable motif that is associated with closure in the absence of ABA. As shown in Figure 4A and Figure S1C, this stable motif involves the activation of PLDδ, PA, SPHK1/2, S1P, RBOH, ROS, OST1 and pH_c_ and the inhibition of ABI1 and ABI2.

Maheshwari et. al. identified different causal logic designations that can be assigned to different edges depending on how the state of a node propagates through the edge to the successor node [3]. The logic designations identified are “sufficient” and “necessary”. An edge is sufficient if the ON state of the regulator is independently enough to stabilize the successor node to the ON state, while an edge is necessary if the OFF state of the regulator is enough to stabilize the successor node to the OFF state. This logic framework allows an alternative method of identification of stable motifs by finding cyclic paths or subgraphs associated with a particular causal logic (e.g., a cycle of sufficient edges). In these motifs, there are certain nodes (referred to as “driver nodes”) which when stabilized to their corresponding fixed state in the stable motif, can stabilize all the nodes of the stable motif. Driver nodes can also exist outside of the motif; when reaching an associated state these nodes can stabilize the motif – these are referred to as “external driver nodes”. A combination of nodes can also drive the motif e.g. if two nodes in the motif when fixed to their corresponding stable states can drive the motif to stability, we refer to this two-node combination as a “two-node driver set”. Similarly, we define a “three-node driver set” where a combination of three nodes can stabilize the motif.

Here we describe the stable motif associated with closure in the absence of ABA, and its drivers, in the original reduced model (shown in Figure 4A), and in the model versions that assume that Ca^2+^_c_ inhibits ABI1 (Figure S5A), Ca^2+^_c_ inhibits ABI2 (Figure 4B), Ca^2+^_c_ inhibits HAB1 or PP2CA (Figure S5B), or PA inhibits ABI2 (Figure 4C), respectively. All these incarnations of the stable motif contain the same nodes and share the 15 edges of the stable motif of the original reduced model (of Figure 4A). The different model versions contain different versions of an added, inhibitory edge. All versions of the stable motif depend on the prior establishment of vacuolar acidification.

Figure 4A shows the stable motif that when stabilized, drives the network to the Closure=ON attractor in the absence of ABA. The stable motif states are as follows: PLDδ=ON, PA=ON, S1P=ON, ROS=ON, OST1=ON, pH_c_=ON, ABI1=OFF, ABI2=OFF. ROS (node in orange color in Figure 4A) is a single-node driver of this motif. If ROS is fixed to the ON state, it activates PLDδ, which catalyzes PA production, which in turn leads to S1P production. ROS also inhibits ABI1 and ABI2, which in turn allows OST1 to activate. ROS indirectly activates both CaIM and CIS (see Figure 7A in the main text), thus induces Ca^2+^_c_ oscillations, which induce vacuolar acidification through the activation of the V-ATPase. The activity of OST1, coupled with the inactivity of ABI1 and ABI2 and the presence of vacuolar acidification leads to pH_c_ increase (the ON state of the pH_c_ node). In summary, sustained presence of ROS can stabilize the entire motif, hence eventually stabilizing the network to the Closure = ON attractor. PLDδ, pH_c_ and ABI2 make up a three-node driver set, that is, if PLDδ is fixed to the ON state, pH_c_ is fixed to the ON state, and ABI2 is fixed to the OFF state, then the entire motif stabilizes. Indeed, PLDδ activation leads to PA production, which leads to activation of S1P and inhibits ABI1. Inhibition of ABI1 and ABI2 activates OST1. The activity of PA, S1P, OST1, pH_c_, coupled with inactivity of ABI1 is sufficient for ROS production, completing the stable motif. Note that not every combination of three nodes can stabilize the entire motif. For example, the combination PA= pH_c_=ON, ABI1=OFF cannot. Although this combination can activate S1P, it is not sufficient to inhibit ABI2 or to activate ROS.


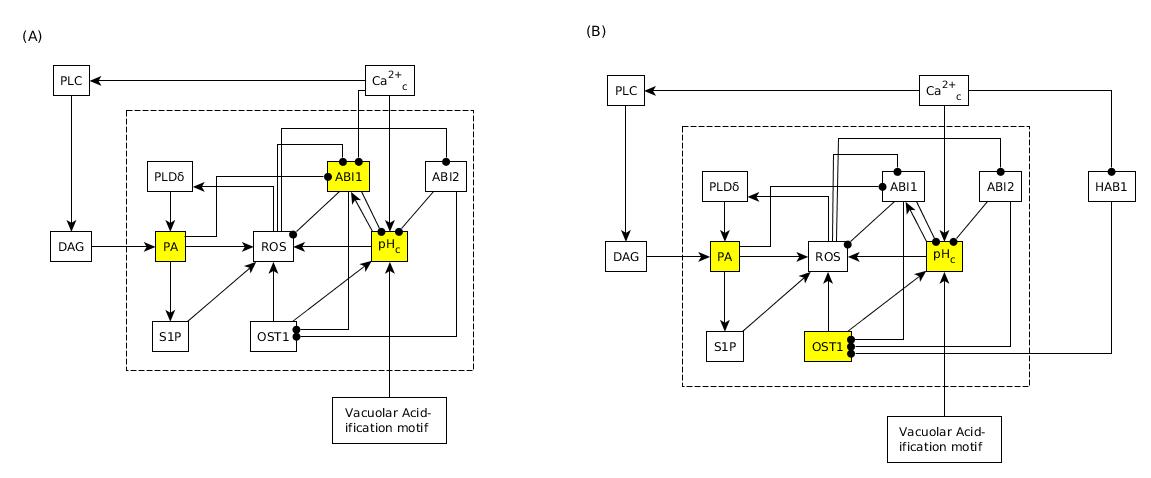


Figure S5. **Two incarnations of the stable motif associated with closure in the absence of ABA in different model versions.** The nodes shown in yellow can be stabilized in their state corresponding to the stable motif if Ca^2+^_c_ is constitutively active. (A). The stable motif when Ca^2+^_c_ inhibits ABI1. The nodes stabilized by Ca^2+^_c_ in this case, namely PA, ABI1 and pH_c_, are not sufficient to stabilize the motif. (B). The stable motif when Ca^2+^_c_ inhibits HAB1. The nodes stabilized by Ca^2+^_c_, namely PA, pH_c_ and OST1, can stabilize the motif. The third incarnation of the motif, which has Ca^2+^_c_ inhibiting ABI2, is shown in Figure 4B.

In the model version where Ca^2+^_c_ inhibits ABI1 (see Figure S5A)*,* if Ca^2+^_c_ is set to the ON state, it stabilizes PA and pH_c_ to their ON states. It also stabilizes the ABI1=OFF state. But, this three-node combination is not enough to stabilize the entire motif; hence in this case, these three nodes do not form a driver set and closure cannot be reached. Thus, this additional edge is ineffectual, consistent with the fact that the simulation results for this case are not different from those of the case where there is no additional edge to inhibit the PP2Cs. The reason for the ineffectuality of this added edge is that in the model of [2], there already are several paths by which Ca^2+^_c_ inhibits ABI1; see Figure S6.


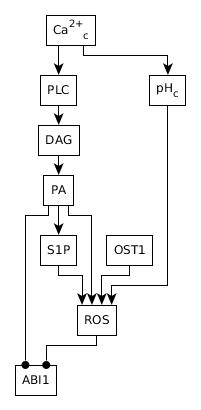


Figure S6. **The multiple paths through which Ca^2+^_c_ inhibits ABI1 in the reduced model.** OST1 contributes to the activation of ROS; it is shown as a source node in this illustration because it is not regulated by Ca^2+^_c_.

Figure 3B shows the stable motif in the model version in which Ca^2+^_c_ inhibits ABI2. In this case, if Ca^2+^_c_ is set to the ON state, it stabilizes PA=ON, pH_c_=ON and ABI2=OFF, which form a valid three-node driver, hence stabilizing the entire motif, hence leading to Closure. ROS is a single driver of the stable motif, the same way as in the absence of any added inhibition.

In the model version wherein Ca^2+^_c_ inhibits HAB1 (see Figure S5B), when Ca^2+^_c_ is set to the ON state, it stabilizes PA=ON and pH_c_=ON. It also stabilizes HAB1=OFF, which coupled with the inhibition of ABI1 by PA stabilizes OST1=ON. The three-node set PA=ON, pH_c_=ON and OST1=ON is a driver set and can stabilize the entire motif, leading to closure. The stable motif for the case when Ca^2+^_c_ inhibits PP2CA is identical, with HAB1 simply replaced by PP2CA.

In the model version wherein PA mediates the indirect inhibition of ABI2 by Ca^2+^_c_, Ca^2+^_c_ is still an external driver node of the stable motif (see Figure 4C). If Ca^2+^_c_ is set to the ON state, pH_c_ stabilizes in the ON state, PA stabilized in the ON state, and, via PA=ON, ABI2 turns OFF. Since PA=ON, pH_c_=ON and ABI2=OFF form a three-node driver, Ca^2+^_c_ can externally stabilize this motif. ROS is a single driver of the stable motif. PA and Vacuolar Acidification make up a new two-node driver set. PA inhibits ABI1 and ABI2. The inactivity of ABI1 and ABI2, coupled with Vacuolar Acidification, yields pH_c_ increase, making this two-node combination equivalent to the PA=ON, pH_c_ =ON, and ABI2=OFF three-node driver of the stable motif. Because of the logical equivalence of the Ca^2+^_c_→ PLC → DAG→PA−•ABI2 path and the direct Ca^2+^_c_ to ABI2 edge, the simulation that involve sustained high Ca^2+^_c_ concentration reach the same attractor in the model version where Ca^2+^_c_ directly inhibits the PP2Cs and in the model version where Ca^2+^_c_ inhibits the PP2Cs via PA (see Table S12). We consider various Ca^2+^_c_ activity patterns in Text S6 below.

1. Zanudo JG, Albert R. An effective network reduction approach to find the dynamical repertoire of discrete dynamic networks. Chaos. 2013;23(2):025111.

2. Albert R, Acharya BR, Jeon BW, Zanudo JGT, Zhu M, Osman K, et al. A new discrete dynamic model of ABA-induced stomatal closure predicts key feedback loops. PLoS Biol. 2017;15(9):e2003451.

3. Maheshwari P, Albert R. A framework to find the logic backbone of a biological network. BMC Syst Biol. 2017;11(1):122.
