# Supplementary material for: Model-driven discovery of calcium-related protein-phosphatase inhibition in plant guard cell signaling
Source: PLoS Comput Biol. 2019 Oct 28;15(10):e1007429. doi: 10.1371/journal.pcbi.1007429 (PMC6837631; doi:10.1371/journal.pcbi.1007429)
Supplement: S6 Text — (DOCX) [file pcbi.1007429.s022.docx]

**Text S6. Exploration of the types of Ca^2+^_c_ patterns that can drive closure in the absence of ABA in the model version where PA inhibits ABI2**

We did the same analysis of Ca^2+^_c_ pulses and repeated spikes as those described in Text S3 and observed very similar results for the model version where PA inhibits ABI2 (as shown in Figure S7A, B). Indeed, the effect of the Ca^2+^_c_ state on the stable motif associated with closure is the same in both model versions (i.e., the effect of Ca^2+^_c_ is the same whether it inhibits ABI2 by a direct or indirect mechanism). As shown in Figure 4B, C and explained in Text S4, Ca^2+^_c_ is an external driver node of both versions of the stable motif. Thus, a Ca^2+^_c_ pulse of sufficient duration, or repeated Ca^2+^_c_ spikes, in conjunction with the feedbacks internal to the stable motif, can yield the activation of the stable motif.


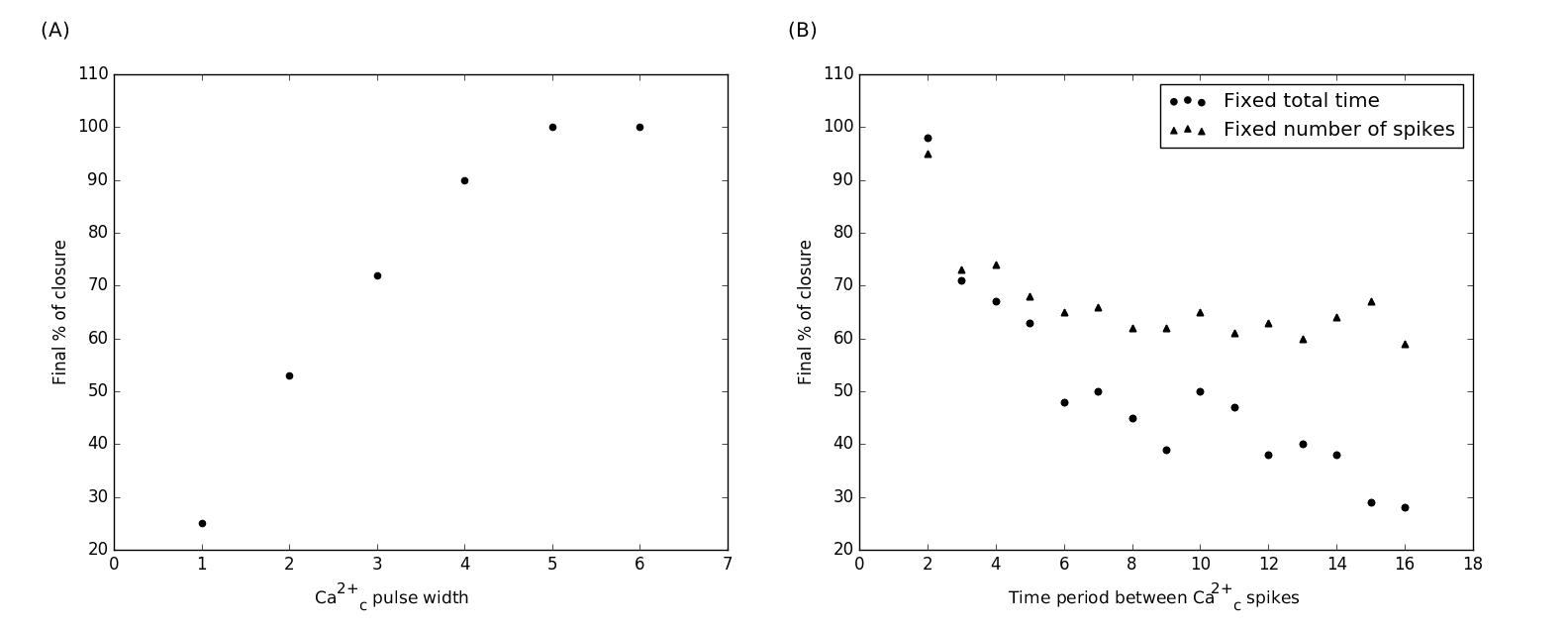


Fig S7: **Dependence of the final percentage of closure on parameters of various Ca^2+^_c_ patterns in the model version where PA inhibits ABI2.** (A). Dependence of the final percentage of closure on the width (duration) of an imposed Ca^2+^ pulse for the model version where PA inhibits ABI2. Each point reflects 500 simulations of a duration of 50 time steps. The final percentage of closure is calculated as the maximum of the percentage of closure in the last 5 time steps. The width of the Ca^2+^_c_ pulse means the time period for which Ca^2+^_c_ is fixed to the ON state and after which it is fixed in the OFF state. (B). The final percentage of closure as a function of the time between two consecutive Ca^2+^ spikes for the model version where PA inhibits ABI2. The circles indicate the cases where the total time was fixed at 50 time steps, thus there are 17 spikes if the time separation is 2 time steps and three spikes if the time separation is 16 time steps. The triangles indicate the cases where the total number of spikes was fixed at 11 and the duration of simulation was increased to accommodate these spikes. The final percentage of closure is calculated as the maximum of the percentage of closure in the last 5 time steps. Each of the data points corresponds to a set of 500 simulations.
