# Supplementary material for: Model-driven discovery of calcium-related protein-phosphatase inhibition in plant guard cell signaling
Source: PLoS Comput Biol. 2019 Oct 28;15(10):e1007429. doi: 10.1371/journal.pcbi.1007429 (PMC6837631; doi:10.1371/journal.pcbi.1007429)
Supplement: S7 Text — (DOCX) [file pcbi.1007429.s023.docx]

**Text S7. Generation and maintenance of Ca^2+^_c_ oscillations in the absence of ABA**

**Initial conditions that can lead to repeated Ca^2+^_c_ spikes in the absence of ABA**

Based on our analysis of various types of Ca^2+^_c_ patterns that can yield stomatal closure (see Text S4), we chose a parsimonious general criterion for such patterns: three or more Ca^2+^_c_ spikes (or pulses) over the 50 time steps of the simulation, including at least one spike/pulse in the last 10 time steps. We ran an ensemble of simulations of the model version where PA inhibits ABI2, in the absence of any signal, and using the initial condition where 17 nodes are initialized randomly (see Text S1). We flagged the Ca^2+^_c_ patterns that satisfy this criterion and verified that almost 100% of these flagged simulations led to stomatal closure. These results are equally valid in the model version where Ca^2+^_c_ inhibits multiple PP2Cs due to the equivalence of the role of Ca^2+^_c_.

In the presence of ABA, all simulations lead to such a Ca^2+^_c_ pattern, as expected. We observed that in the absence of ABA or of any constitutively expressed nodes, such a Ca^2+^_c_ pattern can still be generated, although only for certain initial conditions. This result is in agreement with experimental observations of spontaneous oscillations in Ca^2+^_c_ in the absence of ABA [1]. We find that the initial conditions that may result in repeated Ca^2+^_c_ spikes have at least one of six nodes, namely, cADPR, GHR1, AtRAC1, PLC, PLDδ and DAG, initialized in their state corresponding to stomatal closure, cADPR=ON, GHR1=ON, AtRAC1=OFF, PLC=ON, PLDδ=ON or DAG=ON. The first four nodes induce either Ca^2+^ influx through the membrane (CaIM) or Ca^2+^ release from intracellular stores (CIS) to increase cytosolic Ca^2+^. The nodes PLDδ and DAG are sufficient activators of the node PA, which has a chance of leading to ROS production, which in turn can induce both CIS and CaIM (see below and Figure 7A). Hence if any of these six nodes are initialized in their state corresponding to stomatal closure, there is a chance of an increase in Ca^2+^_c_. Our analysis indicates that the possibility of repeated Ca^2+^_c_ increases depends significantly on the trajectory (timecourse) of the system. Specifically, a key deciding factor is the status of positive feedback cycles incident on Ca^2+^_c_: stabilization of one of these feedbacks yields sustained Ca^2+^ spikes/pulses; otherwise any spikes are only transient and Ca^2+^_c_ finally stabilizes in the OFF state. This also provides a possible explanation for not always observing stomatal closure when spontaneous Ca^2+^ transients occur [2-4].

**Analysis of Ca^2+^ induced Ca^2+^ increase cycles**

The increase of Ca^2+^_c_ is regulated by Ca^2+^ influx through the membrane (CaIM node) and Ca^2+^ release from internal stores (CIS). According to current knowledge incorporated in the model, these two processes can independently induce an above-threshold increase of cytosolic Ca^2+^. Ca^2+^ influx through stretch-activated channels (a process that is indirectly activated by ABA in our model via ABA regulation of the actin cytoskeleton) leads to Ca^2+^_c_ increase in a Ca^2+^-independent manner. The other mechanism of CaIM, mediated by GHR1, depends on ROS and indirectly on Ca^2+^_c_, as two of the regulators of RBOH activity (PA and pH_c_) are regulated by Ca^2+^_c._ This mechanism can function in the absence of ABA, as a method of Ca^2+^ -induced Ca^2+^_c_ increase. As shown in Figure 7A, there is a positive feedback loop made up of Ca^2+^_c_, PLC, DAG, PA, ROS, GHR1, CaIM and Ca^2+^_c_. Most of the edges in this positive feedback loop represent sufficient relationships, with the exception of the PA→ ROS edge. The relationship between PA and ROS depends on the stable motif associated with closure, shown in Figure 4C and sketched in the box in Figure 7A. Simulations of the model version where PA inhibits ABI2, in the absence of ABA, indicate that this feedback loop stabilizes in all the trajectories that lead to closure, yielding a sustained ON state of PA, ROS, GHR1, CaIM (see Table S13).

There also exist two mechanisms of Ca^2+^ -induced Ca^2+^ release from internal stores, both of which can function in the absence of ABA. The two corresponding paths are Ca^2+^_c_ → PLC → InsP3 → CIS and Ca^2+^_c_ → PLC → DAG → PA → ROS → cADPR → CIS (see Figure 7A). Most of the edges in these two paths represent sufficient relationships, with the exception of the PA→ ROS edge, which in this case as well depends on the stable motif associated with closure in the absence of ABA. The nodes PLC and InsP3 have just one regulator each, namely Ca^2+^ and PLC respectively. We verified both computationally and analytically that PLC and InsP3 follow the activity pattern of Ca^2+^_c_ (oscillate if Ca^2+^_c_ oscillates and stabilize in the OFF state if Ca^2+^_c_ stabilizes in the OFF state, see Table S7, Table S13 and Text S2). However, we observed that the pulses of CIS tend to be longer than the Ca^2+^_c_ pulses/spikes, and in all cases of sustained Ca^2+^_c_ oscillations, CIS stabilizes in the ON state. In the simulations that lead to closure, CIS always stabilizes in the ON state (see Table S13). This suggests that the two pathways of Ca^2+^-induced-Ca^2+^ release from internal stores reinforce each other to yield sustained CIS.

In summary, three non-redundant positive feedback loops underlie the possibility of repeated increases in Ca^2+^_c_. PLC is a node that participates in all three loops, thus its simulated KO leads to the stabilization of Ca^2+^_c_ in the OFF state. The importance of PLC is reflected in the fact that the PLC KO simulation in the absence of ABA gives a final percentage of closure of 2% and CPC of 1.00 in the model version where Ca^2+^_c_ inhibits ABI2 and 6% and CPC of 2.51 in the model version where PA inhibits ABI2. Both of these are significantly smaller than the corresponding baseline values: percentage of closure of 22% and 30%, respectively, and CPC of 9.46 and 12.9, respectively. The final percentage of closure for PLC KO is not zero because certain initial conditions can yield the stabilization of the motif in the absence of Ca^2+^_c_ feedback (i.e. for a single increase in Ca^2+^_c_).

ROS is critical to two of these three loops. Simulated depletion of ROS yields the stabilization of Ca^2+^_c_ at 0 and a final closure percentage of zero. This strong effect is due to the fact that ROS KO makes the activation of the stable motif associated with closure, and the activation of SLAC1, impossible. Other perturbations that make the activation of the stable motif impossible, for example disruption of a positive edge to ROS, also have the same strong effect.

The node cADPR is critical to only one of these three loops. It is thus not surprising that cADPR KO gives a final percentage of closure of 17% and CPC of 7.45 in the model version where Ca^2+^_c_ inhibits ABI2 while it gives a final percentage of closure of 25% and CPC of 10.7 in the model version where PA inhibits ABI2 which are very close to the baseline values.

1. Allen GJ, Kwak JM, Chu SP, Llopis J, Tsien RY, Harper JF, et al. Cameleon calcium indicator reports cytoplasmic calcium dynamics in Arabidopsis guard cells. The Plant Journal. 1999;19(6):735-47.

2. Grabov A, Blatt MR. Membrane voltage initiates Ca2+ waves and potentiates Ca2+ increases with abscisic acid in stomatal guard cells. Proceedings of the National Academy of Sciences. 1998;95(8):4778-83.

3. Klüsener B, Young JJ, Murata Y, Allen GJ, Mori IC, Hugouvieux V, et al. Convergence of calcium signaling pathways of pathogenic elicitors and abscisic acid in Arabidopsis guard cells. Plant physiology. 2002;130(4):2152-63.

4. Young JJ, Mehta S, Israelsson M, Godoski J, Grill E, Schroeder JI. CO2 signaling in guard cells: calcium sensitivity response modulation, a Ca2+-independent phase, and CO2 insensitivity of the gca2 mutant. Proceedings of the National Academy of Sciences. 2006;103(19):7506-11.
